# A taxonomic review of the centipede genus *Scolopendra* Linnaeus, 1758 (Scolopendromorpha, Scolopendridae) in mainland Southeast Asia, with description of a new species from Laos

**DOI:** 10.3897/zookeys.590.7950

**Published:** 2016-05-17

**Authors:** Warut Siriwut, Gregory D. Edgecombe, Chirasak Sutcharit, Piyoros Tongkerd, Somsak Panha

**Affiliations:** 1 Biological Sciences Program, Faculty of Science, Chulalongkorn University, Bangkok 10330, Thailand; 2 Animal Systematics Research Unit, Department of Biology, Faculty of Science, Chulalongkorn University, Bangkok 10330, Thailand; 3 Department of Earth Sciences, The Natural History Museum, Cromwell Road, London SW7 5BD, UK

**Keywords:** Chilopoda, Scolopendra, systematics, distribution, phylogeny, species diversity

## Abstract

The centipede genus *Scolopendra* in mainland Southeast Asia is reviewed taxonomically based on morphological characters, informed by a molecular phylogenetic analysis using sequences from three mitochondrial and nuclear genes (COI, 16S rRNA and 28S rRNA). Eight nominal species of *Scolopendra*, namely *Scolopendra morsitans* Linnaeus, 1758, *Scolopendra subspinipes* Leach, 1816, *Scolopendra dehaani* Brandt, 1840, *Scolopendra multidens* Newport, 1844, *Scolopendra calcarata* Porat, 1876, *Scolopendra japonica* Koch, 1878, *Scolopendra pinguis* Pocock, 1891, and *Scolopendra dawydoffi* Kronmüller, 2012, are redescribed together with some revision of type materials. Geographical variation in each species has been compiled with reference to samples that span their distribution ranges in Southeast Asia and some parts of neighbouring areas such as East Asia, the Indian Ocean, and Africa. Comparative study of traditional taxonomic characters from external morphology provides further information to distinguish some closely related species. *Scolopendra cataracta* Siriwut, Edgecombe & Panha, **sp. n.**, is described from the southern part of Laos, with additional records in Thailand and Vietnam. The phylogenetic framework for Southeast Asian *Scolopendra* recognizes *Scolopendra calcarata* + *Scolopendra pinguis*, *Scolopendra morsitans*, and a *Scolopendra subspinipes* group that unites the other six species as the main clades. Within the *Scolopendra subspinipes* group, two monophyletic groups can be distinguished by having either slender or short, thick ultimate leg prefemora and different numbers of apical spines on the coxopleuron. *Scolopendra arborea* Lewis, 1982, is placed in subjective synonymy with *Scolopendra dehaani*. A survey of external morphology of the genital segments confirms its potential for improving species identification in *Scolopendra*. Some observations on biology and behaviour are recorded based on field surveys in this area.

## Introduction

The genus *Scolopendra* Linnaeus, 1758, is among the predominant centipede groups in tropical regions. These animals are generalist feeders that play an important role as one of the top carnivorous invertebrates in soil ecosystems. In several Asian countries, *Scolopendra* has symbolic status or figures in superstitions, and is used commercially in traditional medicine (Pemberton 1999). A few species have been proposed as model animals for medical and biological subjects (Minelli and Fusco 2004) but comprehensive work on the regional biota has not been consolidated since the last monograph on Scolopendromorpha (Attems 1930b).

The taxonomic study of *Scolopendra* dates back to the late 19^th^ to mid-20^th^ centuries (Kohlrausch 1881, Pocock 1891a, Kraepelin 1903, Attems 1930b, 1953). Several names have fallen into synonymy through the course of taxonomic revisions (Kohlrausch 1881, Kraepelin 1903, Attems 1930b). Conversely, some populations that had been classified in geographically widespread species have recently been identified as distinct species e.g., *Scolopendra antananarivoensis* Kronmüller, 2010, versus *Scolopendra morsitans* Linnaeus, 1758, or *Scolopendra subcrustalis* Kronmüller, 2009, versus *Scolopendra subspinipes* Leach, 1816. The phylogenetic position of *Scolopendra* has been investigated in the context of broad-scale phylogeny of Scolopendromorpha (Vahtera et al. 2012, 2013). Combined molecular and morphological data supported a hypothesis that Old World species of *Scolopendra* can be distinguished from the New World species, and nested most Old World species in a clade with the genus *Asanada* Meinert, 1886. Morphological discrimination between two regional groups within *Scolopendra* is made mainly based on a transverse suture on tergite 1 in New World *Scolopendra* that is absent in nearly all Old World species. Some Old World species are polymorphic with respect to external phenotypic characters. For instance, *Scolopendra subspinipes* and *Scolopendra morsitans* are cosmopolitan species worldwide, and both of them include several colour variants (Kohlrausch 1881, Attems 1930a, Shelley et al. 2005). Previous scolopendrid studies proposed that morphological variation within species is influenced by geographic distribution and ontogeny (Lewis 1968, 1972). Ontogenetic variation in colouration patterns has been recorded in *Scolopendra dehaani* Brandt, 1840 from Southeast Asia, where the species has been investigated using both molecular and morphological data (Siriwut et al. 2015a).

To date, 99 described species of *Scolopendra* have been recorded (Bonato et al. 2016), of which fourteen species have been found in the Asian tropics (Schileyko 2007, Lewis 2010b, Kronmüller 2012). Taxonomic studies have been undertaken in the following regions of Asia: the Indian Subcontinent (Jangi and Dass 1984), Indochina including Burma (Pocock 1889, 1891b, Attems 1953, Schileyko 2007), the Malay and Philippine Archipelagos (Pocock 1894, Wang 1962, 1965b, 1967a, b), and the East coast of the China Sea (Takakuwa 1942a, Chamberlin and Wang 1952, Wang 1955a, b, 1956, 1957), including Taiwan (Chao 2008). In Southeast Asia, the following *Scolopendra* species are endemics: *Scolopendra pinguis* Pocock, 1891, *Scolopendra gracillima* Attems, 1898, *Scolopendra spinosissima* Kraepelin, 1903 and *Scolopendra arborea* Lewis, 1982. There are also species which are widely distributed: *Scolopendra subspinipes*, *Scolopendra morsitans*, *Scolopendra dehaani* and *Scolopendra japonica* Koch, 1878; each of them extends into neighbouring territories such as the Indian subcontinent and the Asian temperate region (Koch 1878, Jangi and Dass 1984). In mainland Southeast Asia, *Scolopendra* comprises ten nominal species. Most of them are cosmopolitan species found synanthropically. For several species, geographical variation has not previously been documented, but we now have access to molecular evidence by which such variability can be mapped to genetic structure among and between populations.

The validity of various scolopendrid species has been ambiguous because their distributions have not been comprehensively documented and/or because the diagnostic value of particular taxonomic characters has been unclear (Lewis 1978, 2003, 2010b, Lewis et al. 2006, Edgecombe 2007). Infra-specific variation within *Scolopendra* has long been noted as a fundamental problem for distinguishing between similar species (Newport 1845, Attems 1930a, Lewis 2010b). Exclusive reliance on the traditional external morphological characters may not be sufficient to resolve some of these questions, not the least those involving problems of colour variation (Koch 1982, 1983a, Shelley 2005). The phylogenetic relationships of *Scolopendra* to other scolopendrid genera have also been contentious, as is the monophyly of the genus (Vahtera et al. 2013). Recently, evidence has been presented that morphological identification, molecular phylogeny, and geometric morphometric analyses congruently support the traditional delimitation of *Scolopendra* species in mainland Southeast Asia (Pocock 1891b, Flower 1901, Schileyko 1992, 1995, Kronmüller 2012). Molecular sequence analyses of Southeast Asian species indicated previously unrecognized groups within nominal species (Siriwut et al. 2015a). These results may indicate that even within a morphologically conservative centipede group, cryptic species can potentially be identified.

In this work, we review *Scolopendra* species in mainland Southeast Asia. The type material of some species has been re-described and, where available, type material is photographed and illustrated. All species are compared with the most closely allied congeners to provide distinguishing taxonomic characters. Variability in morphological characters is recorded in order to document geographical variation. The description of a new species is based on specimens from three SE Asian countries. Molecular phylogeny of three standard genes is analyzed, adding new samples to previous work on *Scolopendra*, to test the monophyly of each species and to determine the phylogenetic position of new species. An identification key to *Scolopendra* is presented and distribution ranges for species are updated.

## Methodology

**Material examined.** Specimens were collected mainly throughout mainland Southeast Asia, principally from Thailand, Laos, Cambodia, Myanmar, Singapore and Malaysia and kept at Chulalongkorn University Museum of Zoology, Bangkok, Thailand. Examination of additional Southeast Asian and other Oriental regional collections including available type material was based on both identified and previously undetermined specimens in several museums. All specimens were observed by using either a LEICA MZ 16A, Nikon SMZ25 or Olympus stereo-microscope. Morphological characters were photographed using montaged image stacks. Each morphological feature was serially captured with a Canon 700d linked to an automated calibration program, either Cell’D imaging or Helicon Focus on a desktop PC. In addition, illustration of some morphological variation was made by free-hand drawings.

**Institutional abbreviations**: Chulalongkorn University Museum of Zoology, Bangkok (CUMZ), Museo Civico di Storia Naturale, Genova, Italy (MSNG), The Natural History Museum, London, UK (NHMUK), Naturhistorisches Museum Wien, Vienna (NHMW), Naturhistoriska Riksmuseet, Stockholm, Sweden (NHRS), Naturhistorisches Museum Basel, Switzerland (NMB) and Zoological Museum, University of Copenhagen, Copenhagen, Denmark (ZMUC).

**Behaviour, biology and distribution.** Feeding behaviour and brooding of eggs and hatchlings was observed and photographed both in the field and the laboratory. Characteristics of habitats and brood chambers are discussed in Lewis (1981), Mitić et al. (2012) and Siriwut et al. (2014).

The distributional ranges of all SE Asian *Scolopendra* species were reinvestigated based on field sampling, museum collections, and literature records from this region. Localities cited in the descriptions are arranged geographically and are separated in two sections:

**I.** A determined locality refers to the corrected name of a locality. In cases of inaccurate spelling and outdated names on old labels, we provide the corrected name in square parentheses based on resources from the internet and/or historical notes. Spellings of new collection localities in Thailand were transcribed by the Thai Romanization program (Wirote 2001). Latitude and longitude coordinates are given for each new collecting locality, tracked by a GPS conductor via a Garmin GPS travelling device.

**II.** An undetermined locality refers to a name that is localized at only a regional scale such as by region or country.

Distribution maps for each species include the records from recent field surveys and specimens from museum collections that provide sufficiently detailed locations. Each of those localities is marked by a filled symbol. Some localities from previous taxonomic work are included using a blank symbol. All undetermined localities and some specimens which were attributed only to a region, island or country have been excluded from the distribution maps.

**Species identification and description.** Morphological terminology follows the standardized terminology (Bonato et al. 2010). Taxonomic nomenclature and species identification followed Attems (1930b, 1938, 1953), Schileyko (1992, 1995, 2007), Shelley (2005), Shelley et al. (2005), Chao (2008), Lewis (2010b), Kronmüller (2012) and Siriwut et al. (2015a). The description of the genital segments is based on previous surveys of the genital system of Scolopendridae (Demange and Richard 1969, Iorio 2003).

**Abbreviations for terminology applied to morphology used in descriptions and some comparison tables** are as follow: PS, paramedian suture; AP, apical spine; SAP, subapical spine; LS, lateral spine; DS, dorsal spine; V, ventral spine; VL, ventro-lateral spine; VM, ventro-median spine; M, median spine; DM, dorso-median spine; SP, spine on prefemoral process; ULBS, ultimate leg-bearing segment. Abbreviated terminology for the genital region used in figures is as follows: IM, intermediate membrane; AV, anal valve; GP, gonopod; LA, lamina adanalis; LS, lamina subanalis; PN, penis; SGS I, sternite of genital segment 1; SGS II, sternite of genital segment 2; TGS, tergite of genital segment.

The list of synonyms for each *Scolopendra* species follows Chilobase (http://chilobase.biologia.unipd.it/). Diagnoses are revised from Siriwut et al. (2015a), and the range of geographical variation from the type, voucher specimens and previous surveys.

**Phylogenetic reconstruction.** Southeast Asian and some temperate Asian *Scolopendra* sequences were obtained from GenBank, based on previous phylogenetic analyses (Joshi and Karanth 2011, Vahtera et al. 2013, Siriwut et al. 2015a, b). We add more *Scolopendra* sequences from additional specimens collected during 2014 from various parts of the region (Table 1). DNA extraction methods follow Siriwut et al. (2015a). Three standard genes for centipede phylogeny (cytochrome *c* oxidase subunit I, 16S rRNA and 28S rRNA) were used to reconstruct phylogenetic trees. Maximum likelihood and Bayesian inference approaches were employed, using RAxML (Stamatakis 2006) and MrBayes (Huelsenbeck and Ronquist 2001, Ronquist et al. 2012), respectively. Standard statistical tests were applied to evaluate branch support (bootstrap support and posterior probability). Algorithms and parameter settings for both analyses followed protocols detailed previously by Siriwut et al. (2015a).

**Table 1.** *Scolopendra* sequences used in phylogenetic reconstruction in present study. Abbreviation names of voucher ID codes refer to museum collections as follow: AMNH, American Museum of Natural History, New York; CUMZ, Chulalongkorn University Museum of Zoology, Bangkok; MCZ, Museum of Comparative Zoology, Harvard University, Cambridge; NHMUK, The Natural History Museum, London. References: 1 = Siriwut et al. (2015a), 2 = Siriwut et al. (2015b), 3 = Vahtera et al. (2013).

Species	Taxon locality	Voucher ID number	COI	16S	28S	Reference
*Scolopendra morsitans* Linnaeus, 1758	Khon Kaen, Thailand	CUMZ 00339	KR705662	KR705600	KR705724	1
	Nan, Thailand	CUMZ 00340	KR705661	KR705599	KR705723	1
	Chonburi, Thailand	CUMZ 00341	KR705660	KR705598	KR705722	1
	Surin, Thailand	CUMZ 00342	KR705666	KR705604	KR705728	1
	Chiang Mai, Thailand	CUMZ 00343	KR705665	KR705603	KR705727	1
	Sa Kaeo, Thailand	CUMZ 00344	KR705664	KR705602	KR705726	1
	Sisophon, Cambodia	CUMZ 00345	KR705663	KR705601	KR705725	1
	Singapore	CUMZ 00315	KR705636	KR705574	KR705698	1
*Scolopendra subspinipes* Leach, 1816	Papua New Guinea	MCZ IZ-130685	KF676528	KF676488	–	3
	Martinique	AMNH LP3879, MCZ IZ-131452	HQ402554	HQ402502	–	3
*Scolopendra cingulata* Latreille, 1829	Spain	MCZ IZ-131446	HM453310	HM453220	–	3
*Scolopendra dehaani* Brandt, 1840	Lopburi, Thailand	CUMZ 00282	KR705689	KR705627	KR705751	1
	Ayutthaya, Thailand	CUMZ 00256	KR705688	KR705626	KR705750	1
	Lan Island, Rayong, Thailand	CUMZ 00320	KR705684	KR705622	KR705746	1
	Sa Kaeo, Thailand	CUMZ 00321	KR705682	KR705620	KR705744	1
	Trad, Thailand	CUMZ 00322	KR705681	KR705619	KR705743	1
	Sichang Island, Chonburi, Thailand	CUMZ 00252	KR705683	KR705621	KR705745	1
	Chiang Mai, Thailand	CUMZ 00323	KR705659	KR705597	KR705721	1
	Chiang Mai, Thailand	CUMZ 00346	KR705658	KR705596	KR705720	1
	Maehongson, Thailand	CUMZ 00324	KR705657	KR705595	KR705719	1
	Maehongson, Thailand	CUMZ 00325	KR705656	KR705594	KR705718	1
	Sakon Nakhon, Thailand	CUMZ 00247	KR705655	KR705593	KR705717	1
	Mahasarakarm, Thailand	CUMZ 00275	KR705651	KR705589	KR705713	1
	Loei, Thailand	CUMZ 00277	KR705653	KR705591	KR705715	1
	Ubon Ratchathani, Thailand	CUMZ 00248	KR705652	KR705590	KR705714	1
	Phatthalung, Thailand	CUMZ 00274	KR705641	KR705579	KR705703	1
	Nakhon Si Thammarat, Thailand	CUMZ 00281	KR705639	KR705577	KR705701	1
	Ranong, Thailand	CUMZ 00262	KR705637	KR705575	KR705699	1
	Phang Nga, Thailand	CUMZ 00251	KR705640	KR705578	KR705702	1
	Chumphon, Thailand	CUMZ 00326	KR705638	KR705576	KR705700	1
	Uthai Thani, Thailand	CUMZ 00243	KR705632	KR705570	KR705694	1
	Prachuap Khiri Khan, Thailand	CUMZ 00327	KR705628	KR705566	KR705690	1
	Kanchanaburi, Thailand	CUMZ 00328	KR705631	KR705569	KR705693	1
	Ratchaburi, Thailand	CUMZ 00253	KR705630	KR705568	KR705692	1
	Tak, Thailand	CUMZ 00329	KR705629	KR705567	KR705691	1
	Siem Reap, Cambodia	CUMZ 00330	KR705687	KR705625	KR705749	1
	Srisophon, Cambodia	CUMZ 00331	KR705686	KR705624	KR705748	1
	Attapue, Laos	CUMZ 00332	KR705678	KR705616	KR705740	1
	Champasak, Laos	CUMZ 00333	KR705673	KR705611	KR705735	1
	Luang Prabang, Laos	CUMZ 00334	KR705677	KR705615	KR705739	1
	Phongsaly, Laos	CUMZ 00335	KR705676	KR705614	KR705738	1
	Perak, Malaysia	CUMZ 00336	KR705669	KR705607	KR705731	1
	Kelantan, Malaysia	CUMZ 00337	KR705668	KR705606	KR705730	1
	Perak, Malaysia	CUMZ 00338	KR705667	KR705605	KR705729	1
*Scolopendra multidens* Newport, 1844	Qiang Binh, Vietnam	NHMUK, MCZ IZ-131459	KF676540	KF676485	–	3
*Scolopendra calcarata* Porat, 1876	Kanchanaburi, Thailand	CUMZ 00312	KR705650	KR705588	KR705712	1
	Wat Mae Long, Mae Chaem, Chiang Mai, Thailand	CUMZ 00417	KU512629	KU512632	KU512635	This study
	Lan Sang Waterfall, Mueang, Tak, Thailand	CUMZ 00418	KU512630	KU512633	KU512636	This study
*Scolopendra japonica* Koch, 1878	Matsumoto, Japan	CUMZ 00319	KR705679	KR705617	KR705741	1
	Xieangkhuang, Laos	CUMZ 00298.1-2	KR705671, KR705670	KR705609, KR705608	KR705733, KR705732	1
	Phongsaly, Laos	CUMZ 00297.1-2	KR705675, KR705674	KR705613, KR705612	KR705737, KR705736	1
*Scolopendra pinguis* Pocock, 1891	Bo Kaeo, Laos	CUMZ 00309	KR705646	KR705584	KR705708	1
	Kanchanaburi, Thailand	CUMZ 00303	KR705646	KR705584	KR705708	1
	Nan, Thailand	CUMZ 00307	KR705644	KR705582	KR705706	1
	Xieangkhuang, Laos	CUMZ 00306	KR705643	KR705581	KR705705	1
	Huaphun, Laos	CUMZ 00304	KR705642	KR705580	KR705704	1
	Chiang Mai, Thailand	CUMZ 00313	KR705649	KR705587	KR705711	1
	Mae Hong Son, Thailand	CUMZ 00314	KR705648	KR705586	KR705710	1
*Scolopendra dawydoffi* Kronmüller, 2012	Trad, Thailand	CUMZ 00272	KR705680	KR705618	KR705742	1
	Nakhon Ratchasima, Thailand	CUMZ 00290	KR705654	KR705592	KR705716	1
	Nakhon Ratchasima, Thailand	CUMZ 00294.1-2	KR705635, KR705634	KR705573, KR705572	KR705697, KR705696	1
*Scolopendra cataracta* sp. n.	Tad E-tu Waterfall, Bolaven Plateau, Pakse, Champasak, Laos	Holotype CUMZ 00316	KR705672	KR705610	KR705734	1
	Tad-Yueang Waterfall, Mueang Singh, Luang Namtha, Laos	Paratype CUMZ 00317	KR705633	KR705571	KR705695	1
	Kao Sok National Park, Surat Thani, Thailand	Paratype NHMUK 010305528	KU512631	KU512634	KU512637	This study
*Cormocephalus monteithi* Koch, 1983	Queensland, Australia	MCZ IZ-130638	HM453309.1	AF370861.1	HM453274	3
*Digitipes kalewaensis* Siriwut, Edgecombe & Panha, 2015	Kalewa, Sagaing, Burma	CUMZ 00234	KP204116	KP204112	–	2
*Otostigmus astenus* Kohlrausch, 1878	Fiji / Vanuatu	MCZ IZ-130669/130670	HM453312	HM453221	–	3
*Sterropristes violaceus* Muadsub & Panha, 2012	Similan, Thailand	MCZ IZ-130610	KF676519	KF676477	–	3

## Results

### Phylogenetic relationships of mainland Southeast Asian *Scolopendra* and the position of *Scolopendra cataracta* sp. n.

**Figure 1.**
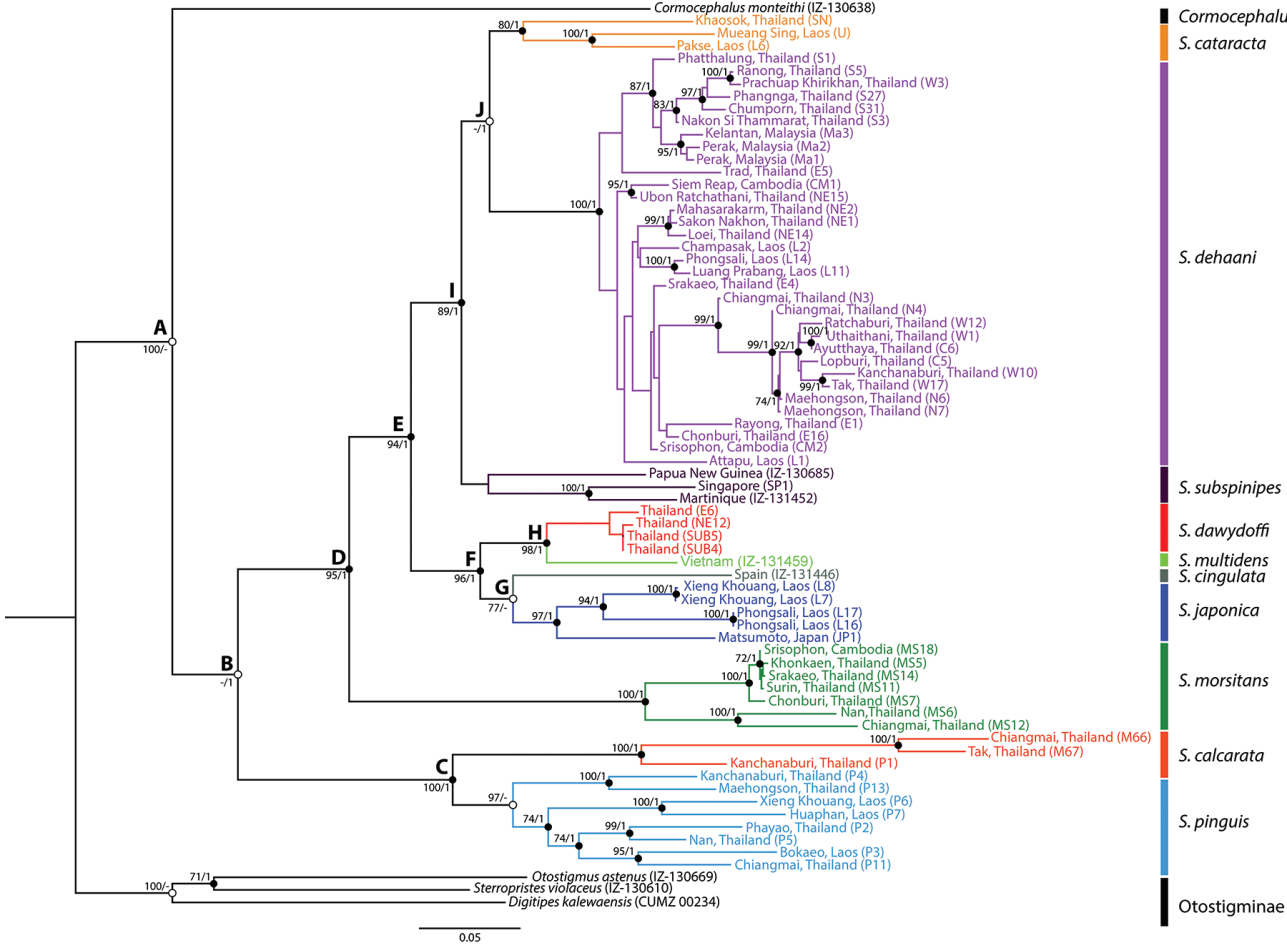
Maximum likelihood tree for *Scolopendra* in mainland Southeast Asia: colours for clades correspond to species and outgroups; black and white circles indicate statistical support values in both ML and BI analyses or only ML or BI analysis, respectively. Numbers at nodes are bootstrap support and posterior probability. Specimen codes in parentheses following localities correspond to Siriwut et al. (2015a, b: table 2) and Vahtera et al. (2013: table 1).

The phylogenetic tree from the updated concatenated DNA dataset aggregates studied specimens into eleven monophyletic groups within Scolopendrinae that are compatible with morphological identification (Fig. 1: Clade A). The phylogeny supports the monophyly of the genus *Scolopendra*, in contrast to a previous analysis in which a sampled species of *Cormocephalus* fell within *Scolopendra* (Siriwut et al. 2015a). The sequence annotation for each partial marker is given in Table 2. Genetic divergence was calculated by pair-wise comparison of k-2 parameter distance under one thousand bootstrap replicates (Table 3). The genetic distance among *Scolopendra* species ranges from 15.9–24.4% in COI and 8.3–25% in 16S (COI 13.5–16.8% and 16S 19.3–23.0% for European *Scolopendra* by Oeyen et al. (2014)). Comparing with different genera from the same/another subfamily, the distances are between 21.6–28.9% and 23.1–26.9% in COI, and 22.2–26.1% and 25.9–34.1% in 16S, respectively. Within populations, intraspecific variation is between 8.3–18.4% in COI and 5.2–11.3% in 16S (Table 4).

**Table 2.** Sequence annotation of three partial genes used in this present study.

Molecular marker	Length	Parsimony informative sites	Variable sites	Conserved sites
COI	611	260	302	309
16S	446	214	271	175
28S	683	149	188	450

**Table 3.** Genetic distance between *Scolopendra* species in mainland Southeast Asia and outgroups; upper right and lower left distance collected from COI and 16S partial gene pairwise comparisons.

Species		16S													
		1	2	3	4	5	6	7	8	9	10	11	12	13	14
COI	*Scolopendra dehaani* ^1^		0.143	0.159	0.148	0.216	0.216	0.210	0.159	0.142	0.185	0.253	0.259	0.265	0.294
	*Scolopendra cataracta* ^2^	0.159		0.164	0.138	0.238	0.213	0.199	0.156	0.158	0.175	0.259	0.262	0.288	0.300
	*Scolopendra dawydoffi* ^3^	0.171	0.190		0.164	0.209	0.220	0.217	0.123	0.127	0.083	0.222	0.292	0.285	0.293
	*Scolopendra subspinipes* ^4^	0.167	0.194	0.177		0.229	0.238	0.212	0.162	0.166	0.174	0.257	0.271	0.303	0.291
	*Scolopendra pinguis* ^5^	0.219	0.247	0.215	0.224		0.171	0.240	0.246	0.230	0.233	0.234	0.280	0.298	0.322
	*Scolopendra calcarata* ^6^	0.228	0.240	0.212	0.230	0.219		0.241	0.237	0.223	0.250	0.255	0.270	0.278	0.315
	*Scolopendra morsitans* ^7^	0.204	0.230	0.204	0.233	0.244	0.242		0.214	0.215	0.231	0.274	0.323	0.341	0.290
	*Scolopendra japonica* ^8^	0.202	0.227	0.163	0.208	0.218	0.219	0.232		0.123	0.135	0.258	0.287	0.297	0.307
	*Scolopendra cingulata* ^9^	0.188	0.197	0.177	0.214	0.224	0.228	0.221	0.181		0.131	0.261	0.274	0.291	0.322
	*Scolopendra multidens* ^10^	0.196	0.203	0.107	0.178	0.216	0.214	0.229	0.163	0.188		0.230	0.304	0.326	0.323
	*Cormocephalus* ^11^	0.236	0.263	0.234	0.238	0.269	0.239	0.267	0.243	0.231	0.233		0.266	0.296	0.322
	*Digitipes* ^12^	0.234	0.216	0.227	0.255	0.249	0.252	0.289	0.260	0.237	0.229	0.279		0.213	0.246
	*Sterropristes* ^13^	0.234	0.246	0.228	0.249	0.233	0.240	0.245	0.217	0.232	0.242	0.244	0.205		0.199
	*Otostigmus* ^14^	0.217	0.237	0.222	0.237	0.250	0.232	0.251	0.248	0.267	0.236	0.261	0.219	0.208	

**Table 4.** Genetic distance under pairwise sequence comparison within populations of *Scolopendra* species in mainland Southeast Asia.

Species	COI	16S
*Scolopendra dehaani*	0.083	0.052
*Scolopendra cataracta*	0.165	0.087
*Scolopendra dawydoffi*	0.019	0.010
*Scolopendra subspinipes*	0.146	0.109
*Scolopendra pinguis*	0.184	0.113
*Scolopendra calcarata*	0.114	0.072
*Scolopendra morsitans*	0.086	0.068
*Scolopendra japonica*	0.128	0.061

Three main clades are identified in mainland Asian *Scolopendra* (Fig. 1: Clade B), corresponding to *pinguis*-*calcarata*, *subspinipes* and *morsitans* groups (Fig. 1: Clade C, D and E). The highest intraspecific variation is observed in *Scolopendra pinguis*, a species native to this region, and the lowest variation is in *Scolopendra dehaani*, which is widespread and the dominant species in the region. The high measure of genetic divergence among *Scolopendra pinguis* populations in our previous study prompted a re-examination of those specimens and additional ones that were added in this study. The updated phylogenetic tree revealed that the former *Scolopendra pinguis* clade (Siriwut et al., 2015a: fig. 1, clade C) can be divided into two species, *Scolopendra pinguis* and *Scolopendra calcarata* (Fig. 1: Clade C), and this separation can also be supported by diagnostic morphological characters. These two species are distributed along the montane areas of Burma and Thailand and occur eastward to the Indochina sub-region in mountain ranges between Laos and Vietnam. Synapomorphic characters shared by these two species are the comparatively robust, vaulted shape of their body segments, the tergite of the ultimate leg-bearing segment being acute posteriorly, four glabrous antennal articles, and the dichromatic colouration on the cephalic plate in all *Scolopendra calcarata* specimens and most *Scolopendra pinguis* populations. In addition, the phylogeny indicates that even after re-categorising a closely related species (*Scolopendra calcarata*) that had previously been classified as an aberrant clade within *Scolopendra pinguis*, the genetic distance within *Scolopendra pinguis* is still considerable. This distance might suggest cryptic speciation among different geographical populations.

The remaining *Scolopendra* species may be divided into two groups, one consisting of *Scolopendra morsitans* and another including former subspecies of *Scolopendra subspinipes* sensu Kronmüller (2012). In the case of *Scolopendra morsitans*, monophyly is corroborated with high bootstrap support and posterior probability in ML and BI analyses, respectively (Fig. 1: Clade D). Within the *Scolopendra subspinipes* group (Fig. 1: Clade E), a clade uniting *Scolopendra cingulata* Latreille, 1829, *Scolopendra japonica*, *Scolopendra dawydoffi* and *Scolopendra multidens* (Fig. 1: Clade F) differs from a clade composed of *Scolopendra subspinipes*, *Scolopendra cataracta* and *Scolopendra dehaani* (Fig. 1: Clade I). A morphological feature shared by *Scolopendra cingulata*, *Scolopendra japonica*, *Scolopendra dawydoffi* and *Scolopendra multidens* is the *cingulata*-like ultimate legs, which have a dorsally flattened prefemur and femur and are much shorter and stouter than in the *subspinipes* clade (Fig. 1: Clade I). Attems (1938) referred to similar groupings based on form of the ultimate legs when describing *Scolopendra dawydoffi*. The number of apical spines on the coxopleural process might also be useful for discrimination of these two groups (one or two versus more than two spines in the *subspinipes* and *cingulata* groups, respectively). A monophyletic group composed of *Scolopendra subspinipes* and the two allied species (*Scolopendra cataracta* and *Scolopendra dehaani*) received statistical support both in ML and BI (Fig. 1: Clade I).

All samples of *Scolopendra cataracta* united as a clade with *Scolopendra dehaani* to the exclusion of *Scolopendra subspinipes* (Fig. 1: Clade J). This new *Scolopendra* species shares the following morphological similarities with various species of the *Scolopendra subspinipes* group: presence of two ventro-lateral spines on the ultimate leg prefemur (as in *Scolopendra subspinipes*), long and slender ultimate legs (like *Scolopendra dehaani*), and incomplete paramedian sutures on the sternites (like *Scolopendra dawydoffi*). However, *Scolopendra cataracta* is clearly distinguished from all of them by extremely short tergal paramedian sutures. Interspecific variation of DNA sequences ranges between 15.9–19.4% and 13.8–16.4% in COI and 16S, respectively, among these three related species.

### Species diversity of *Scolopendra* in mainland Southeast Asia

In this region, nine species are identified from our survey. The taxomomic boundaries between species were based on information from both morphology and molecular analysis. Two other species of *Scolopendra* were not included in this paper, namely *Scolopendra mirabilis* (Porat, 1876), and *Scolopendra hardwickei* Newport, 1845. In the case of *Scolopendra mirabilis*, an African-central Asian species, the single known specimen in SE Asia may be introduced, being found on an island in a coastal area of northern Vietnam (Schileyko 1995). Likewise *Scolopendra hardwickei* was reported from Singapore, the largest port in Southeast Asia (Decker 2013). This species has been documented from India and the Nicobar and Andaman islands, and it probably occurs in Sumatra and Java (Khanna 2001, Lewis 2010b, Decker 2013). Without further material from Singapore or neighbouring areas, the status of this recorded species in the mainland SE Asian fauna is questionable. *Scolopendra gracillima sternostriata* Schileyko, 1995, from Vietnam (Schileyko 1995, 1997), is similar to *Scolopendra pinguis* in most respects. We include it in the key below but have no new material of this subspecies, and accordingly have not revised it. The following key to native species of *Scolopendra* excludes only the two first aforementioned species.

### Key to species of *Scolopendra* in mainland Southeast Asia

**Table:** 

1	Tergite of ultimate leg-bearing segment with median suture	***Scolopendra morsitans* Linnaeus, 1758**
–	Tergite of ultimate leg-bearing segment without median suture	**2**
2	Sternal paramedian sutures complete	**3**
–	Sternal paramedian sutures incomplete	**4**
3	Coxopleural process with three or more apical spines, thick prefemur of ultimate leg with at least two ventro-lateral and four spines on prefemoral process; average ratio of width:length of ultimate leg prefemur1:2	***Scolopendra japonica* Koch, 1878**
–	Coxopleural process with 1–2 apical spines, slender prefemur of ultimate leg with two ventro-lateral and two spines on prefemoral process; ratio of width:length of ultimate leg prefemur 1:3	***Scolopendra subspinipes* Leach, 1816**
4	Ultimate leg prefemur with at least one ventro-lateral spine	**5**
–	Ultimate leg prefemur without ventro-lateral spines	***Scolopendra dehaani* Brandt, 1840**
5	Coxopleural process with one lateral and one dorsal spine; ultimate leg prefemur with numerous small scattered spines	**6**
–	Coxopleural process without lateral and dorsal spines; ultimate leg prefemur with a few enlarged spines in rows	**8**
6	Legs 21 with a tarsal spur	***Scolopendra calcarata* Porat, 1876**
–	Leg 21 without tarsal spurs	**7**
7	Sternites of anterior body segments with complete paramedian sutures	***Scolopendra gracillima sternostriata* Schileyko, 1995**
–	All sternites with incomplete paramedian sutures, reaching only 20–30% on anterior part of sternites	***Scolopendra pinguis* Pocock, 1891**
8	Complete paramedian sutures on tergites	**9**
–	Short, incomplete paramedian sutures confined to anterior and posterior parts of tergites	***Scolopendra cataracta* Siriwut, Edgecombe & Panha, sp. n.**
9	Cephalic plate and tergite 1 densely punctate; tergites with short median sulcus on posterior part	***Scolopendra multidens* Newport, 1844**
–	Cephalic plate and tergite 1 sparsely punctate; tergites without median sulcus on posterior part	***Scolopendra dawydoffi* Kronmüller, 2012**

## Systematics

### Family Scolopendridae Leach, 1816 Subfamily Scolopendrinae Kraepelin, 1903 Genus *Scolopendra* Linnaeus, 1758

#### Scolopendra morsitans

Taxon classificationAnimaliaScolopendromorphaScolopendridae

Linnaeus, 1758

**Figure 2.**
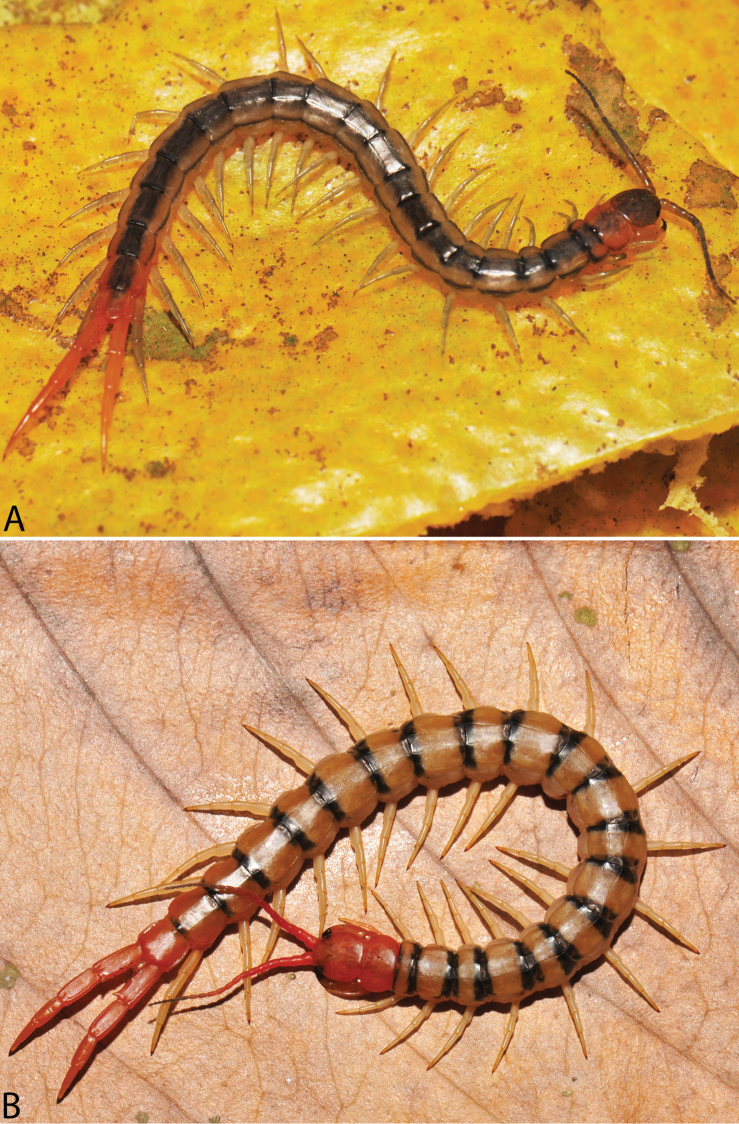
Colouration pattern during developmental stages of most *Scolopendra morsitans* populations in mainland Southeast Asia: **A** Juvenile stage **B** Adult stage (Colour morph 1).

**Figure 3.**
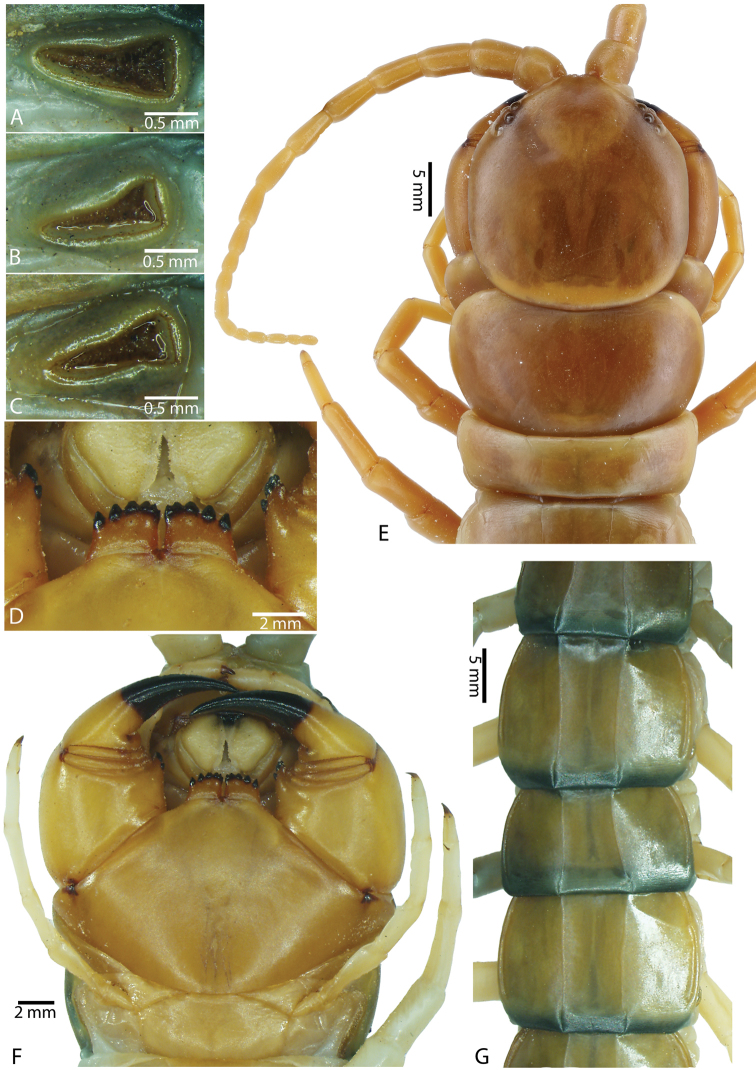
*Scolopendra morsitans* (CUMZ 00344): **A–C** Spiracles 3, 5 and 8, respectively **D** Tooth-plates **E** Cephalic plate and trunk segments 1–2 **F** Forcipular segment **G** Tergites 9–11.

**Figure 4.**
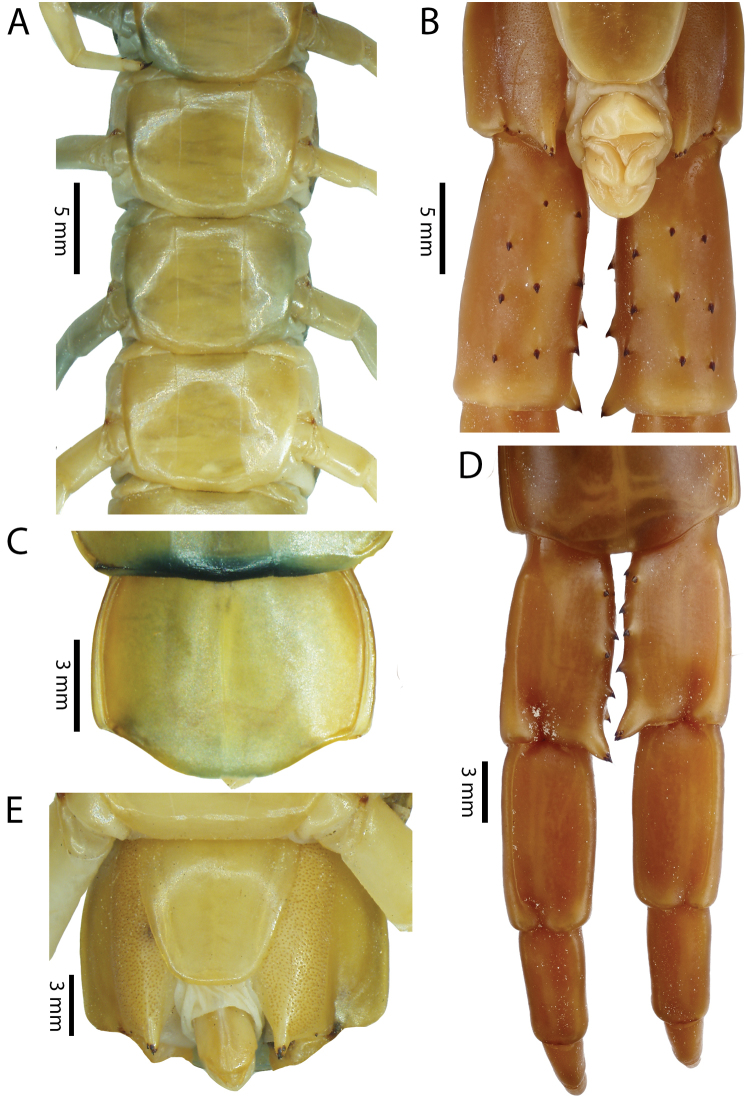
*Scolopendra morsitans* (CUMZ 00344, NHMUK 1889.7.15.14): **A** Sternites 9–11 **B** Coxopleura and ventral view of ultimate leg prefemora **C** Tergite of ultimate leg-bearing segment **D** Dorsal view of ultimate legs **E** Sternite of ultimate leg-bearing segment and coxopleura.

**Figure 5.**
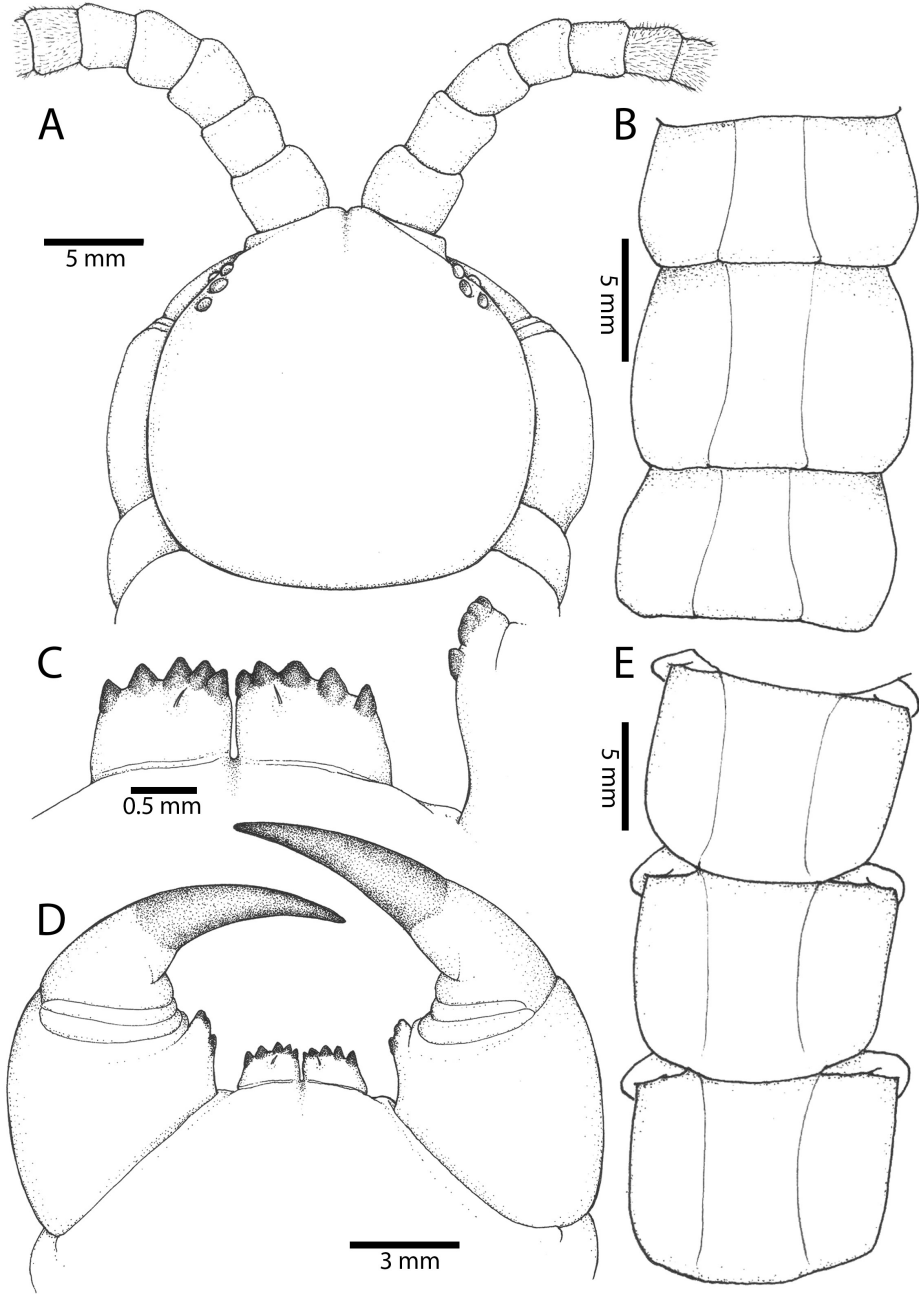
*Scolopendra morsitans* (CUMZ 00300, 00344): **A** Cephalic plate and basal antennal articles **B** Tergites 9–11 **C** Teeth on tooth-plates and trochanteroprefemoral process **D** Forcipular segment **E** Sternites 9–11.

**Figure 6.**
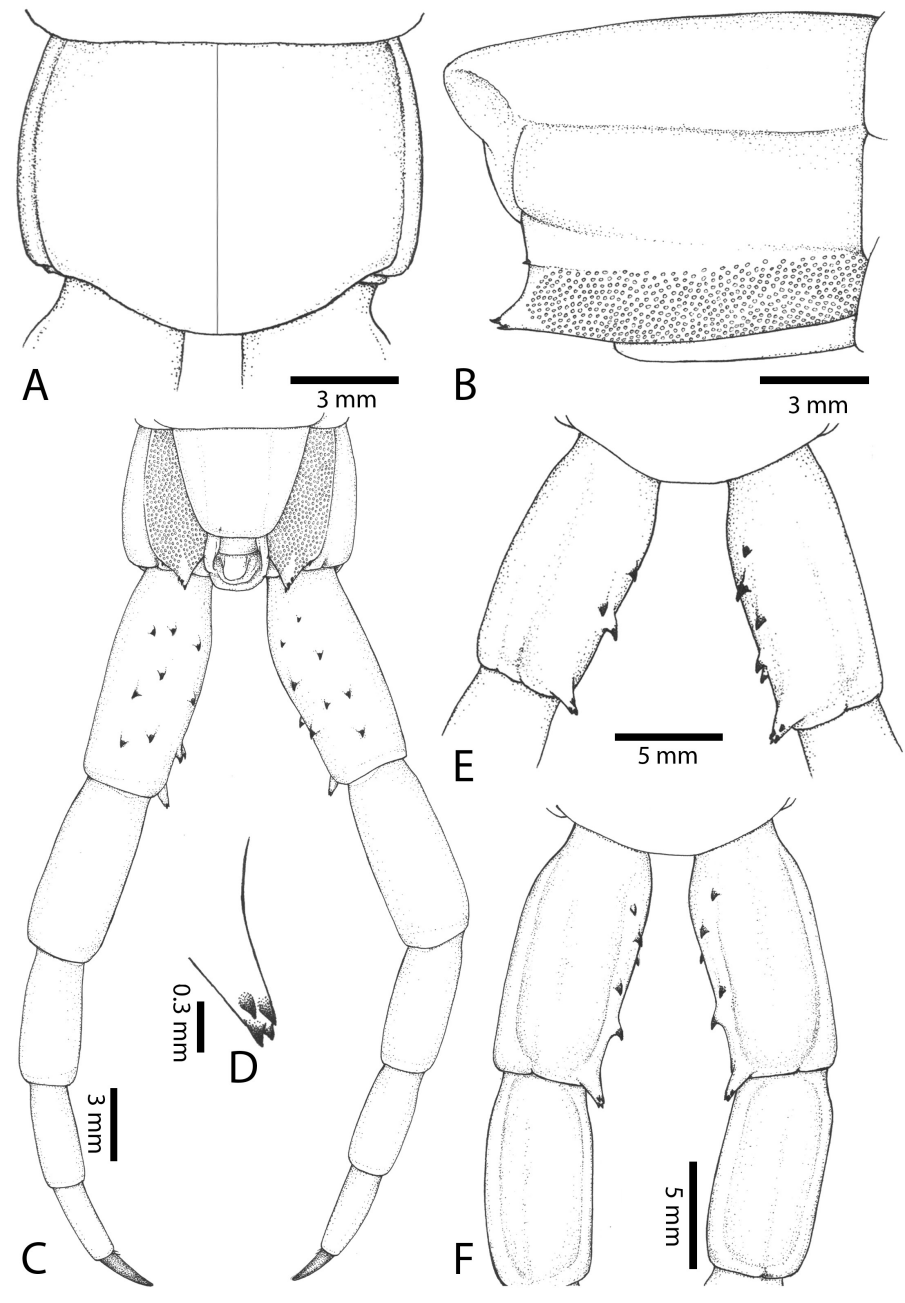
*Scolopendra morsitans* (CUMZ 00300, 00344): **A** Tergite of ultimate leg-bearing segment **B** Lateral view of coxopleuron **C** Sternite of ultimate leg-bearing segment, coxopleura and ultimate legs **D** Spines on prefemoral process of ultimate leg **E–F** Spines on ultimate leg prefemora and margination on prefemora and femora (dorsal view).

**Figure 7.**
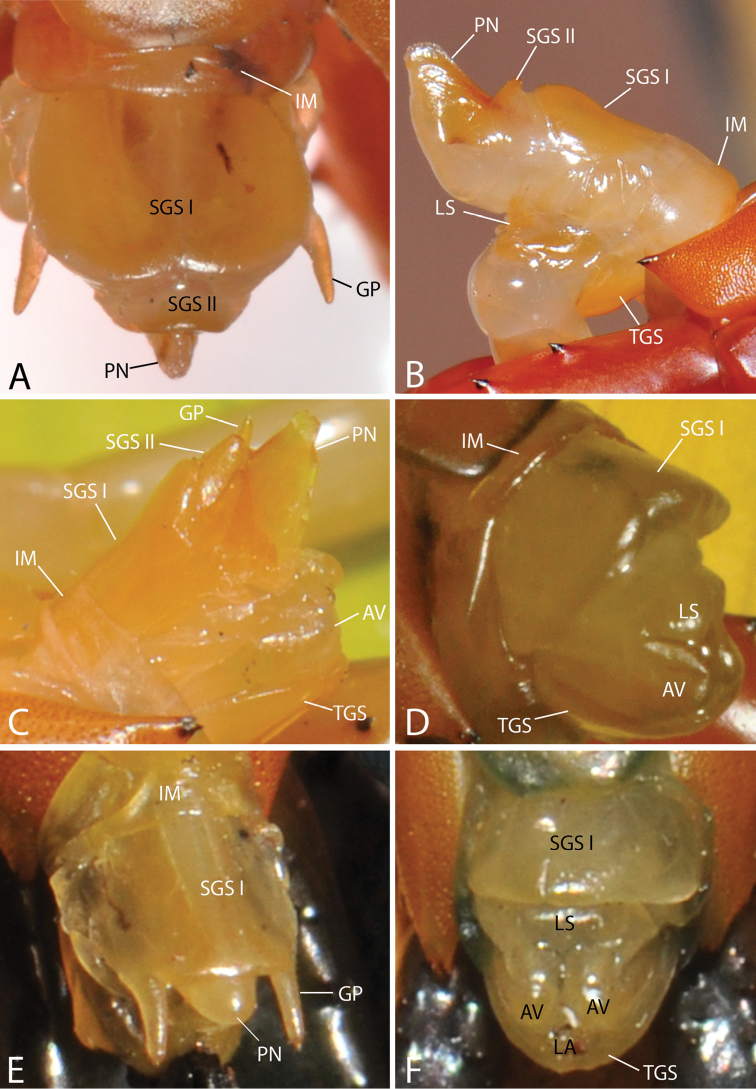
Genital segments in some live *Scolopendra* specimens: **A** *Scolopendra dehaani* (male) **B** *Scolopendra dawydoffi* (male) **C** *Scolopendra morsitans* (male) **D** *Scolopendra japonica* (female) **E** *Scolopendra pinguis* (male) **F** *Scolopendra pinguis* (female).

Figs 1 , 2 , 3 , 4 , 5 , 6 , 7

Scolopendra morsitans Linnaeus, 1758: 638. Newport 1844: 97, 1845: 378. Koch 1847: 163. Wood 1862: 23. Kohlrausch 1881: 104. Meinert 1886: 200. Haase 1887: 52, pl. 3, figs 52–54. Daday 1889: 150, 1891: 150. Silvestri 1895: 714. Kraepelin 1903: 250. Attems 1907: 80, 1909: 13, 1914a: 106, 1927: 61, 1930b: 23, Figs 38–39, 1930c: 175 1932: 5, 1938: 334. Brölemann 1912: 54. Muralewicz 1913: 200. Takakuwa 1942a: 359, 1942b: 15, 1942c: 41, 1943: 171, 1947: 936. Bücherl 1974: 107. Chamberlin and Wang 1952: 180. Jangi 1955: 597–607, 1959: 253–257. Wang 1955a: 198, 1955b: 16, 1956: 158, 1957: 27, 1962: 101, 1965: 450, 1967b: 391. Würmli 1975: 201–206. Koch 1983a: 79–91. Jangi and Dass 1984: 29, fig. 1. Lewis 2002: 81, 2010b: 107, figs 4, 33, 34. Shelley 2005: 39, figs 57–64, 2006: 5. Shelley et al. 2005: 39–58. Schileyko 2007: 75. Akkari et al. 2008: 83, map. 2. Decker 2013: 19. Tran et al. 2013: 228. Chagas-Júnior et al. 2014: 138.
Scolopendra brandtiana Gervais, 1837: 16. Newport 1845: 379.
Scolopendra bilineata Brandt, 1840: 155. Kohlrausch 1881: 108.
Scolopendra crassipes Brandt, 1840: 153. Kohlrausch 1881: 108.
Scolopendra erythrocephala Brandt, 1840: 155. Kohlrausch 1881: 108.
Scolopendra limbata Brandt, 1840: 154. Kraepelin 1903: 250.
Scolopendra platypus Brandt, 1840: 153. Newport 1844: 97, 1845: 379.
Scolopendra elegans Brandt, 1841: 21. Kohlrausch 1881: 107.
Scolopendra fulvipes Brandt, 1841: 22. Kohlrausch 1881: 108.
Scolopendra morsitans scopoliana C.L. Koch, 1841: 222, pl. 11. Akkari et al. 2008: 83.
Scolopendra angulipes Newport, 1844: 97. Kohlrausch 1881: 108.
Scolopendra leachii Newport, 1844: 97. Kraepelin 1903: 251.
Scolopendra longicornis Newport, 1844: 97. Kohlrausch 1881: 109.
Scolopendra platypoides Newport, 1844: 97. Kohlrausch 1881: 111.
Scolopendra tuberculidens Newport, 1844: 97. Kohlrausch 1881: 108.
Scolopendra algerina Newport, 1845: 387. Akkari et al. 2008: 83.
Scolopendra fabricii Newport, 1845: 384. Kohlrausch 1881: 107.
Scolopendra formosa Newport, 1845: 383. Kohlrausch 1881: 108.
Scolopendra richardsoni Newport, 1845: 385. Kohlrausch 1881: 109.
Scolopendra tigrina Newport, 1845: 381. Kohlrausch 1881: 108.
Scolopendra varia Newport, 1845: 380. Kohlrausch 1881: 111.
Scolopendra tongana Gervais, 1847: 275. Kohlrausch 1881: 110.
Scolopendra infesta Koch, 1847: 169. Kohlrausch 1881: 112.
Scolopendra planipes Koch, 1847: 168. Kohlrausch 1881: 106.
Scolopendra pella Wood, 1861: 13. Kohlrausch 1881: 111.
Scolopendra porphyratainia Wood, 1861: 15. Kohlrausch 1881: 108.
Scolopendra brachypoda Peters, 1862: 529, pl. 33, fig. 2. Kohlrausch 1881: 107.
Scolopendra mossambica Peters, 1862: 527, pl. 33, fig. 4. Kohlrausch 1881: 107.
Scolopendra compressipes Wood, 1862: 31. Haase 1887: 52.
Scolopendra modesta Wood, 1862: 29. Kraepelin 1903: 251.
Scolopendra carinipes Humbert & Saussure, 1870: 204. Kohlrausch 1881: 111.
Scolopendra afzelii Porat, 1871: 1146. Meinert 1886: 200.
Scolopendra attenuata Porat, 1871: 1148. Meinert 1886: 200.
Scolopendra chlorocephala Porat, 1871: 1149. Meinert 1886: 200.
Scolopendra cognata Porat, 1871: 1145 Meinert 1886: 200.
Scolopendra intermedia Porat, 1871: 1145. Meinert 1886: 200.
Scolopendra picturata Porat, 1871: 1144. Meinert 1886: 200.
Scolopendra pilosella Porat, 1871: 1148. Meinert 1886: 200.
Scolopendra saltatoria Porat, 1871: 1151. Meinert 1886: 200.
Scolopendra vaga Porat, 1871: 1151. Kraepelin 1903: 251.
Scolopendra wahlbergi Porat, 1871: 1150. Meinert 1886: 200.
Eurylithobius slateri Butler, 1876: 446. Kraepelin 1903: 250.
Scolopendra impressa Porat, 1876: 12. Meinert 1886: 200.
Scolopendra morsitans procera Haase, 1887: 53, pl. 33, fig. 53. Kraepelin 1903: 250.
Scolopendra morsitans sulcipes Haase, 1887: 54, pl. 33, fig. 54. Kraepelin 1903: 250.
Scolopendra morsitans calcarata Daday, 1891: 150. Kraepelin 1903: 250.
Scolopendra grandidieri Saussure & Zehntner, 1902: 302, pl. 3, fig. 13, pl. 12, fig. 6. Kraepelin 1903: 251.
Scolopendra lineata Saussure & Zehntner, 1902: 308, pl. 15, fig. 19. Kraepelin 1903: 251.
Scolopendra spinosella Saussure & Zehntner, 1902: 308, pl. 2, fig. 11. Kraepelin 1903: 251.
Scolopendra morsitans fasciata Attems, 1930a: 372. Würmli 1975: 205.
Scolopendra morsitans amazonica Bücherl, 1946: 135. Würmli 1975: 205.
Trachycormocephalus jodhpurensis Khanna, 1977: 154, figs 5–8. Jangi and Dass 1980: 67.

##### Type locality.

India.

##### Material.

**Thailand** — CUMZ 00343, one spm., Hui Hong Khrai, Chiang Mai (18°50'59.5"N, 99°13'16.4"E). CUMZ 00340, one spm., Lainan, Weing Sa, Nan (18°34'16.1"N, 100°46'59.7"E). CUMZ 00405, one spm., Wat Khao Isan, Pak Tho, Ratchaburi (13°23'2.458"N, 99°46'16.525"E). CUMZ 00302, two spms., Wat Mahavanh, Buriram (14°41'09.8"N, 102°52'33.8"E). CUMZ 00342, one spm., Ban Khok Pho, Prasat, Surin (14°32'53.4"N, 103°22'19.1"E). CUMZ 00339, one spm., Ban Dan Chang, Ta Kantho, Kalasin (16°50'06.1"N, 103°16'32.0"E). CUMZ 00409, one spm., Nong Bo, Borabue, Maha Sarakham (16°1'35.695"N, 103°7'42.487"E). CUMZ 00410, two spms., Ban Tha Tum, Mueang, Maha Sarakham (16°10'45.231"N, 103°27'4.134"E). CUMZ 00411, one spm., Wat Pa Sai Mun, Sai Mun, Yasothon (15°56'45.092"N, 104°12'1.929"E). CUMZ 00412, two spms., Wat Tham Pha Koeng, Phu Wiang, Khon Kaen (16°42'10.303"N, 102°14'56.901"E). CUMZ 00414, two spms., Wan Tham Chia, Nong Ruea, Khon Kaen (16°32'27.014"N, 102°33'19.454"E). CUMZ 00413, two spms., Phu Wiang National Park, Nong Bua Rawe, Chaiyaphum (16°41'4.089"N, 102°14'38.477"E). CUMZ 00299, one spm., Wang Bua, Kabin Buri, Prachinburi (13°57'16.3"N, 101°36'37.3"E). CUMZ 00406, one spm., Wang Bo Waterfall, Mueang, Prachin Buri (14°10'33.933"N, 101°25'40.163"E). CUMZ 00407, 16 spms., Khram Yai Island, Sattahip, Chon Buri (12°42'18.095"N, 100°50'29.35"E). CUMZ 00341, one spm., Juang Island, Sattahip, Chonburi (12°31'46.4"N, 100°57'18.4"E). CUMZ 00408, three adult and numerous juvenile spms., Ta Phraya, Sa Kaeo (14°5'1.047"N, 102°45'36.389"E). CUMZ 00300, two spms., Mueang, Sa Kaeo (13°49'07.9"N, 102°03'10.5"E). CUMZ 00344, one spm., Tha Kra Bak Reservoir, Sa Kaeo (13°58'13.9"N, 102°15'57.6"E). CUMZ 00301, one spm., Kuiburi, Prachuab Khiri Khan (12°06'32.0"N, 99°45'53.0"E). CUMZ 00403, 12 spms., Hat Wanakon National Park, Tab Sakae, Prachuab Khiri Khan (11°38'6.012"N, 99°42'5.25’’). CUMZ 00404, five spms., Kui Buri National Park, Kui Buri, Prachuab Khiri Khan (12°8'57.096"N, 99°45'34.433"E). CUMZ 00402, two spms., Ban Laem Sai, Chaiya, Surat Thani (9°24’ 9.691"N, 99°17'19.719"E). CUMZ 00400, one spm., Ching Kho, Singhanakhon, Songkhla (7°16'44.261"N, 100°31'36.759"E). CUMZ 00401, eight spms., Nhai Plao Beach, Khanom, Nakhon Si Thammarat (9°7'52.64"N, 99° 52’ 39.415"E). NHMUK 1897.9.7.29, one spm., Betong, Yala, leg. S.S. Flower. NHMUK, one spm., Ko Kraew [Khao Kaeo, Chonburi]. NHMUK, one spm., Ko Kraam [Kram Islands, Chonburi], leg. S.S. Flower, 1897–1898.

**Cambodia** — CUMZ 00345, one spm., Wat Phanombak, Srisophon (13°36'05.5"N, 102°57'09.3"E). NHMW, two spms., Mount Cardamones [Cardamom Mountain], 500 m above sea level, Mission Dawydoff, April 1893.

**Laos** — CUMZ 00419, two spms., Ban Na Ka-Som, Attapue (14°48'30.477"N, 106°50'49.948"E), CUMZ 00420, two spms., Wat Kao Kaeo, Pakse, Champasak (14°11'30.572"N, 105°54'30.821"E), CUMZ 00421, two spms., Savannhaket (16°38'25.053"N, 104°50'0.994"E)

**Myanmar** — CUMZ 00415, two spms., Old Bagan, Bagan (21°10'19.161"N, 94°51'34.61"E). CUMZ 00416, two spms., Kyaing, Pakokku (21°52'37.982"N, 94°37'47.384"E). NHMUK, one spm., Pyrimana [Pyinmana], Upper Burma. NHMUK 1889.7.15.14-18, 21 spms., Teikiyi (Rangoon) [Yangon], leg. E.W. Oates. NHMUK 1889.7.15-16, two spms., Moulmein. NHMUK 1889.7.15.17, five spms., Townwingyi (upper Burma) [Taunggyi, Shan State], leg. E.W. Oates. NHMUK 1889.7.15.15, six spms., Iharrawady [Ayeyarwady Region], leg. E.W. Oates. NHMUK 1889.7.15-20, 20 spms., Mandalay, leg. E.W. Oates (Cap). NHMW Inv. No. 671, one spm., Osl-Indien, Aracan [Rakhine State], leg. Stoliczka, 1873.

**Vietnam** — NHMUK 1926.9.30.13, one spm., Thai Nien Basin, Heure Range, Tonkin, leg. Sladen-Godman, Trust Expedition. NHMUK, one spm., Annam, with label “?19”.

**Brunei** — NHMUK 1973.7.659, one spm., Jerudong, with label “CIE Coll. A6527”. NHMW Inv. No. 641, one spm., Brunei, don. Stimdarlmus.

**Philippines** — NHMUK, one spm., Mactan, Zebu Island. NHMUK 1913.6.18.851-853, one spm., Philippines. NHMW Inv. No. 646, two spms., Manila, Novara Expedition.

**Indonesia** — NHMW Inv. No. 659, one spm., Padang, Sumatra, leg. Comal Fehiel, 1901. NHMW Inv. No. 12?, one spm., Java, with label “adeusawer 8”. NHMW Inv. No. 636, five spms, Batavia, Java, Novara Expedition, 1857–1859. NHMUK 1913.6.18.849, one spm., Celebes, with label “Spec. No. 28”. NHMW Inv. No. 632, one spm., Celebes, leg. Beruh. Walt, 3 January 1894. NHMUK, one spm., Seram [Seram Island, Maluku Province], leg. Dr. R.F. Ellen, det. D. MacFarlane, 1975. NHMW Inv. No. 635, one spm., Amboina [Ambon], leg. Doleschal, 1859.

**China** — NHMUK 1928.3.16.41-42, two spms., Amoy, leg. Prof. B. Ping 4/2/1926. NHMUK, two spms., China. NHMW Inv. No. 652, one spm., Hong Kong, April 1901. NHMW Inv. No. 638, two spms., Takao [Kaohsiung, Taiwan], leg. H. Sauler. NHMW Inv. No. 643, one spm., Fumasra [Formosa: Taiwan], leg. Breitenstein, 1884.

**Japan** — NHMUK 1913.6.18.853, one spm., Japan, leg. Koch. NHMUK 1892.10.10, one spm., Loochoo [Ryukyu Islands], leg. Holst. NHMW Inv. No. 647, two spms., Ishikagi-Jiwa, Liu Kiu Island [Ryukyu Islands], leg. H. Sauler.

**Indian and Middle Asian Territory** — NHMUK 1930.4.11.14, one spm., Ahmadabad, leg. Capt. J.B.E. Manning I.M.S. NHMUK, one spm., North Behar, Champharam, leg. Mrs. Campbell Martens with note “see letter 24/9/1930”. NHMUK, one spm., Mandras in 1925, leg. F.A. Turk. NHMUK, one spm., on porch at Bombay, leg. N.H. Soc, 1/8/1902. NHMUK 1894.10.24.70-73, five spms., Madras, leg. J.R. Henderson. NHMUK 1910.4.10.31, one spm., Ceylon, Bainbridge Fletcher’s collection. NHMUK 1903.6.18.848, one spm., Bombay. NHMUK 1948.8.6.5, one spm., Kasual [Kansal], Punjab, leg. S.F. Woodward. NHMUK 1975.12, one spm., Ceylon. NHMUK, one spm., Andaman Island, leg. B.B. Osmaston and P.A. Buxton, London School of Hygiene and Tropical Medicine. NHMUK, one spm., Northern Baluchiotan [Balochistan, Pakistan], brought to Indian Museum, leg. Prof. P.A. Buxton, Indian School of Tropical Medicine. NHMUK, one spm., Calcutta, leg. Dr. S.P.R. Chaudhuri, relocated in 25.4.49. NHMUK, one spm., Indian Ocean, with label “Assumption of J.S. Gardiner in 1952.12.17”. NHMUK, 10 spms., Aldaima, Maldives, November, 1908, det. J.S. Gardiner. NHMUK, two spms., Aldaima, Maldives, 17/12/1952. NHMUK 1952.12.17.247, one spm., Astove Island, Indian Ocean, leg. J.S. Gardiner. NHMUK 1948.10.11.8-4, five spms., Delhi, leg. J.H. Graham. NHMUK 1896.10.2.3-4, two spms., Assam, leg. E.W.P. Cambridge. NHMW Inv. No. 665, one spm., Calcutta, leg. Stoliezka, 1865. NHMW Inv. No. 650, one spm., Kamoly?, Ceylon, leg. M. Hoelui, September, 1892. NHMW Inv. No. 673, two spms., Osindien [East India], with label “Parr.”. NHMW Inv. No. 642, one spm., Kagi Island, Maldives, leg. H. Sauler.

**Africa** — NHMUK, one spm., imported with bananas from West Africa, leg. J. Knight Co. Ltd Bermondsey, Indian S.E. on 17/5/1952. NHMUK, one spm., St. Helena Island, South Atlantic Ocean. NHMUK 1892.5.16.1, one spm., Delagoa Bay [Maputo], leg. J. de Coster. NHMUK 1954.7.5.17, one female, Tanga, Tanganyika territory, Tanzania, leg. R.H.C. Sweeney. NHMUK, two spms., St. Helena, leg. A. Loveridge. NHMUK, one spm., Shinyanga [Shinyanga, Tanzania?], Tanganyika, leg. P. Gettliffe.

**Australia** — NHMW Inv. No. 637, two spms., Gayndah [Queensland], New-Holland. NHMW Inv. No. 666, one spm., Neu Holland, leg. Dr. Millas, 1884.

**South Pacific** — NHMUK 1893.11.15.1-2, two spms., Tongatapu. NHMUK 1975.65, one spm., Samoan Island [Samoa]. NHMUK 1966.72, two spms., Samoan Island. NHMUK 1892.12.27.11-13, two spms., Levuka, Fiji, leg. H. Hjorring, det. G.M. Thomson. NHMUK 1913.6.18.850, one spm., Vamma Levu [Vanua Levu, Fiji]. NHMUK 1976.31, two spms., Samoan Island. NHMW Inv. No. 634, one spm., Tahiti. NHMW Inv. No. 649, one spm., Tahiti, Museum Goddefroi, 3/3/1881.

**Undetermined locality** — NHMW Inv. No. 640, one spm., Yantempo, leg. H. Sauler. NHMUK 1985.29, three spms., with label “Lnas”.

##### Diagnosis.

17–23 antennal articles, 5–8 basal articles glabrous dorsally. Each tooth-plate with 5–6 teeth. Tergites 7(12)-20 with paramedian sutures. Tergite of ultimate leg-bearing segment with median suture. Complete paramedian sutures on sternites 2–20. Coxopleural process with 3–4 apical and 0–1 lateral spines. Ultimate leg prefemora with three rows of ventral spines (2–6 VL, 3 V, 2–6 VM), 2–6 M, 2–6 DM and 0–8 spines on prefemoral process. One tarsal spur on legs 1–19 (in Southeast Asia).

##### Composite description.

Body length up to 12.7 mm (In Australian populations according to Koch (1983a)). Reddish-brown or yellowish colour on body segments. Cephalic plate and tergites monochromatic or dichromatic in adult (Fig. 2B). Tergites usually reddish-orange (Thai, Laos and Cambodian populations); dark band on posterior border of tergites. Cephalic plate with or without small punctae, median sulcus present on anterior part. Posterior part of cephalic plate without paramedian sutures.

**Figure 8.**
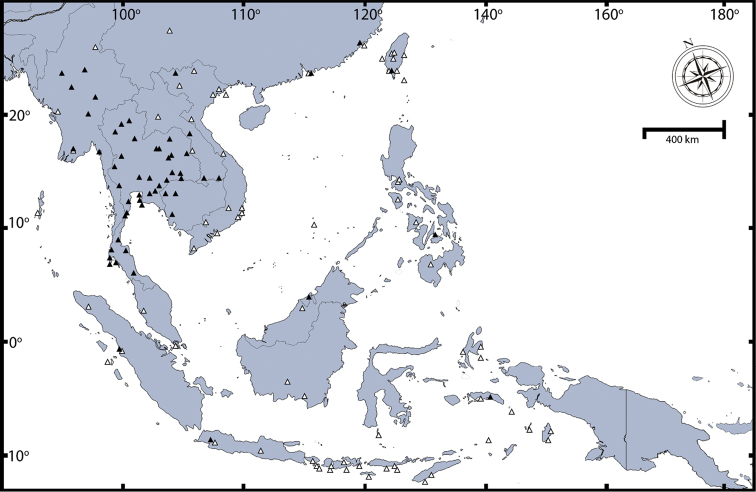
Distribution map of *Scolopendra morsitans* in Southeast Asia: Filled triangles indicate data from material examined herein; blank triangles indicate localities in the literature (Shelley et al. 2005, Schileyko 2007).

Antenna usually with 18–20 articles (sometimes 17, 21 or 23 on one side), basal 5–7 glabrous dorsally (Figs 3E, 5A), 5–8 articles glabrous ventrally. Antennae reach to segment 4. Forcipular trochanteroprefemoral process (Figs 3F, 5D) with denticles in two groups, 2–3 apical and one inner. Tooth-plates wider than long or nearly as long as wide, usually 5–6 teeth (Fig. 3D); rarely 3, 4 or 7. Tooth-plate with straight, transverse basal suture (Fig. 5C). Coxosternite without median suture. Article 2 of second maxillary telopodite with spur.

Anterior margin of T1 underlying cephalic plate (Fig. 3E). Complete paramedian sutures from TT4–5; margination typically starting on T14 (one spm., with margination restricted restricted to last two tergites). Tergite surface (Figs 3G, 5B) smooth. Tergite of ultimate leg-bearing segment (Figs 4C, 6A) curved posteriorly, with median suture; ratio of width: length of tergite of ultimate leg-bearing segment 1.34:1. Sternites (Figs 4A, 5E) with complete paramedian sutures. Sternites without depressions. Sternite of ultimate leg-bearing segment (Fig. 4E) with sides converging posteriorly; surface without depression. Pore-field on coxopleuron terminating well beneath margin of tergite of ultimate leg-bearing segment, pore area slightly widened anteriorly.

Coxopleural process moderately long or short, usually with 4–5 apical and 0–1 lateral spines (Fig. 4E); pore-free area extending 40–50% length from distal part of coxopleural process to margin of sternite of ultimate leg-bearing segment (Fig. 6B).

All legs without setae and tibial spurs. One tarsal spur on legs 1–19 (20 in some African and Indian populations). Ultimate legs: thick and moderately long, with ratios of lengths of prefemur and femur 1.2:1, femur and tibia 1.3:1, tibia and tarsus 2 1.7:1.; tarsus 1 and tarsus 2 2.8:1. In male, lateral margin of prefemora, femora and tibia marginated dorsally. Prefemoral spines (Figs 4B, D, 6C–F): 2–4 VL, 3 V, 2–4 VM, 2–3 M, 2–3 DM and prefemoral process usually with 3–5 spines. Posterior margin of prefemur with shallow median groove.

Genital segments well developed, reaching longer than the distance between posterior margin of sternite of ultimate leg-bearing segment and distal part of coxopleural process (Fig. 7C). Sternite of genital segment 1 round and convex posteriorly, with median suture. In male, sternite of genital segment 2 attached to penis. Tergites of genital segments without small setae. Gonopods with small setae in male. Penis with fine posterior seta.

**Colouration.** *Scolopendra morsitans* demonstrates colour variation among its populations in SE Asia. Previously, colour variation has been recorded in African, Australian and Taiwanese populations (Lewis 1968, Koch 1983a, Chao 2008), those studies proposing that latitude and habitat composition might affect this variability. Recent molecular analyses of Thai-Cambodian *Scolopendra morsitans* suggested that some colour morphs may be specific to local populations (Siriwut et al. 2015a), although similar patterns occur in each of three different continental faunas. We have recorded the colouration pattern in juvenile and adult specimens (Fig. 2A–B, respectively):

**Colour morph 1.** Dichromatic. Cephalic plate, T1 and tergite of ultimate leg-bearing segment orange, the remaining tergites brownish. Posterior borders and lateral margins of tergites dark. Antenna bright orange. Pleuron of leg-bearing segments with pale grey integument, pleurites orange. Legs 1–21 orangish or yellow. Ultimate legs orangish or light brown.

**Colour morph 2.** Dichromatic. Cephalic plate, T1 and tergite of ultimate leg-bearing segment dark brown or blackish, the remaining tergites brownish. Posterior borders and lateral margins of tergites dark. Antenna dark blue. Pleuron of leg-bearing segments with pale grey integument, pleurites orange or brown. Legs 1–20 yellowish or pale. Ultimate legs blackish or brown.

##### Discussion.

*Scolopendra morsitans* is morphologically varied and subsumes many synonyms that are now attributed to geographical and/or ontogenetic variation. Intraspecific variation has been studied in Africa (Lewis 1969), India (Jangi 1955, 1959), and Australia (Koch 1983a), revealing that some diagnostic characters are inconsistent within its populations. These include: number of glabrous antennal articles, number of teeth on the forcipular tooth-plates, number of tergites that are marginated, and the number of legs with tarsal spurs. This species also demonstrates differences in colour patterns that might be correlated with its geographical distribution. Lewis (1969) noted that a population of *Scolopendra morsitans* from Bihe, Angola, demonstrated a dark body with red legs whereas specimens from Sudan were straw-coloured. Here we record two colouration patterns in Thai populations that do not occur sympatrically. In addition, some morphological characters might be restricted to certain geographical populations, such as a tarsal spur on leg 20, which has been reported from India and in some African populations. For this reason, the utility of this character for defining boundaries between *Scolopendra morsitans* and other *Scolopendra* species that share some morphological characters with it, such as *Scolopendra laeta* Haase, 1887 and *Scolopendra antananarivoensis*, is not absolutely clear. Our survey of geographic variation in *Scolopendra morsitans* is presented in Table 5.

**Table 5.** Geographical variation in several populations of *Scolopendra morsitans* in Old World territory including Australia. ? insufficient data.

Character	India^3^	Burma^1^	Indochina^1,2^	Malay Archipelago^1,2^	Philippines^1^	East Asia^1,6^	Australia^5^	Africa^2,4^
Number of antennal articles	19–20	18–22	20–21	18–20	17–19	18–20	17–23	17–21
Number of glabrous articles	6–9	6–7	5	6–7	6–7	5–7?	3–8	5–7
Teeth on tooth-plate	5	4–5	3–7	4–5	4–5	5	3–6	5
First tergite with complete paramedian sutures	2	3	4–5	2–3	2–3	3	2–4	2–4
First tergite with margination	7–17	6–17	5–14	6–13	12–13	10–14	5	2–7 (15)
Tergite surface	smooth	smooth	smooth	smooth	smooth	smooth	smooth	smooth
Median furrow on tergite of ULBS	present	present	present	present	present	present	present	present
Paramedian sutures on sternites	incomplete	incomplete	incomplete	incomplete	incomplete	incomplete	incomplete	incomplete
Sternite of ULBS	?	without depression	without depression	without depression	without depression	without depression	without depression	?
Spines on coxopleural process	AP: 3–5 LS: 0–1	AP: 3–5 LS: 1	AP: 1–5 SAP: 0–1 LS: 0–1	AP: 3–5 LS: 0–1	AP: 4–5 LS: 0–1	AP: 4 LS: 0–1	AP: 2–6	AP: 2–6 LS: 0–1
Spine formula on prefemora of ultimate legs	VS: 3 rows DS: 4–5 2 rows (4–5) SP: 4–10	V: 5–10 (3 rows) M: 2–4 DM: 2–4 SP: 3–7	V: 5–10 (3 rows) M: 0–6 DM: 0–5 SP: 2–6	V: 5–10 (3 rows) M: 2–4 DM: 2 SP: 3–7	V: 9–10 (3 rows) M: 2–4 DM: 2 SP: 3–7	V: 7–9 (3 rows) M: 0–3 DM: 2–6 SP: 0–4	VL: 2–6 VM: 2–8 M: 2–6 DM: 2–6 SP: 4–8	VL: 6–12 M: 2–6 DM: 3–6 SP: 3–8
Legs with one tarsal spur	1–19(20)	1–19	1–19	1–19	1–19	1–19	1–19	1–19(20)

**Note**: each superscript number refers to description in previous and present studies as follow; ^1^ = this study, ^2^ = Attems (1930b, 1953), ^3^ = Jangi and Dass (1955), ^4^ = Lewis (1967, 1969), ^5^ = Koch (1983), ^6^ = Chao (2003, 2008).

Our phylogenetic analysis corroborates the monophyly of SE Asian populations of this species (Fig. 1). Previous molecular phylogenetic analyses of the *Scolopendra morsitans* complex in India suggested that *Scolopendra morsitans* was paraphyetic with respect to specimens that were determined as *Scolopendra amazonica*, the latter name being used for specimens with tarsal spurs on leg 20 (Joshi and Karanth 2011). From these results, it seems that molecular phylogenetics of this species complex throughout its geographic range may be necessary to clarify the taxonomic value of some variable morphological characters and to more confidently determine the taxonomic status of some phenotypically similar species. It is likely that some names currently treated as junior subjective synonyms of *Scolopendra morsitans* may be found to be applicable to cryptic species.

##### Distribution.

This is one of the oldest described centipede species and it is distributed worldwide in the tropics.The native distribution is difficult to determine because of assumed introduction in several areas. Shelley et al. (2005) provided full distribution records. Here we provide a distribution map (Fig. 8) and summarise the occurrence of *Scolopendra morsitans* in Southeast Asia and some parts of East Asia as follows: **Southeast Asia**: Thailand (entirely), Laos (southern part; Khammouane, Champasak), Cambodia (probably entirely), Vietnam (fide Schileyko 2007: Bai Tu Long Archipelago, Nghe An (Vinh), Thua Thien Hue (Hai Van Pass), Dak Lak, Khanh Hoa, Ninh Thuan (Phan Rang), Lam Dong (Da Lat), Ba Ria, Tay Ninh and Ca Mau), Spratly Archipelago, Myanmar (probably entirely), Malaysia, Singapore, Indonesia, Philippines (Manilla and Zebu Island), and Brunei (Jerudong). **East Asia**: China (Amoy, Hong Kong, Taiwan) and Japan (Ryukyu Islands).

#### Scolopendra subspinipes

Taxon classificationAnimaliaScolopendromorphaScolopendridae

Leach, 1816

Figs 9A , 10 , 11 , 12 , 13 , 14 , 15 , 16 , 17 , 18

Scolopendra subspinipes Leach, 1816: 383. Newport 1844: 96. Koch 1847: 163. Kohlrausch 1881: 96. Meinert 1886: 202. Haase 1887: 44, pl. 3, Figs 43–45. Daday 1889: 150. Latzel 1892: 185. Pocock 1894: 312. Silvestri 1894: 624, 1895: 714. Attems 1897: 477, 1903: 81, 1914a: 106, 1914b: 568, 1914c: 380, 1915: 2, 1927: 1, 1930b: 29, fig. 43, 1930c: 175, 1932: 5, 1938: 334; 1953: 145. Flower 1901: 21. Ribaut 1912: 248. Chamberlin 1918: 158, 1920a: 30, 1920b: 391. Muralewicz 1913: 201. Chamberlin and Wang 1952: 190. Takakuwa 1942a: 1, 1942b: 15, 1942c: 41, 1943: 171. Wang 1955b: 16, 1962: 101, 1965a: 449, 1967b: 391. Würmli 1972: 91, Fig. 1. Shelley 2000: 42. Lewis 2002: 83, 2007: 10, 2010a: 129, 2010b: 111, 2010c: 380, figs 1–3. Schileyko 1995: 77, 2007: 75. Chao and Chang 2003: 4, fig. 9, tables 1, 2. Chao 2008: 35, figs 37–39, table 2. Kronmüller 2012: 20, table 1, figs 3–5. Chagas-Júnior et al. 2014: 139.
Scolopendra audax Gevais, 1837: 50. Kohlrausch 1881: 99.
Scolopendra septemspinosa Brandt, 1840: 152. Kraepelin 1903; 256.
Scolopendra borbonica Blanchard, 1829: 7, pl. 1. Kohlrausch 1881: 98.
Scolopendra sexspinosa Newport, 1844: 96, 1845: 391. Kohlrausch 1881: 100. Daday 1891: 149.
Rhombocephalus gambiae Newport, 1845: 392. Kraepelin 1903: 256.
Scolopendra ceylonensis Newport, 1845: 391. Kohlrausch 1881: 98.
Scolopendra flava Newport, 1845: 392. Kohlrausch 1881: 98.
Scolopendra gervaisii Newport, 1845: 390. Kohlrausch 1881: 100.
Scolopendra lutea Newport, 1845: 392. Kohlrausch 1881: 99.
Scolopendra ornata Newport, 1845: 392. Koch 1863: 10, pl. 66, fig. 134. Kohlrausch 1881: 100.
Scolopendra placeae Newport, 1845: 390. Kohlrausch 1881: 100.
Scolopendra planiceps Newport, 1845: 391. Kohlrausch 1881: 99.
Scolopendra rarispina Gervais, 1847: 270. Kohlrausch 1881: 97.
Scolopendra sandwichiana Gervais, 1847: 276. Kohlrausch 1881: 99.
Scolopendra mactans Koch, 1847: 16, 1863: pl. 41, fig. 79. Kohlrausch 1881: 98.
Scolopendra sulphurea Koch, 1847: 156, 1863: 24, table. 11, fig. 21. Kohlrausch 1881: 98.
Scolopendra byssina Wood, 1861: 10. Kohlrausch 1881: 99.
Scolopendra cephalica Wood, 1861: 12. Kraepelin 1903: 256.
Scolopendra cephalica gracilis Wood, 1861: 13. Kraepelin 1903: 256.
Scolopendra dinodon Wood, 1861: 12. Kohlrausch 1881: 98.
Scolopendra gracilipes Wood, 1861: 12. Kraepelin 1903: 256.
Scolopendra parvidens Wood, 1861: 13. Kohlrausch 1881: 98.
Scolopendra plumbeolata Wood, 1861: 14. Kohlrausch 1881: 97.
Scolopendra bispinipes Wood, 1862: 28. Brölemann 1909: 25.
Scolopendra nesuphila Wood, 1862: 31. Kraepelin 1903: 256.
Scolopendra repens Wood, 1862: 31. Kraepelin 1903: 256.
Scolopendra elongata Porat, 1871: 1143. Meinert 1886: 202.
Rhombocephalus smaragdinus Butler, 1876: 446. Kraepelin 1903: 256.
Scolopendra damnosa Koch, 1878: 789. Kraepelin 1903: 256.
Scolopendra mutilans Koch, 1878: 791. Haase 1881: 47, pl. 3, fig. 47. Takakuwa 1947: 938.
Scolopendra aurantiipes Tömösváry, 1885: 67. Haase 1887: 44. Takakuwa 1936: 152.
Scolopendra variispinosa Tömösváry, 1885: 67. Haase 1887: 44. Takakuwa 1936: 152.
Scolopendra rugosa Meinert, 1886: 202. Kraepelin 1903: 257.
Scolopendra meyeri Haase, 1887: 49, pl. 3, fig. 50. Kraepelin 1903: 257.
Scolopendra macracanthus Bollman, 1889: 213. Kraepelin 1903: 257.
Scolopendra flavicornis Tömösváry, 1885: 67. Kraepelin 1903: 256. Takakuwa 1936: 152.
Scolopendra subspinipes gracilipes Daday, 1891: 149. Kraepelin 1903: 256.
Scolopendra subspinipes molleri Verhoeff, 1892: 199. Kraepelin 1903: 256.
Scolopendra polyodonta Daday, 1893: 5. Kraepelin 1903: 257.
Scolopendra machaeropus Attems, 1901: 136. Kraepelin 1903: 257.
Scolopendra aringensis Sinclair, 1901: 529, pl. 31, fig. 46, pl. 32, Figs 67, 85, 86, 93. Kraepelin 1903: 257.
Scolopendra subspinipes mutilans Kraepelin, 1903: 263. Attems 1938: 334, 1953: 138. Takakuwa 1943: 171. Takashima 1952: 4. Shinohara 1961: 75. Wang 1993: 850, fig. 5. Schileyko 1998: 268, 2007: 75. Chao and Chang 2003: 8, table 1–2, figs 6–7. Chao 2008: tab. 2. Lewis 2010b: 111. Kronmüller 2012: 20, table 1.
Scolopendra subspinipes gastroforeata Muralewicz, 1913: 201. Lewis 2010b: 114. Kronmüller 2012: table 1.
Scolopendra subspinipes piceoflava Attems, 1934: 51. Lewis 2010b: 113. Kronmüller 2012: 21, table 1.
Scolopendra subspinipes fulgurans Bücherl, 1946: 148, 1974: 107. Kronmüller 2012: 21, table 1.

##### Type locality.

Not designed. The whereabouts of the holotype are unknown.

##### Material.

**Specimens referred to *Scolopendra subspinipes* Leach, 1816: Malaysia** — NHMUK 1897.1.25.12, one spm., Penang, Malaysia, leg. S.S. Flower, 26/11/1896, with label “PENANG 226”. NHMUK, two spms., Penang, Malay Peninsula, leg. H.N. Ridley. NHMUK (E): 2000-110, one spm., C89, caught in base camp, Mulu, Sarawak, 5/8/1978, leg. J.G.E. Lewis. NHMUK (E): 2000-110, one spm., Mulu, Sarawak, 21/8/1978, leg. Ian Baillie. NHMUK 1952.9.8.576, one spm., Sarawak, Borneo, with label “F.42.24.8.1932”, Oxford University Sarawak Expedition. NHMUK 1906.2.18.3, one spm., Malay Peninsula, leg. Annandale and Robinson, with label “No. 45”.

**Singapore** — CUMZ 00315, one spm., Kentridge Road, Singapore (1°17'08.9"N, 103°47'09.8"E). NHMUK 1886.115, one spm., Singapore, leg. Dr. Invine Russell, don. E.W. Holmes Zgar. NHMUK 1897.12.22.63-64, two spms., Singapore, leg. S.S. Flower.

**Indonesia** — NHMUK 1893.5.13.30, two spms., east coast Sumatra, leg. Mrs. Findlay. NHMW, 12 spms., Singkarak, Klakah [Singkarak Lake, Sumatra, Indonesia]. NHMUK, one spm., Java, with label “No. 46/108”, 2/3/1885. NHMUK 1874.57, two spms., Java, leg. G. Lyon Esq. NHMW Inv. No. 726, six spms., Java, Indonesia, 1884, det. Attems C. NHMUK 1896.6.20.33, one spm., Surabaya, Java, leg. S.S. Flower, April 1896. NHMW Inv. No. 8596, two spms., Klakah, Lumajang Regency, East Java, Indonesia, leg. Thienemanm, October/November 1928, det. Attems C. NHMW Inv. No. 965, two spms., Java, 30/6/1857-1859, Novara expedition. NHMUK, one spm., found between base camp and corner camp near Utakawa River, Expedition to Dutch New Guinea [Irian Jaya]. NHMUK 1911.12.23.63-64, two spms., Muisika River, South Dutch New Guinea, B.O.U. expedition, leg. Mr. Wollaston.

**Philippines** — NHMUK 1896.3.8.87, one spm., Philippines, leg. Pascal. NHMUK 1913.6.18.897, one spm., Philippines, with label “Typical form” and “Spec. 13”. NHMUK 1883.33, two spms., Manila, leg. S.W. Taylor.

**China** — NHMUK 1928.3.16.64-68, one spm., Amoy, China, leg. Prof. C. Ping, 4/2/1926, with label “No.CAT.3”. NHMUK 1894.12.20.1, one spm., Central Formosa [Taiwan], leg. Holst. NHMUK, one spm., Changsha, Siang River (28°12'N, 112°59'E), leg. L.T. Loomer and R.H.S. Rodger R.N.

**Japan** — NHMUK, one spm., Japan, leg. Koch, with label “Number 2?”. NHMUK 1907.6.18.1-2, two spms., Goto Island, Japan, leg. R. Gordon Smith.

**South Asia** — NHMUK, one spm., India, with label “No. 45/29”. NHMUK, two spms., Ceylon, with label “No.46/104”.

**Africa** — NHMUK 1881.99, one spm., Kee Road, South Africa

**Jamaica** — NHMUK, two spms., in bananas from Jamaica, det. P.C. Jerrard. NHMUK, seven spms., Jamaica. NHMUK Entomology: 2000-110, one spm., in case of bananas from Jamaica, 1987. NHMUK, one spm., in bananas from Jamaica, Longford, Kent, leg. Miss S. Truman, 1/11/1950.

**Madagascar** — NHMUK, one spm., West Africa, with label “n/a”. NHMUK 1989.3.12.3-7, five spms., Madagascant [Madagascar], det. Lewis and Ransome (cap.). NHMUK 1878.30, one spm., Madagascar.

**Rodrigues** — NHMUK Entomology: 2000-110, six spms., under stones, heavily grazed grassland hill, west of port Mathunn, Rodrigues, 9/4/1995 (63°25'E, 9°41'S). NHMUK Entomology: 2000-110, three spms., Solitude expedition to Rodrigues, 10.11.1995. NHMUK Entomology: 2000-110, two spms., under forest cavern, Patate, Rodrigues (63°23.5'E, 19°45.5'S). NHMUK, six spms., Rodrigues, leg. Slater, October 1876, with label “refer to *Scolopendra mossambica* (Peter)”. NHMUK, one spm., probably from Rodriguez, with label “Rodriguez: Anse aux anglaise”. April 1983 CIE A14995”. NHMUK 1924.2.9.6-11, six adult spms. and brood, Rodrigues, leg. G.C. Addison-Williamson.

**Mauritius** — NHMUK Entomology: 2000-110, two spms., Raphael island, St. Brandon, Mauritius, January 1996.

**Seychelles** — NHMUK, one spm., with label “No. 13216?”, Praslin, January 1953, det. E.S. Brown. NHMUK 1952.12.17.248-249, two spms., Silhouette Island, leg. J.S. Gardiner. NHMUK, one spm., Mahe, leg. J.M and R.D. Pope, August 1976, det. MacFarlane. NHMUK 1867.76, two spms., Seychelles, leg. A. Newton Esq. NHMW Inv. No. 8597, two spms., Mahe, leg. Brauer, det. Attems C.

**Comoros** — NHMUK Entomology: 2000-110, one spm., Comores, leg. Helen Read, May 1995.

**Pacific Islands** — NHMUK, one spm., South Pacific Islands, leg. J.M. Selfridge Mhl., 2/3/1885. NHMUK 1950.4.19.13, one spm., Nukualofa, Tonga, 22/2/1925, det. Brolemann. NHMUK 1950.4.19.6 and 11, two spms., Apia, Upolu, Samoa, leg. Buxton and Hopkins, 7/5/1924. NHMUK, one spm., Ahui, Tautira, Tahiti, 9/8/1925, leg. Cheesman. NHMUK, one spm., Pahenoo, Tahiti, South Pacific, 3/1925, leg. Col. S.Y. Sr. George P.H. Johnson S.Z.R.Q. NHMUK 1882.60, one spm., Ravatonga, leg. Sir J. Fulbock. NHMUK 1911.12.4.14, one spm., Savau, Friendly Island. NHMUK, one spm., Hawaiian Islands, leg. Henry Edward, 30/12/1875. NHMUK 1882.60, one spm., Rarotonga, Sir J. Lubbock’s collection. NHMUK 1926.1.24.465, one spm., on mango trunk, Fatu Hiva, Marquesas Island, leg. P.H. Johnson S.Z.R.A, 6/1/1925. NHMUK 1926.1.24.466-470, five spms., Hiva Oa, Marquesas Island, January 1924, leg. P.H. Johnson.

**United Kingdom** — NHMUK, one spm., in flat over banana-ripening store, Poplar, London, leg. E.Z.H.O.H., 9/10/1956. NHMUK 1894.12.23.1, one spm., Kew Gardens. NHMUK, one spm., imported with bananas, Windsor, 14/6/1965, det. P.C. Jerrard, 1965. NHMUK, one spm., West Indies dock, London. NHMUK, one spm., imported with bananas, Boston, Lincs [Lincolnshire, U.K.], det. P.C. Jerrard, 24/10/1960.

**Caribbean Sea** — NHMUK, two spms., Barbados, det. J. Locke Esq. NHMUK 1886.113-116, one spm., Montserrat. NHMUK 1899.6.1-3, three adults, numerous juvenile spms., St. John (West Indies), leg. J.W. Gregory. NHMUK, one spm., leg. Morne Fortune, R.F.S., det. D.J. Clark. NHMUK, one spm., Bermuda, April, 1873. NHMUK 1896.3.17.21.22, two spms., Antigua, leg. W.R. Forrest. NHMUK, one spm., Bermuda, April, 1873.

**Central and South America** — NHMUK 1913.6.13.398-399, two spms., Bogotia [Bogotá, Colombia]. NHMUK 1898.2.12.15, one spm., Rio Jurua, Amazons, leg. Dr. Bach. NHMUK 1913.6.18.900, one spm., Cayenne. NHMUK, one spm., Rio Sofiars, northwest Ecuador, 450 ft., leg. Rosenberg. NHMUK 1905.7.13.1, one spm., Isthmus of Panama, leg. Mr. H. Robert.

**Undetermined locality** — NHMUK, one spm., unknown locality, with label “No.47/21”. NHMUK 1813.6.18.201-202, one spm., with label “*Scolopendra* Div.I seet. B.6 Cintillus”. NHMUK 1813.6.18.904, one spm., with label “*Scolopendra subspinipes* I (typical form)”. NHMUK 1916.10.4.4-8, one spm., unknown locality. NHMUK, one spm., Rei Islands, leg. Cahl. Lingen, with label “88-100”. NHMW Inv. No. 703, one spm., Ostindien [East Indies].

**Specimens referred to *Scolopendra mutilans* Koch, 1878**: **Japan** — **Syntype** NHMW Inv. No. 751 of *Scolopendra mutilans* Koch, 1878, one spm., Japan, with label “Syntype”, leg. Roletz, don. Latzel, 1919 (Figs 12–13). NHMUK 1911.12.12.915-916, two spms., Izu, Japan, leg. S. Akiyama. NHMUK, one spm., Yokohama, Japan, HMS Challenger Expedition, May 1875. NHMUK, two spms., Kole [Kobe], Japan, June 1875. NHMW Inv. No. 746, one spm., Nagasaki, Japan, leg. Rausonel, 1871. NHMW Inv. No. 738, seven spms., Japan, don. Roretz, 1/3/1881. NHMW Inv. No. 740, six spms., Japan.

**China** — NHMUK 1886.120, one spm., Snowy valley, Ningbo. NHMUK 1892.12.6.1, one spm., Chusan Island [Zhoushan], leg. J.J. Walkes.

**Korea** — NHMUK 1882.14, two spms., Southeast Korea. NHMUK, two spms., Kang-hwa [Khangwhado], Korea, leg. Miss Scarlett.

**Undetermined locality** — NHMUK 1888.50, one spm., Seimer Island (Pabva), leg. H.O.F. NHMUK, two spms., Tsur Island, leg. Holst, July-August 1891. NHMW, nine spms., unknown locality determined as “*Scolopendra mutilans*”.

##### Diagnosis.

17–19 antennal articles, 6 basal articles glabrous dorsally. Each tooth-plate with (4)5-7 teeth. Tergites 3(4)-20 with paramedian sutures. Complete tergite margination on TT14 (17)-21. Tergite of ultimate leg-bearing segment without depression or median suture. Complete paramedian sutures on sternites 2(3)-20. Coxopleural process with 2 apical spines, without lateral and dorsal spine. Ultimate leg prefemora with 2 VL, 1–2 M, 0–3 DM and prefemoral process with 1–6 spines. One tarsal spur on legs 1–19 or 20.

##### Composite description.

Body length up to 16 cm. Reddish brown colouration on entire body. Cephalic plate and segments monochromatic or dichromatic. Tergites reddish brown; dark band on posterior border of tergites. Cephalic plate with small punctae on anterior part; median sulcus present. Posterior part of cephalic plate without paramedian sulci.

Antenna usually with 18–19 articles (16–17 articles on one side in some specimens), basal 6 subcylindrical and glabrous dorsally (Fig. 16A), 6 articles glabrous ventrally. Antennae reach segment 3–4. Forcipular trochanteroprefemoral process with denticles in two groups, 1–3 apical and 1–2 inner (Fig. 16B, D). Tooth-plates wider than long or nearly as long as wide, 5–7 teeth (Figs 10D, 16B, D); atypically with 10 on one side (NHMUK specimen from western New Guinea). Tooth-plate with straight, transverse basal suture. Coxosternite without median suture (Figs 10C, 16D). Article 2 of second maxillary telopodite with spur.

**Figure 9.**
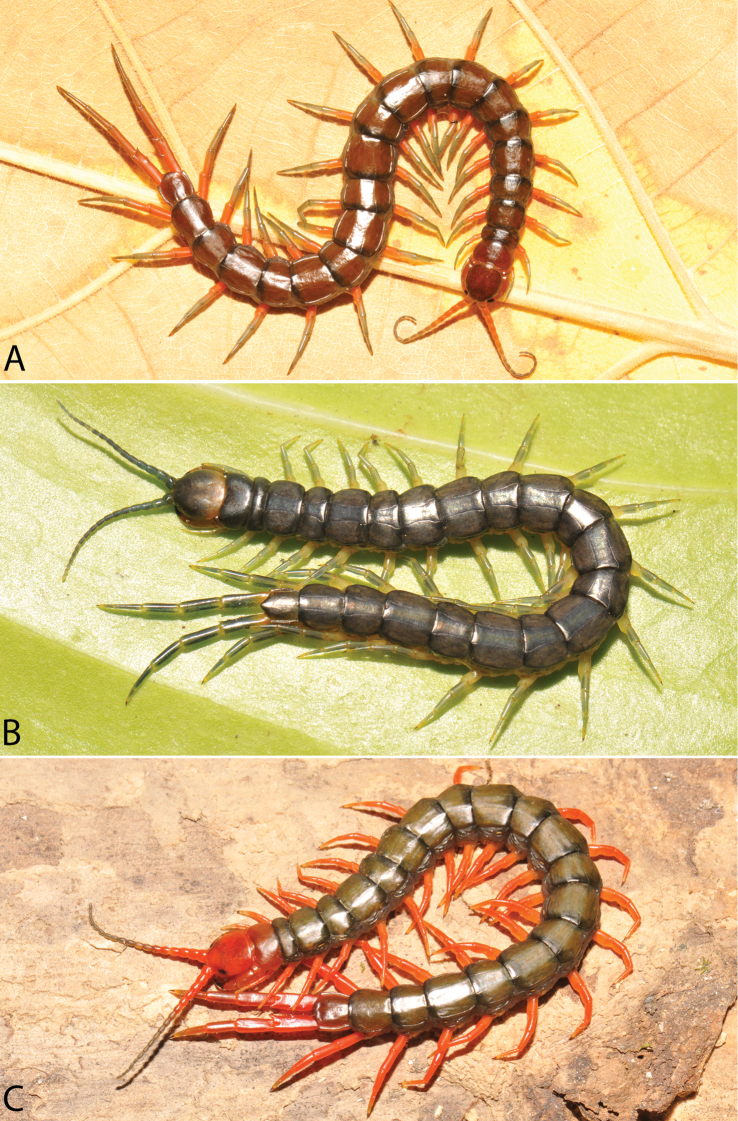
Habitus photographs of *Scolopendra* species: **A** *Scolopendra subspinipes* (Singapore, CUMZ 00315) **B** *Scolopendra calcarata* (Thailand, CUMZ 00418) **C** *Scolopendra japonica* (Colour morph 2: Laos, CUMZ 00298).

**Figure 10.**
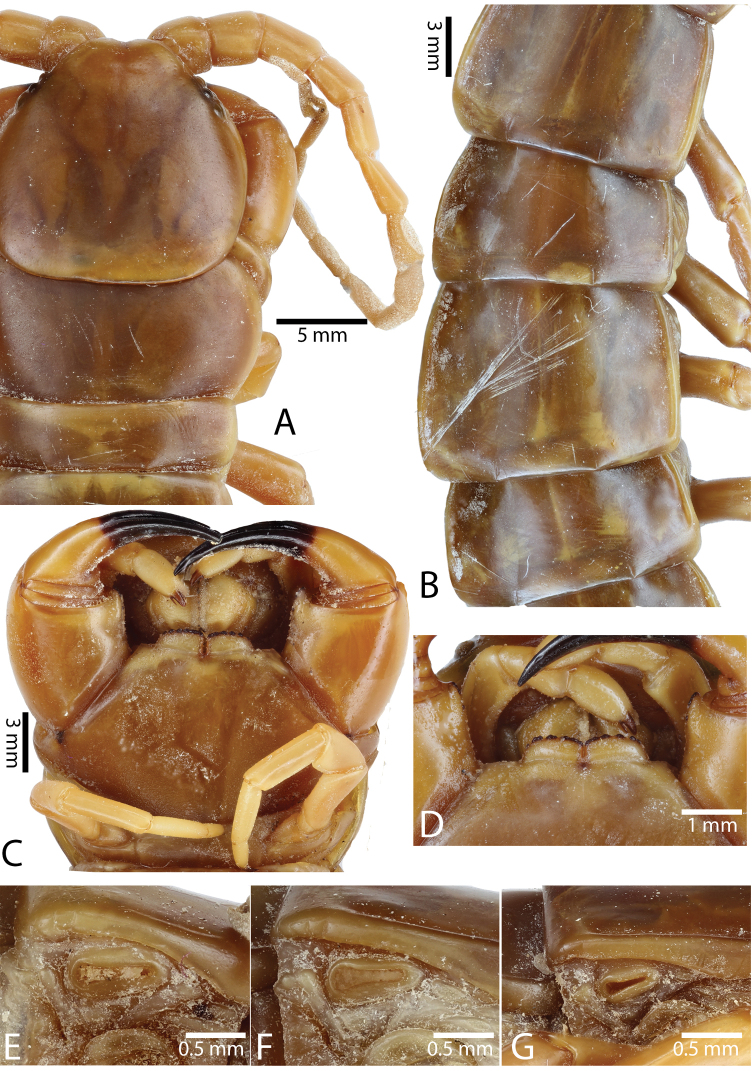
*Scolopendra subspinipes* (NHMUK): **A** Cephalic plate and trunk segments 1–2 **B** Tergites 9–12 **C** Forcipular segment **D** Tooth-plates **E**–**G** Spiracles 3, 5 and 8, respectively.

**Figure 11.**
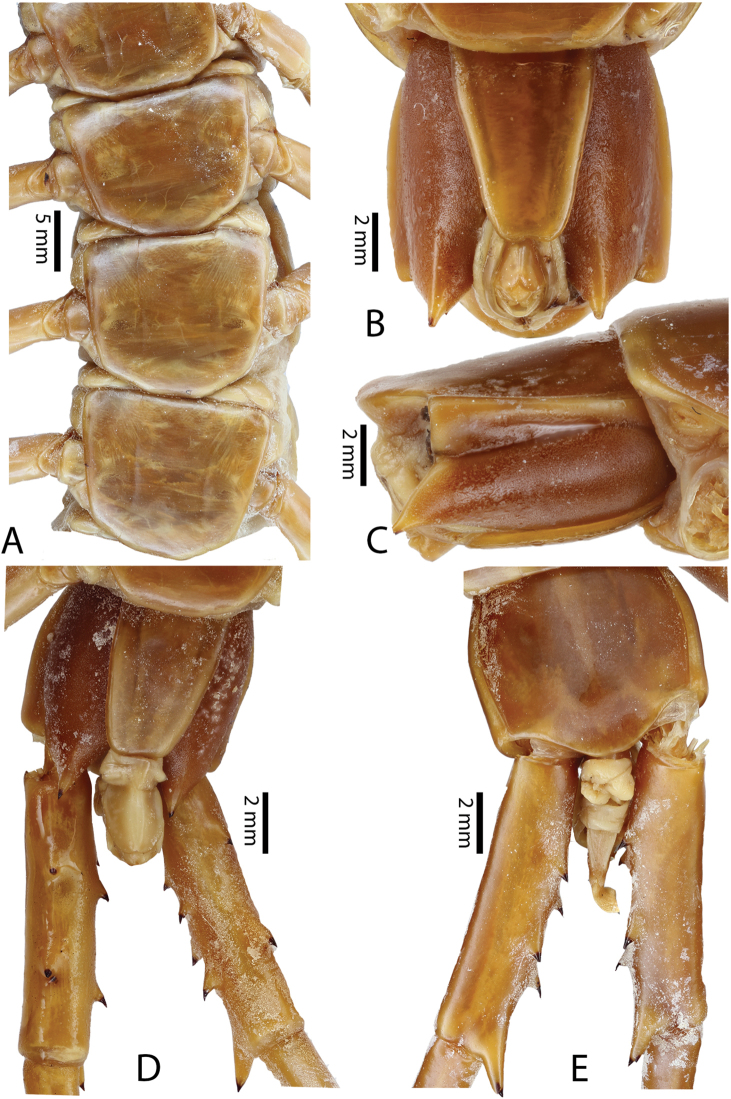
*Scolopendra subspinipes* (NHMUK): **A** Sternites 8–11 **B** Sternite of ultimate leg-bearing segment and coxopleura **C** Lateral view of coxopleuron **D** Ventral view of ultimate leg-bearing segment and ultimate leg prefemora **E** Dorsal view of ultimate leg-bearing segment and prefemora.

Anterior margin of T1 underlying cephalic plate (Figs 10A, 16A). Complete paramedian sutures on TT3–4; margination typically starting on TT5–10 (atypically from TT12–13 in some specimens). Tergite surface (Figs 10B, 16C) smooth. Tergite of ultimate leg-bearing segment (Fig. 17C) curved posteriorly, without median suture or depression; ratio of width: length of tergite of ultimate leg-bearing segment 0.8:1. Sternites (Figs 11A, 17A) with complete paramedian sutures. Surface of sternites smooth, without depression. Sternite of ultimate leg-bearing segment (Fig. 11B) with sides converging posteriorly; surface without depression. Pore-field on coxopleuron terminating far beneath margin of tergite of ultimate leg-bearing segment, dorsal margin of pore area gently sinuous (Figs 11C, 17B).

Coxopleural process moderately long, with two apical and 0–1 subapical spines; pore-free area extending 30–70% length from distal part of coxopleural process to margin of sternite of ultimate leg-bearing segment (Figs 11B, 17D).

All legs without setae and tibial spur. One tarsal spur on legs 1–19 or more commonly 1–20. Ultimate legs: moderately long and slender, with ratios of lengths of prefemur and femur 1.4:1, femur and tibia 1.2:1, tibia and tarsus 2 1.4:1.; tarsus 1 and tarsus 2 2:1. Prefemoral spines: 2 VL, 1–2 M, 0–3 DM and prefemoral process with 1–6 spines (Figs 11D–E, 17D–E). Posterior margin of prefemur with short median groove.

Genital segments well developed, reaching longer than distance between posterior margin of sternite of ultimate leg-bearing segment and distal part of coxopleural process. Sternite of genital segment 1 round and convex posteriorly, with median suture. In male, sternite of genital segment 2 attached to penis. Tergite of genital segment without small setae. Gonopods with small setae in male. Penis with apical bristle.

##### Discussion.

**Figure 12.**
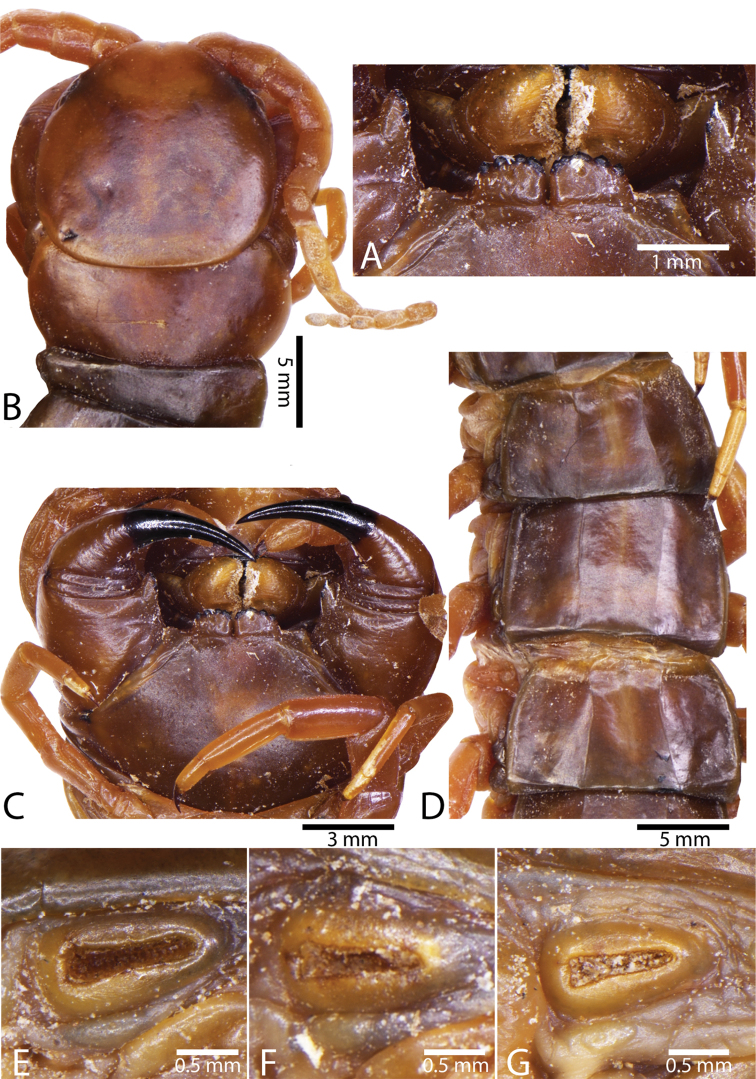
*Scolopendra subspinipes* (Syntype NHMW 751 of *Scolopendra mutilans* Koch, 1878): **A** Tooth-plates **B** Cephalic plate and trunk segments 1–2 **C** Forcipular segment **D** Tergites 9–11 **E–G** Spiracles 3, 5 and 8, respectively.

**Figure 13.**
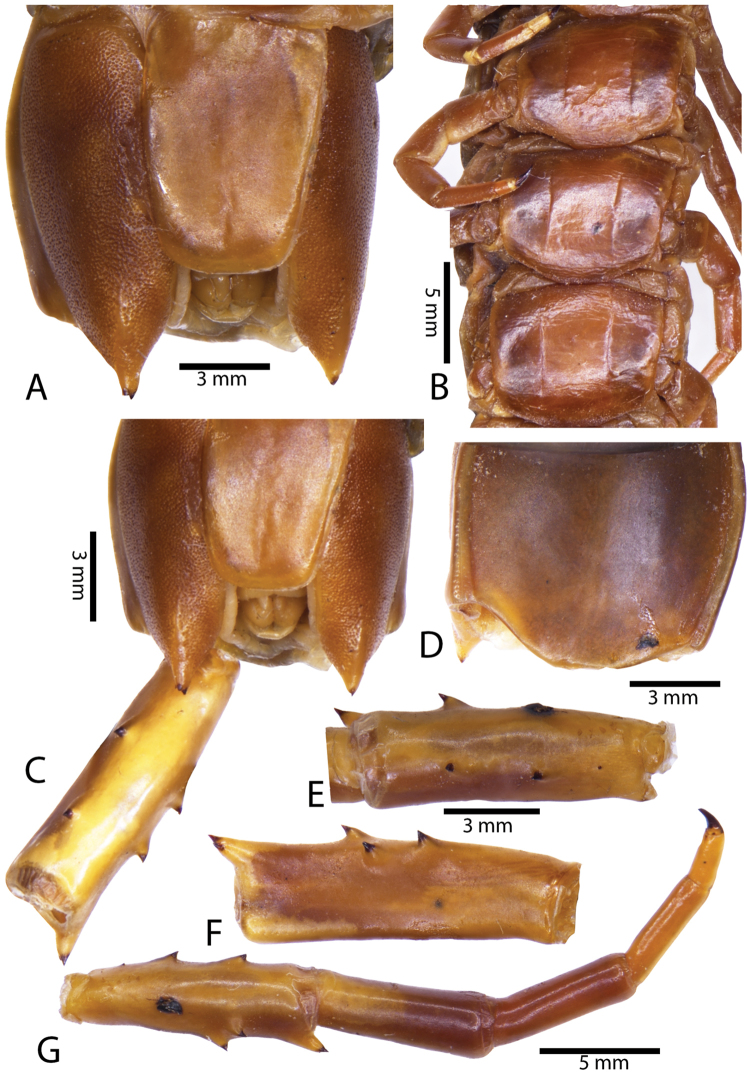
*Scolopendra subspinipes* (Syntype NHMW 751 of *Scolopendra mutilans* Koch, 1878): **A** Sternite of ultimate leg-bearing segment and coxopleura **B** Sternites 10–12 **C** Sternite of ultimate leg-bearing segment, coxopleura and ventral view of ultimate leg prefemur **D** Tergite of ultimate leg-bearing segment **E** Ventral view of ultimate leg prefemur **F** Dorsal view of ultimate leg prefemur **G** Ventro-lateral view of ultimate leg.

Recently, the taxonomic validity of *Scolopendra subspinipes* and its former subspecies has been evaluated both by morphology (Kronmüller 2012) and molecular methods (Chao et al. 2011, Siriwut et al. 2015a). Three former subspecies of *Scolopendra subspinipes*, namely *Scolopendra subspinipes japonica*, *Scolopendra subspinipes dehaani* and *Scolopendra subspinipes cingulatoides* (= *Scolopendra dawydoffi*), have been raised to species rank (Kronmüller 2012), whereas the remaining four subspecies (in the classification of Attems (1930b)) have been synonymized with the nominotypical subspecies. However, some subspecies still remain of ambiguous status. Notably, *Scolopendra subspinipes mutilans* Koch, 1878, a nominal subspecies occurring in East Asia, corresponds to *Scolopendra subspinipes* in all respects apart from the cephalic plate and T1 showing reddish colouration. Recent morphological revisions have regarded this subspecies to be a synonym of *Scolopendra subspinipes* (Schileyko 2007, Kronmüller 2012), whereas molecular analyses based on four loci found it to either resolve as sister taxon to *Scolopendra subspinipes* s.str. or to group more closely with other species (Vahtera et al. 2013). In this study, we document a syntype of *Scolopendra mutilans* Koch, 1878 in the NHMW collection (Figs 12–13) and reconfirmed its taxonomic status by using molecular analysis from the concatenated DNA dataset of *Scolopendra subspinipes* s.str. and *Scolopendra mutilans* Koch, 1878. The phylogenetic tree supports the proposition that this subspecies cannot be distinguished taxonomically from *Scolopendra subspinipes*. According to genetic divergence among examined populations, *Scolopendra mutilans* Koch, 1878 should be regarded as a geographical variant of *Scolopendra subspinipes*, as was suggested in other recent taxonomic studies (Schileyko 1995, 2007). Some morphological comparisons of several populations from Southeast and East Asia are provided in Table 6.

**Table 6.** Morphological comparison of *Scolopendra subspinipes* populations from different geographical regions. ? insufficient data.

Character	Bay of Bengal and Indian Ocean^1^	Malay Peninsula^1^	Vietnam ^2^	Indonesia^1^	Philippines^1^	Taiwan ^3^	China^1^	Japan^1^
Number of antennal articles	18	18–19	18–19	17–19	17–18	18–19	16–18	15–19
Number of glabrous articles	6	6	6	6	6	6	6	6
Teeth on tooth-plate	5+5	4+6, 5+5, 6+5, 6+6, 7+7	4–9 (each side?)	5+5, 6+7, 7+7, 6+10	5+5, 10+5, 7+7	5+5	5+5	4+5, 5+5, 6+5, 6+6
First tergite with complete paramedian sutures	3	3	2(9) (poorly defined in some specimens)	3–4	3–4	4–6	3–6	3–6
First tergite with margination	5	4–10	14–15	4–11	3–5	5–9	5–8	5–16
Tergite surface	smooth	smooth	smooth	smooth	smooth	smooth	smooth	smooth
Median furrow on tergite of ULBS	absent	absent	absent	absent	absent	absent	absent	absent
Paramedian sutures on sternites	complete	complete	complete	complete	complete	incomplete	complete	complete
Sternite of ultimate leg-bearing segment	without pit	pit-like median furrow	?	pit-like median furrow	without pit	?	pit-like median furrow	pit-like median furrow
Spines on coxopleural process	AP: 2	AP: 2 SAP: 0–1	AP: 2	AP: 2 SAP: 0–1	AP: 2 SAP: 0–1	AP: 2–3	AP: 1–2 SAP: 0–1	AP: 0–2 SAP: 0–1
Spine formula on prefemora of ultimate legs	VL: 2 M: 1 DM: 1 SP: 2	VL: 2–3 M: 1–2 DM: 1–2 SP: 2–3	VL: 0–3 M: 0–2 DM: 0–3 SP: 1≥	VL: 2 M: 1–2 DM: 1–2 SP: 2–5	VL: 2 M: 1–2 DM: 1–2 SP: 2	VL: 2 VM: 1 DM: 2 SP: 2–4	VL: 2–3 M: 1 DM: 1 SP: 1–2	VL: 0–3 M: 0–2 DM: 0–2 SP: 1–6
Legs with one tarsal spur	1–20(R)	1–19(20)	1–19	1–19 (20)	1–19 (20)	1–20	1–20	1–19(20)

**Note**: each superscript number refers to description in previous and present studies as follow: ^1^ = this study, ^2^ = Schileyko (1995), ^3^ = Chao (2008).

*Scolopendra subspinipes piceoflava*, another former subspecies of *Scolopendra subspinipes* from Sulawesi, Indonesia, is currently treated as a synonym of *Scolopendra subspinipes* (see Kronmüller 2012), but may upon further study prove to be a valid species. Attems (1934) stated that it could be distinguished from other forms of *Scolopendra subspinipes* by yellowish colouration on the posterior part of its tergites. He also referred to the weakness or near absence of tergal paramedian sutures, which also occurs in some other SE Asian *Scolopendra* species (see Kronmüller 2012 and discussion of *Scolopendra cataracta* in this study). Re-examining the syntypes of *Scolopendra subspinipes piceoflava* leads us to dispute the taxonomic validity of this character because paramedian sutures are visible on the tergites in all syntypes. In order to provide a more complete evaluation of its taxonomic status, a redescription of its syntypes is as follows:

#### Scolopendra subspinipes piceoflava

Taxon classificationAnimaliaScolopendromorphaScolopendridae

Attems, 1934

Figs 14 , 15

##### Material.

**Figure 14.**
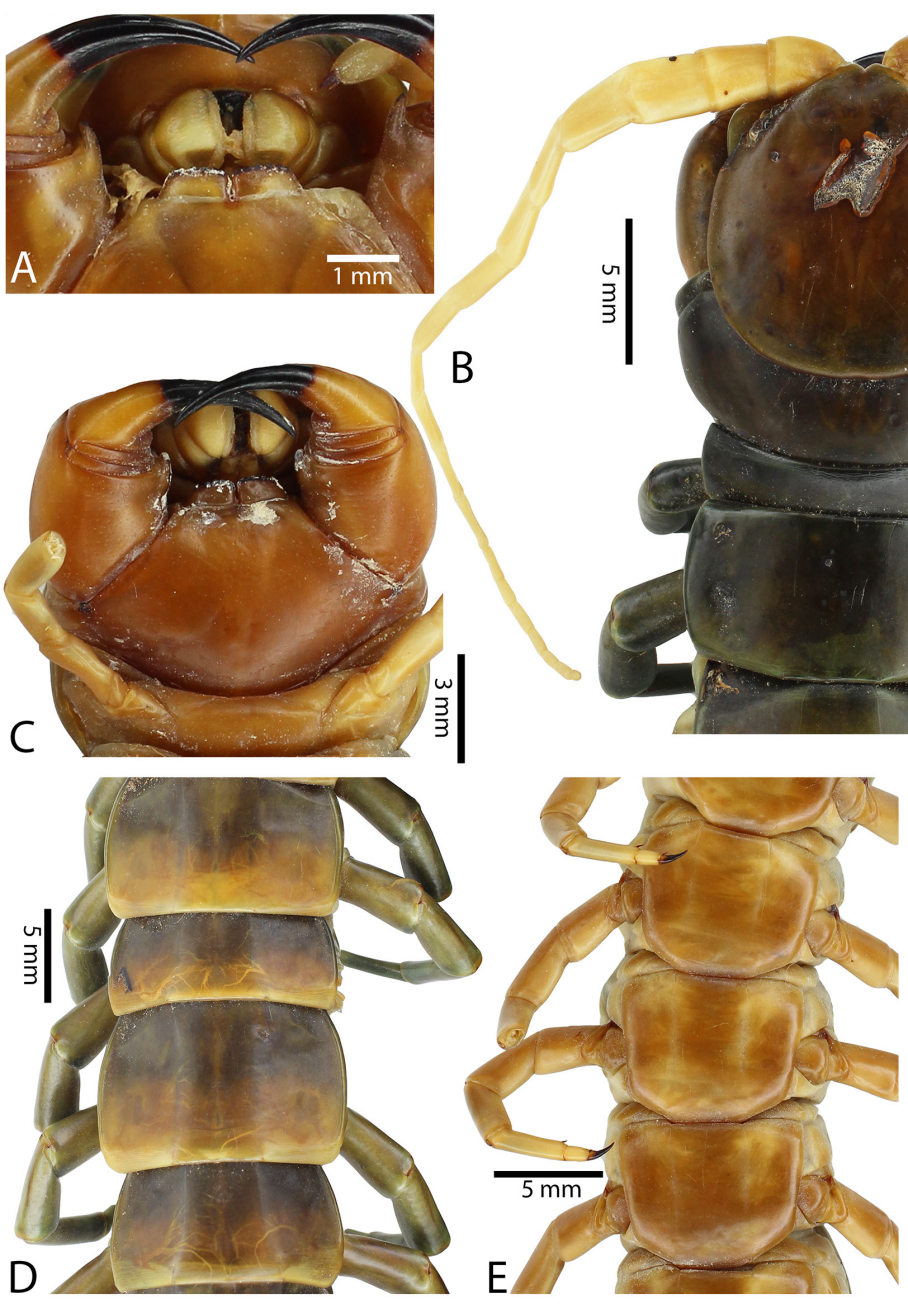
*Scolopendra subspinipes* (Syntypes NMB 391Va of “*piceoflava* Attems, 1934”): **A** Tooth-plates **B** Cephalic plate and trunk segments 1–3 **C** Forcipular segment **D** Tergites 9–12 **E** Sternites 8–10.

**Figure 15.**
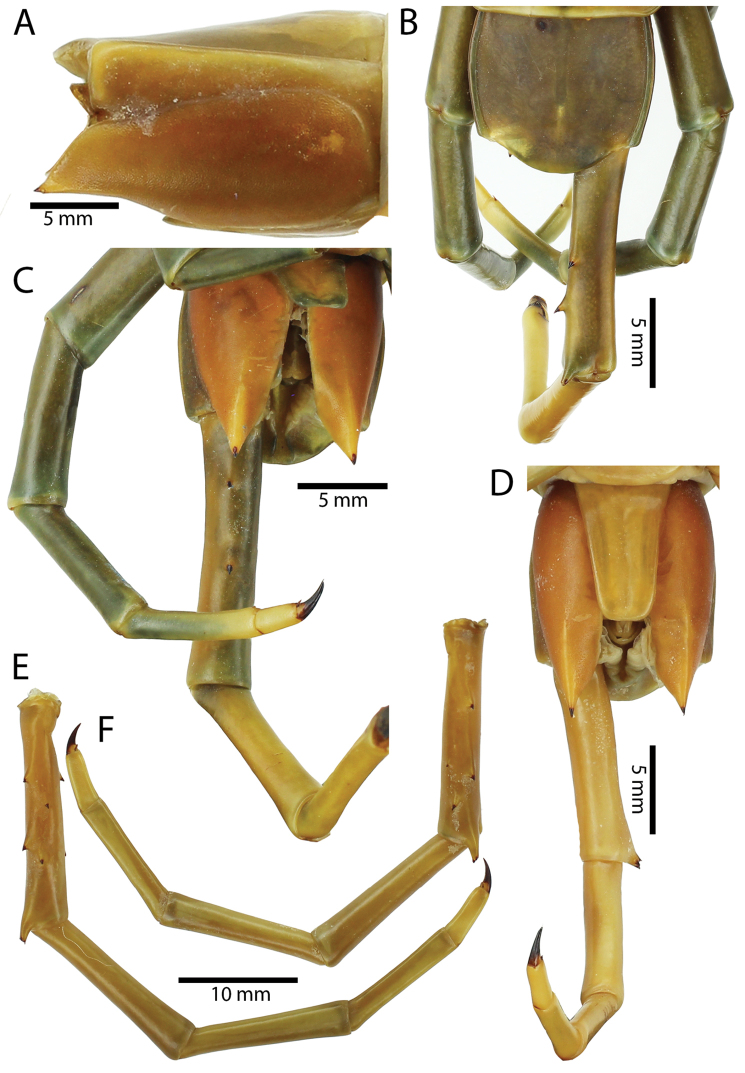
*Scolopendra subspinipes* (Syntypes NMB 391Va of “*piceoflava* Attems, 1934”): **A** Lateral view of coxopleuron **B** Tergite of ultimate leg-bearing segment and dorsal view of legs 20 and ultimate leg **C** Ventral view of Leg 20, coxopleura and ultimate leg **D** Sternite of ultimate leg-bearing segment, coxopleura and ultimate leg **E–F** Ventro-lateral view of ultimate legs.

**Syntypes** NMB 391Va spec.1–3, one adult male and two adult females, Central Celebes, don. Z.U.F. Sarasin, 1895 (Figs 14, 15).

**Figure 16.**
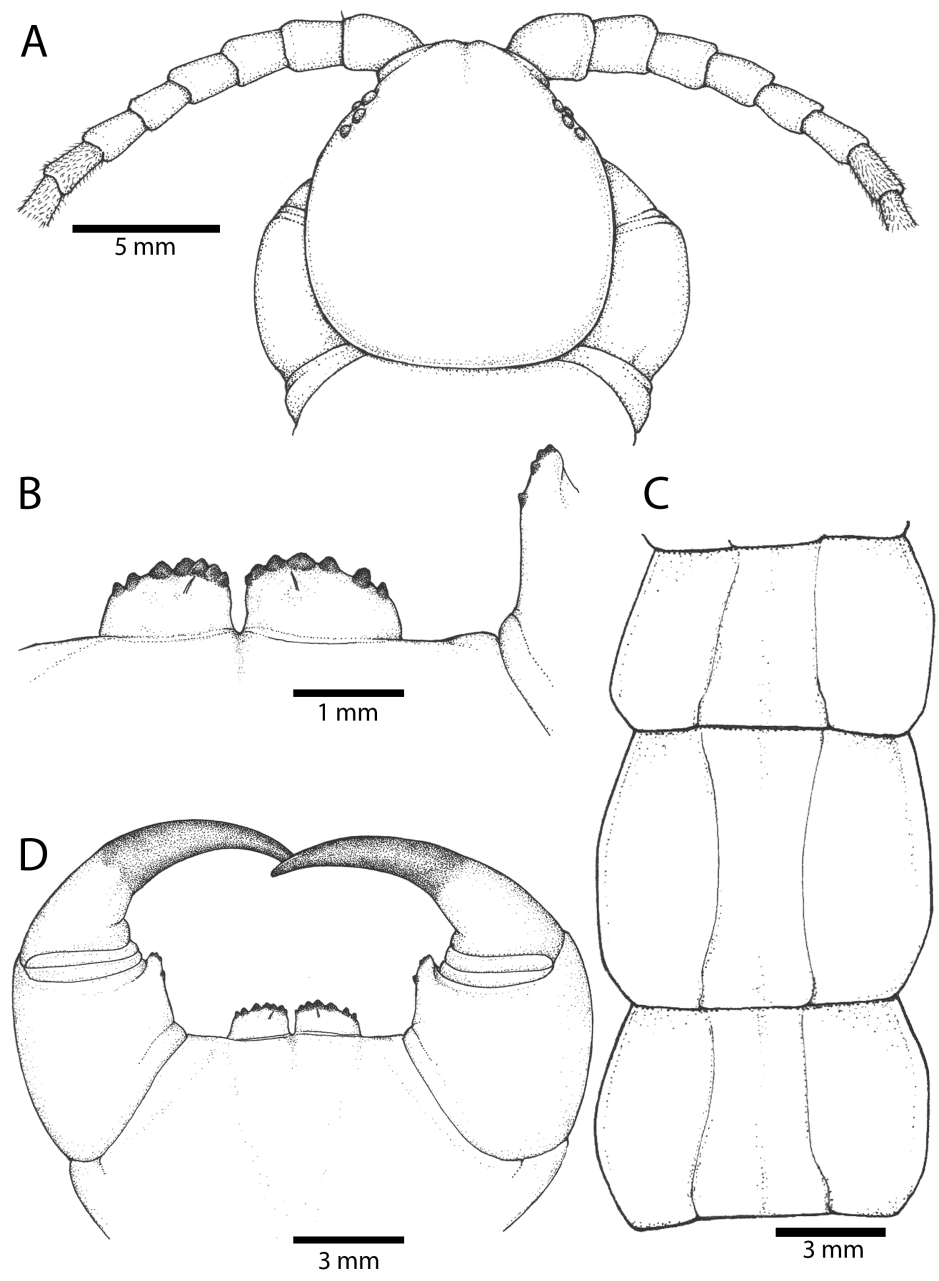
*Scolopendra subspinipes* (CUMZ 00315): **A** Cephalic plate and basal antennal articles **B** Tooth-plates and trochanteroprefemoral process **C** Tergites 9–11 **D** Forcipular segment.

**Figure 17.**
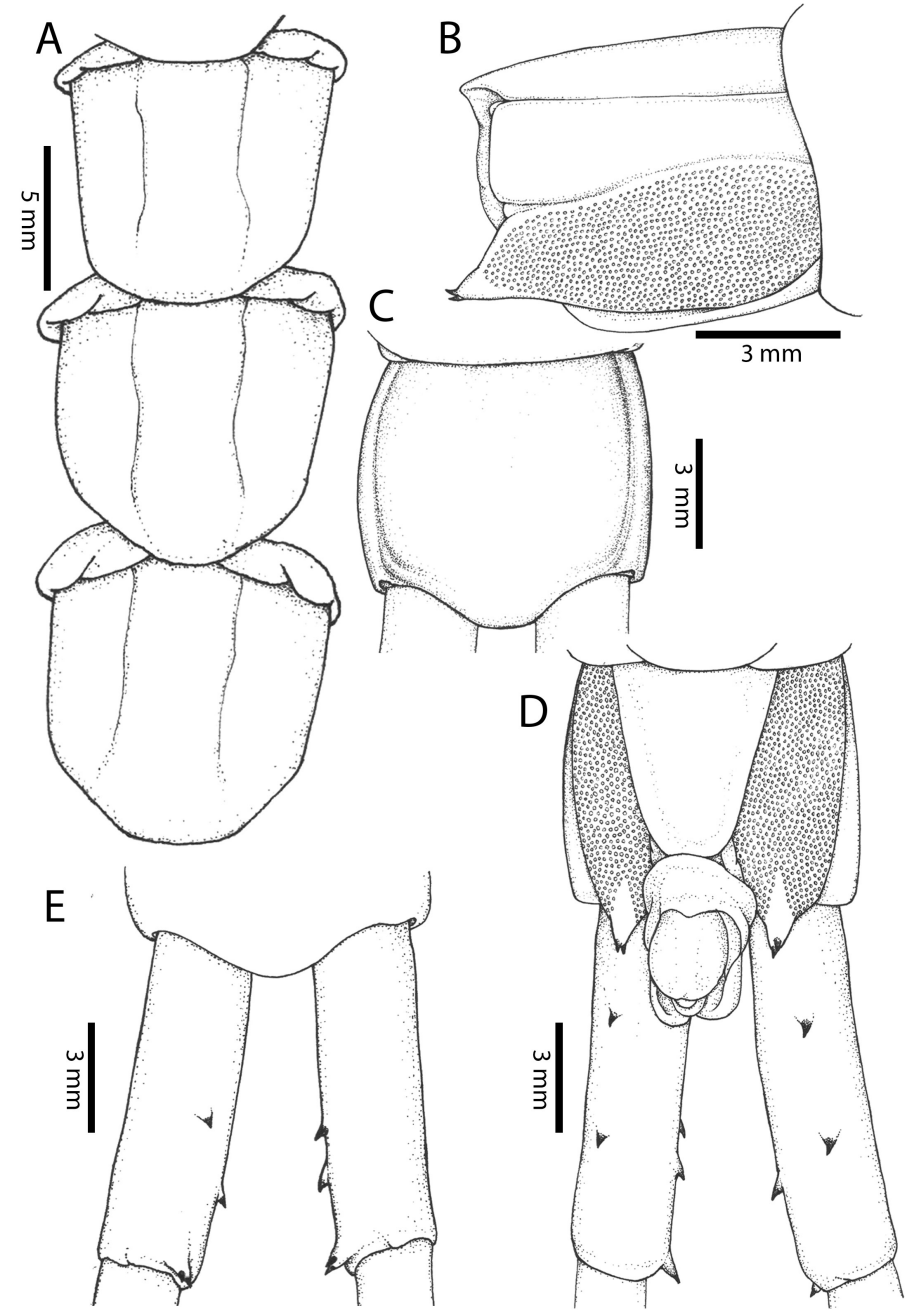
*Scolopendra subspinipes* (CUMZ 00315): **A** Sternites 9–11 **B** Lateral view of coxopleuron **C** Tergite of ultimate leg-bearing segment **D** Sternite of ultimate leg-bearing segment, coxopleura, female genital segment and ultimate leg prefemora **E** Dorsal view of ultimate leg prefemora.

##### Type locality.

Tomohon, Sulawesi, Indonesia [Tomohon, North Sulawesi Province, Indonesia].

##### Description.

Body length 16.7 cm in male and 17.1 and 16.5 cm in female syntypes. Preserved male still exhibiting traces of its colouration pattern: cephalic plate and segments dark greenish or brown. Antenna yellowish. Tergites with yellowish or pale colour on posterior margin. All legs blue-greenish, distal part yellow. Cephalic plate without small punctae on anterior part, median sulcus present. Posterior part of cephalic plate without paramedian sulci.

Antenna usually with 17–19 articles, basal 6 subcylindrical and glabrous both on dorsal and ventral sides. Antennae reach segment 4 (Fig. 14B). Forcipular trochanteroprefemoral process bearing denticles in two groups, 2–3 apical and one inner. Tooth-plates wider than long or nearly equivalent, 6–7 teeth (Fig. 14A). Tooth-plate with straight, transverse basal suture. Coxosternite smooth without median suture, with shallow depression in male specimen (Fig. 14C). Article 2 of second maxillary telopodite with spur.

Anterior margin of T1 underlying cephalic plate (Fig. 14B). Complete paramedian sutures from T4; margination typically starting on TT5–7. Tergite surface (Fig. 14D) smooth, without median sulci. Tergite of ultimate leg-bearing segment (Fig. 15B) curved posteriorly, without median furrow or depression; ratio of width: length of tergite of ultimate leg-bearing segment 0.82:1. Sternites (Fig. 14E) with incomplete paramedian sutures, extending 80% length of sternite on anterior segments. Surface of sternites smooth, without depression. Sternite of ultimate leg-bearing segment (Fig. 15D) with sides converging posteriorly; surface typically without depression (with median depression in one female specimen; NMB391Va sp.1). Pore-field on coxopleuron well developed, with gently curved dorsal margin, reaching nearly to margin of tergite of ultimate leg-bearing segment, anterior part of pore area widest (Fig. 15A).

Coxopleural process long (Fig. 15C–D) with 1–2 apical spine(s) and absence of lateral and dorsal spines; pore-free area extending 30–50% length from distal part of coxopleural process to margin of sternite of ultimate leg-bearing segment.

All legs without setae and tibial spur. One tarsal spur on legs 1–20. Ultimate legs: slender and long (Fig. 15E–F), with ratios of lengths of prefemur and femur 1.2:1, femur and tibia 1.1:1, tibia and tarsus 2 1.3:1, tarsus 1 and tarsus 2 2.3:1. Prefemoral spines: 0–2 VL, 1–2 M, 1–2 DM and prefemoral process with 2–5 spines. Posterior margin of prefemur with acute median groove.

Sternite of genital segment 1 round and convex posteriorly, with median suture. In male, sternite of genital segment 2 attached to penis. Tergite of genital segment without small setae. Gonopods present in male.

##### Discussion.

Based on examination of the syntypes, we corroborate the assignment of this nominal subspecies to the *Scolopendra subspinipes* group. Some morphological characters that appear, however, not to be identical with *Scolopendra subspinipes* are the sharpness and length of the coxopleural process, which bears one or two strong apical spines, the ratio of ultimate leg podomeres, and the colouration pattern on the tergites that is clearly distinct from other geographical populations of *Scolopendra subspinipes* (the posterior part of the tergites exhibiting a yellowish colouration). On the other hand, the syntypes of *Scolopendra subspinipes piceoflava* also display morphological variation between each other with respect to the number of prefemoral spines on the ultimate legs: a male specimen has 4–6 spines on the prefemoral process whereas VL, M and DM spines are absent in one female specimen. The latter is similar to *Scolopendra dehaani* but it is possible that this absence may be due to regeneration in this individual. However, without additional material and lacking molecular data with which to test relationships among morphological similar species, we tentatively accept *Scolopendra subspinipes piceoflava* as a junior synonym of *Scolopendra subspinipes* as proposed by Kronmüller (2012).

##### Distribution.

**Figure 18.**
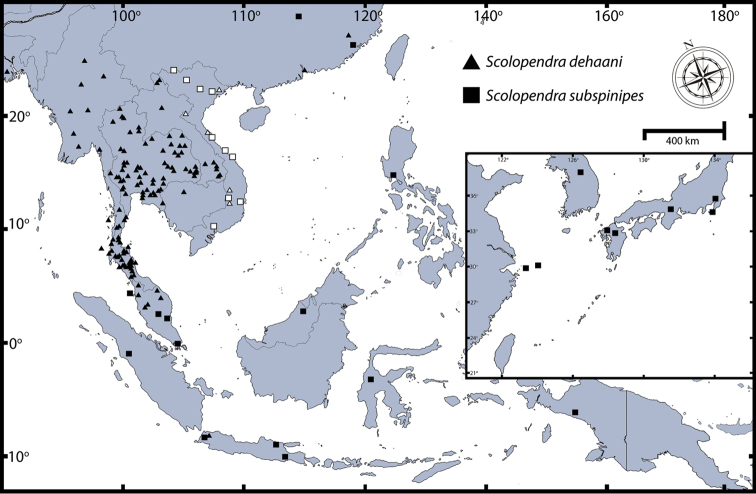
Distribution map of *Scolopendra dehaani* and *Scolopendra subspinipes* in Southeast Asia and China-Japan Sea (small map): Filled and blank colours refer to localities from the present study and in the literature, respectively.

Previous studies regarded *Scolopendra subspinipes* s.l. as a cosmopolitan species in tropical regions (Schileyko 2007; Chao 2008; Lewis 2010b). In this study, most of the sampled specimens were collected on islands. Several old collections in the NHMUK identified as *Scolopendra subspinipes* sensu lato from mainland East and Southeast Asia instead refer to former subspecies of *Scolopendra subspinipes* that are now identified as distinct species, including *Scolopendra dawydoffi*, *Scolopendra dehaani*, *Scolopendra multidens* and *Scolopendra japonica*. For this reason, we removed occurrence records of *Scolopendra subspinipes* s.l. from Thailand and Laos due to our extensive surveys throughout these two countries, finding that no specimen of *Scolopendra subspinipes* s.str. was found in this area. The updated distribution of this species in Asia (Fig. 18) is as follows: **Southeast Asia**: Myanmar, Malaysia (Penang and Sarawak), Singapore, Vietnam (fide Schileyko 2007: Lao Cai, Vinh Phuc, HatTay, Hai Phong, Quang Binh, Thua Thien Hue, Da Nang, Dak Lak, Khanh Hoa and Dong Nai Provinces), Indonesia (Surabaya, Java, east coast of Sumatra, Lombok, Sumbawa and Mimika River, New Guinea) and Philippines (Manilla). **East Asia**: China (Zhouzhan Island, Ningbo and Changsha), Taiwan (Kang-hwa), Japan (Izu and Goto Islands, Yokohama and Kobe) and South Korea.

#### Scolopendra dehaani

Taxon classificationAnimaliaScolopendromorphaScolopendridae

Brandt, 1840

Figs 7A , 18 , 19 , 20 , 21 , 22 , 23 , 24 and 25A

Scolopendra dehaani Brandt, 1840: 152, 1841: 59. Newport 1844: 96, 1845: 394. Kohlrausch 1881: 47. Meinert 1886: 203. Flower 1901: 21. Siriwut et al. 2015a: 7, table 1, figs 1–2. Kronmüller 2012: 24, figs 3C, 4D, table 1.
Scolopendra subspinipes dehaani — Pocock 1891: 409, 1894: 312. Silvestri 1895: 714. Kraepelin 1903: 260. Attems 1907: 80, 1914a: 106, 1930b: 31, 1938: 334, 1953: 138. Takakuwa 1942c: 41. Wang 1955b: 16, 1956: 158, 1965b: 449. Schileyko 1998: 268, 2007: 75. Chao and Chang 2003: 2. Lewis 2010b: 113. Tran et al. 2013: 230.
Scolopendra subspinipes — Jangi and Dass 1984: 30, figs 2–6.
Scolopendra childreni Newport, 1844: 96. Kraepelin 1903: 260.
Scolopendra concolor Newport, 1845: 394. Daday 1889: 150, 1891: 188. Kraepelin 1903: 260.
Scolopendra inermis Newport, 1845: 393. Koch 1863: 64, pl. 29, fig. 55.
Scolopendra inermipes Koch, 1847: 153. Kraepelin 1903: 260.
Scolopendra silhetensis Newport, 1845: 393. Kraepelin 1903: 260.
Scolopendra lucasii Gervais, 1847: 270. Kraepelin 1903: 260.
Scolopendra horrida Koch, 1847: 154. Kraepelin 1903: 260.
Scolopendra limicolor Wood, 1861: 12. Kraepelin 1903: 260.
Scolopendra bispinipes Wood, 1862: 28. Kraepelin 1903: 260.
Scolopendra fissispina Koch, 1865: 891. Kraepelin 1903: 260.
Scolopendra nudipes Tömösváry, 1885: 67. Kraepelin 1903: 260.
Scolopendra foveolata Verhoeff, 1937: 220. Würmli 1972: 91.
Scolopendra arborea Lewis, 1982: 389. Lewis 2010: 97. **Syn. nov.**

##### Type locality.

Java, Indonesia.

##### Material.

**Thailand** — CUMZ 00325, one spm., Pha Mon Cave, Pang Ma Pha, Mae Hong Son (19°30'01.6"N, 98°16'43.5"E). CUMZ 00324, one spm., Wat Ban Mai, Mae Hong Son (19°17'55.3"N, 97°59'13.5"E). CUMZ 00323, one spm., Wat Tham Chiangdao, Chiang Mai (19°23'36.8"N, 98°55'42.6"E). CUMZ 00377, one spm., Wat Tham Pak Piang, Chiang Dao, Chiang Mai (19°24'10.498"N, 98°55'52.691"E). CUMZ 00378, one spm., Tham Chiang Dao, Chiang Dao, Chiang Mai (19°23'35.758"N, 98°55'44.412"E). CUMZ 00379, five spms., Huai Hong Khrai, Doi Saket, Chiang Mai (18°51'26.107"N, 99°13'21.827"E). CUMZ 00346, one spm., Hui Hong Khrai, Chiang Mai (18°50'58.6"N, 99°13'18.9"E). CUMZ 00380, one spm., Suan Hin Maharat Stone Park, Long, Phrae (18°9'15.072"N, 99°59'10.343"E). CUMZ 00381, one spm., Lai Nan, Wieng Sa, Nan (18°36'39.969"N, 100°53'17.242"E). CUMZ 00382, two spms., Si Nan National Park, Na Noi, Nan (18°21'56.97"N, 100°49'56.393"E). CUMZ 00288, one spm., Phusang Waterfall, Phusang, Phayao (19°40'05.0"N, 100°23'25.1"E). CUMZ 00243, Hub Pa-Tat, Larnsak, Uthai Thani. CUMZ 00276, one spm., Wat Tham Erawan, Ban Rai, Uthai Thani (15°02'01.5"N, 99°27'16.6"E). CUMZ 00329, one spm., Wat Phothikhun, Maesot, Tak (16°44'39.2"N, 98°36'17.2"E). CUMZ 00374, two spms., Wat Tham Namphu Khao Rong Kwang, Lansak, Uthai Thani (15°25'59.212"N, 99°35'7.924"E). CUMZ 00375, two spms., Wat Tham Khao Chakkachan, Chum Ta Bong, Nakhon Sawan (15°35'48.533"N, 99°32'38.758"E). CUMZ 00376, one spm., Wat Khao Huai Lung, Banphot Phisai, Nakhon Sawan (15°55'41.844"N, 99°52'20.407"E). CUMZ 00257, one spm., Wat Huai Lung, Banphot Phisai, Nakhon Sawan (15°55'29.6"N, 99°52'28.0"E). CUMZ 00256, one spm., Bang Ban, Ayutthaya (14°21'51.4"N, 100°29'22.3"E). CUMZ 00282, one spm., Wang Kanlueang Waterfall, Chai Badan, Lopburi (15°06'49.4"N, 101°06'38.8"E). CUMZ 00289, one spm., Wat Khao Somphot, Chai Badan, Lopburi (15°09'42.2"N, 101°16'49.5"E). CUMZ 00286, one spm., Chaloem Phra Kiat, Saraburi (14°40'11.9"N, 100°53'09.4"E). CUMZ 00371, one spm., Chet Sao Noi Waterfall, Muak Lek District, Saraburi (14°43'35.463"N, 101°11'22.63"E). CUMZ 00372, one spm., Phu Khae Botanical Garden, Chaloem Phra Kiat, Saraburi (14°40'10.455"N, 100°53'13.401"E). CUMZ 00373, one spm., Wat Tham Welu Wan, Suphanburi (14°57'6.824"N, 99°38'57.176"E). CUMZ 00364, one spm., Wat Mai Luak, Sai Yok, Kanchanaburi (14°26'16.384"N, 98°52'19.822"E). CUMZ 00365, two spms., Wat Tha Thung Na, Sai Yok, Kanchanaburi (14°29'40.864"N, 98°50'21.332"E). CUMZ 00268, one spm., Wat Namtok, Saiyok, Kanchanaburi (14°13'47.5"N, 99°03'59.8"E). CUMZ 00270, 00361, two spms., Wat Tham Lijia, Sangkhaburi, Kanchanaburi (15°04'12.6"N, 98°33'59.0"E). CUMZ 00362, two spms., Wat Khao I-San, Ratchaburi (13°22'57.897"N, 99°46'21.374"E). CUMZ 00363, one spm., Central Botanical Garden, Ratchaburi (13°36'24.229"N, 99°39'37.584"E). CUMZ 00253, one spm., Tham Khao Bin, Ratchaburi (13°35'35.6"N, 99°40'02.3"E). CUMZ 00241, one spm., Wat Huailat, Phu Ruea, Loei, Thailand (17°27'00.4"N, 101°24'45.4"E). CUMZ 00383, one spm., Wat Pa Huai Lat, Phu Ruea, Loei (17°26'20.14"N, 101°25'17.223"E). CUMZ 00277, two spms. and CUMZ 00384, five spms., Tham Pha Pu, Loei (17°34'41.5"N, 101°42'39.1"E). CUMZ 00292, one spm., Wat Tham Pak Khaew Chiang Khan, Loei (17°52'34.0"N, 101°40'20.8"E). CUMZ 00247, one spm., Ban Dong Savanh, Phang Khon, Sakon Nakhon (17°28'17.9"N, 103°30'12.6"E). CUMZ 00266, one spm. and CUMZ 00392, one spm., Kham Hom Waterfall, Sakon Nakhorn (17°07'19.9"N, 104°01'07.4"E). CUMZ 00393, one spm., Wat Nikhom Kaset, Mukdahan (16°48'26.312"N, 104°42'41.205"E). CUMZ 00394, one spm., Pak Maenam Songkhram, Nakhon Phanom (17°38'23.43"N, 104°27'13.517"E). CUMZ 00395, one spm., Cha Naen Waterfall, Bueng Kan (18°12'54.298"N, 103°53'18.422"E). CUMZ 00385, one spm., Tham Pu Lup, Chum Phae, Khon Kaen (16°47'48.98"N, 102°37'31.026"E). CUMZ 00386, two spms., Wat Tham Phra, Tha Khantho, Kalasin (16°51'46.069"N, 103°15'57.202"E) CUMZ 00249, two spms., Ban Dan Chang, Tha Kantho, Kalasin (14°49'29.3"N, 99°41'36.5"E). CUMZ 00271, one spm., Wat Tham Phupha, Thakantho, Kalasin (16°48'05.7"N, 103°12'37.5"E). CUMZ 00275, two spms., Tha Tum, Mueang, Maha Sarakham (16°10'32.2"N, 103°26'59.6"E). CUMZ 00387, one spm., Wat Pa Khok Hinlat, Mueang, Maha Sarakham (16°10'4.82"N, 103°28'57.255"E). CUMZ 00287, one spm., Ban Phon Thong, Kaset Wisai, Roi-Et (15°39'59.6"N, 103°33'10.9"E). CUMZ 00369, one spm., Wat Pa Ka Thon, Wang Nam Khiao, Nakhon Ratchasima (14°24'38.479"N, 101°41'3.581"E). CUMZ 00370, two spms., Wat Tham Phrommachan Thamma Ram, Pak Chong, Nakhon Ratchasima (14°34'29.046"N, 101°16'39.524"E). CUMZ 00388, five spms., Khao Kradong Forest Park, Buriram (14°56'26.209"N, 103°5'21.1"E). CUMZ 00389, one spm., Phanom Sawai Forest Park, Surin (14°45'39.325"N, 103°22'2.067"E). CUMZ 00390, one spm., Mueang, Surin (14°52'9.914"N, 103°30'21.877"E). CUMZ 00284, one spm., and CUMZ 00391, six spms., Pa Son Nongkhu, Sangkhla, Surin (14°40'55.7"N, 103°45'51.9"E). CUMZ 00248, two spms., Kaeng Lamduan, Ubon Ratchathani (14°26'15.0"N, 105°06'06.7"E). CUMZ 00279, one spm., Nang Rong Waterfall, Nakhon Nayok (14°19'52.5"N, 101°19'09.1"E). CUMZ 00252, one spm., Si Chang Island, Chonburi (13°09'08.1"N, 100°48'29.3"E). CUMZ 00258, two spms., Wat Khao Maidaeng, Sriracha, Chonburi (12°56'56.0"N, 101°02'11.9"E). CUMZ 00320, one spm., Lan Island, Chonburi (12°55'05.8"N, 100°46'43.8"E). CUMZ 00285, one spm., Juang Island, Sattahip, Chonburi (12°31'46.4"N, 100°57'18.4"E). CUMZ 00269, two spms., Samet Island, Ban Phe, Rayong (12°34'04.3"N, 101°27'23.3"E). CUMZ 00291, one spm., Wat Khao Sarp, Rayong (12°36'46.8"N, 101°23'18.8"E). CUMZ 00254, one spm., Wat Khao Chakan, Sa Kaeo (13°39'38.0"N, 102°05'02.7"E). CUMZ 00366, one spm., Wat Tham Khao Phrachan, Khao Chakan, Sa Kaeo (13°34'37.66"N, 102°5'39.42"E). CUMZ 00367, one spm., Tham Phet Pho Thong, Khlong Hat, Sa Kaeo (13°39'15.671"N, 102°29'28.066"E). CUMZ 00368, two spms., Wat Khao Phrom Suwan, Watthana Nakhon, Sa Kaeo (13°54'10.886"N, 102°30'49.344"E). CUMZ 00322, one spm., Tha Sen Waterfall, Trad (12°07'59.1"N, 102°42'22.6"E). CUMZ 00360, seven spms., Wat Khao Ma Rong, Bang Saphan, Prachuab Khiri Khan (11°12'23.041"N, 99°30'1.442"E). CUMZ 00326, one spm., Kreab Cave, Langsuan, Chumphon (9°49'01.8"N, 99°02'15.6"E). CUMZ 00359, one spm., Ban Tham Thong, Pathio, Chumphon (10°55'59.984"N, 99°29'27.521"E).CUMZ 00293, one spm., Tham Khao Kriab, Pathio, Chumphon (9°49'01.3"N, 99°02'17.9"E). CUMZ 00354, one spm., Tham Khao Phlu, Lamae, Chumphon (9°43'41.103"N, 99°6'10.996"E). CUMZ 00355, one spm., Wat Tham Khao Kriap, Chumphon (9°48'58.347"N, 99°2'10.364"E). CUMZ 00356, one spm., Ton Phet Waterfall, Kapong, Ranong (9°43'36.056"N, 98°37'7.853"E). CUMZ 00357, one spm., Wat Khao Sap, Ranong (9°57'50.532"N, 98°38'28.765"E) CUMZ 00358, one spm., Bok Krai Waterfall, Ranong (10°22'31.47"N, 98°51'15.943"E). CUMZ 00278, one spm., Bok Krai Waterfall, Kraburi, Ranong (10°22'34.6"N, 98°51'18.3"E). CUMZ 00262, one spm., King Rama V Stone park, Kraburi, Ranong (10°29'36.7"N, 98°54'35.7"E).CUMZ 00251, three spms., Sai Rung Waterfall, Takua Pa, Phang Nga (7°26'26.2"N, 99°48'47.5"E). CUMZ 00261, one spm., Surin Islands, Phang Nga (9°26'58.4"N, 97°52'37.8"E). CUMZ 00273, two spms., Khao Phlai Dam, Sichon, Nakhon Si Thammarat (9°05'33.4"N, 99°54'25.0"E). CUMZ 00281, two spms., Klong Phot Waterfall, Nop Phitam, Nakhon Si Thammarat (7°48'37.8"N, 99°12'20.0"E). CUMZ 00351, one spm., Wat Tha Li-Phong, Chian Yai, Nakhon Si Thammarat (8°5'20.669"N, 100°7'59.627"E). CUMZ 00352, one spm., Tham Khamin, Ban Na San, Surat Thani (8°49'9.678"N, 99°22'19.029"E). CUMZ 00265, one spm., Kao Sok Resort, Phanom, Surat Thani (8°54'18.9"N, 98°31'21.1"E). CUMZ 00353, two spms., Ban Laem Thong, Chaiya, Surat Thani (9°24'33.236"N, 99°17'37.035"E). CUMZ 00267, one spm., Wat Huai To, Mueang, Krabi (8°13'37.6"N, 98°53'01.7"E). CUMZ 00263, two spms., Lanta Island, Krabi (7°29'55.7"N, 99°05'20.4"E). CUMZ 00283, 10 spms., Rog Nai Island, Koh Lanta, Krabi (7°13'12.7"N, 99°04'12.7"E). CUMZ CUMZ 00260, one spm., Wat Tham Suea, Krabi (8°07'26.1"N, 98°55'27.2"E). CUMZ 00250, two spms., Ron Waterfall, Khlong Thom, Krabi (7°56'07.4"N, 99°12'37.5"E). CUMZ 00255, one spm., Tham Rue Si, Kantang, Trang (7°28'35.9"N, 99°29'04.0"E). CUMZ 00259, one spm., Chao Mai Beach, Sikao, Trang (7°26'27.8"N, 99°20'45.0"E). CUMZ 00350, two spms., Thung Khai Botanical Garden, Trang (7°28'6.826"N, 99°38'8.117"E). CUMZ 00346, one spm., Wat Tham Khao Chin, Satun (6°38'33.161"N, 100°5'8.924"E). CUMZ 00347, one spm., Tham Ton Din, Kuan Don, Satun (6°43'16.848"N, 100°9'53.819"E). CUMZ 00348, one spm., Boriphat Waterfall, Rata Phum, Songkhla (6°59'41.991"N, 100°8'47.947'E). CUMZ 00349, one spm., Wat Hua Khao Chaison, Phatthalung (7°26'11.723"N, 100°7'44.344"E) 00264, one spm., Tham Malai, Phatthalung (7°38'58.3"N, 100°06'23.7"E). CUMZ 00244, two spms., Wat Tham Phuthakodom, Sinakharin, Phatthalung (7°33'37.2"N, 99°53'07.3"E). CUMZ 00274, five spms., Tham Wang Thong, Khuan Kanun, Phatthalung (7°40'55.1"N, 100°00'56.8"E). CUMZ 00280, one spm., Tham Su Mano, Srinakarin, Phatthalung (7°35'12.3"N, 99°52'04.3"E). NHMUK, one spm., Trang, leg. Dr. David A. Wamell. NHMUK, two spms., Lamphun. NHMUK 1898.4.5-28.32, two spms., Bangkok, two spms., Ko Si Chang Island and one spm., Dong Phaya Fai, leg. S.S. Flower. NHMUK 1898.6.28.17-18, two spms., Kabin, Siam [Sa Kaeo, Thailand], leg. H. Way. NHMUK, one spm., Siam [Thailand], leg. W.H. Hillman. NHMW Inv. No. 774, one spm., Xieng-Zang near Mekong, Hinter-Indien [Chiang Saen, Chiang Rai, Thailand], January 1886.

**Laos** — CUMZ 00335, one spm., Ban Bun Tai, Phongsaly (21°24'18.148"N, 101°57'27.294"E). CUMZ 00396, one spm., Mueang Phong Sali, Phongsaly (21°30'26.611"N, 102°10'27.28"E). CUMZ 00399, one spm., Luang Prabang (19°53'10.2"N, 102°08'16.2"E). CUMZ 00246, one spm., Ban Xai Na Pho, Xekong (15°25'28.3"N, 106°36'34.8"E). CUMZ 00245, one spm., Ban Ka Soam, Attapue (14°49'15.8"N, 106°49'05.0"E). CUMZ 00332, one spm., Thung Hin Tang 39 km from Vietnamese border, Attapue (14°45'57.353"N, 107°7'55.516"E). CUMZ 00397, one spm., Tad-Fan, Pakse, Champasak (15°11'25.735"N, 106°7'39.408"E). CUMZ 00398, one spm., Khon Phapaeng Waterfall, Champasak (13°56'53.2"N, 105°56'27.1"E).

**Cambodia** — CUMZ 00330, one spm., Angkor Wat, Siem Reap (13°24'45.5"N, 103°52'14.7"E). CUMZ 00331, one spm., Wat Tham Ban Kele, Srisophon (13°36'05.5"N, 102°57'09.3"E).

**Myanmar** — NHMUK 1889.7.15.2, 16 juveniles and one adult spms., Thayelinyo, Burma, leg. E.W. Oates. NHMUK, eight spms., Mandalay, Burma, leg. Oates. NHMUK, one juvenile spm., Cqimana, Uflu, Burma [undetermined]. NHMUK 1889.7.15.10, one spm., Moulmein, Burma [Mawlamyaing District, Mon State], leg. E.W. Oates. NHMUK 1889.7.15.3, five spms., Tondwingyi, Burma. NHMUK 1889.5.15.5, one spm., Teikjyi near Rangoon [Yangon], Burma. NHMUK, two spms., Rangoon [Yangon], Burma, leg. Oates. NHMUK, one spm., Owen Island, Mergui Archipelago, Burma, leg. Dr. Anderson. NHMUK 1889.5.15.6, one spm., Ihawawaddy River [Irrawaddy River], Burma, leg. E.W. Oates (Cpt). NHMUK 1889.5.15.7, one spm., South Tenasserim Range, Burma, leg. E.W. Oates.

**Malaysia** — NHMUK 1885.8.15.3.6, two spms., Kualin, Kedah, Malaysia, leg. S.S. Flower. NHMW Inv. No. 773, one spm., Penang, Siam [now Malaysia], leg. Skind, 1/2/1882. CUMZ 00336, one spm., Gumpung Baru, Gunung Genting, Perak (4°41'39.9"N, 100°52'46.0"E). CUMZ 00338, one spm., Klinic Desa, Kampung Panit Luar, Perak (4°56'17.9"N, 100°59'00.1"E). NHMUK 1887.71, fifty-two spms., Perak, Malaysia, leg. L. Way Esq. NHMUK 1889.5.26.1, one spm., Perak, Malaysia, leg. J.H. Leech. CUMZ 00337, Gua Musang, Kelantan (4°52'11.3"N, 102°00'40.6"E). NHMUK 1904.7.14.1-3, two spms., Kuala Kenting, Kelantan, Malaysia don. Imperial Institute. NHMUK(E) 200054 Chilo.1952.9.8.616, one spm., 2000 ft. in Koyan Forest, Sarawak, Oxford University Sarawak Expedition.

**Singapore** — NHMUK, one spm., Singapore, labelled “Scolopendra subspinipes var. concolor”, by Senniry. NHMW Inv. No. 775, one spm., Singapore, 1/1/1887, Aurora Expedition, leg. Swoboda, det. Attems C.

**Indonesia** — NHMUK 1893.5.13.2.30, two spms., east coast of Sumatra, Indonesia, leg. Mrs. Findlay. NHMW Inv. No. 8603, one spm., Java, Indonesia, with old label “Soumarak near Java”, leg. Plason A., det. Attems C., 1874. NHMW Inv. No. 772, one spm., Java, 1882. NHMW Inv. No. 8602, one spm., West Java, leg. Ausbeute Kainy, det. Attems C. NHMW Inv. No. 37U, one spm., Java, Novara Expedition. NHMW, one spm., Java, 1/2/1882, leg. Breitensl. NHMW Inv. No. 1648, one spm., Java, 1/5/1879, don. Moscovics. NHMW Inv. No. 8604, one spm., Java, Indonesia, don. Moscovics, det. Attems C. in 1/5/1879. NHMW Inv. No. 8600, one spm., Buitenzorg [Bogor], Java, Indonesia, leg. Müller, det. Attems C. NHMW Inv. No. 8605, six spms., Java, Indonesia. NHMW Inv. No. 8598, one spm., Java, Indonesia, leg. Adensamer, det. Attems C. NHMW Inv. No. 778, one spm., Java, Indonesia, don. Moscovics, det. Attems C. NHMW Inv. No. 779, one spm., Java, Indonesia, Novara Expedition.

**China** — NHMUK, one spm., Hong Kong by G. Browning.

**Indian Territory** — NHMW Inv. No. 768, one spm., India or Aracan, leg. Stoliczka in 1873. NHMUK, one spm., Chamba, Himalaya in Schlogin.

**Mexico** — NHMUK, one spm., Mexico, leg. California Academy of Science [probably introduced, if not mislabeled].

**Undetermined locality** — NHMUK, 1894.8.23.7, one spm., unknown locality, leg. C. Hose Esq. NHMUK 1913.6.18.903, two spms., Côte Malaga, Koch’s collection; NHMUK, one spm., near mouth of Patam R. [possibly Karandag (Patam) River, Bangladesh].

##### Diagnosis.

17–21 antennal articles, 6 basal articles glabrous dorsally. Each tooth-plate with 5–6 teeth. Tergites 3(4)–20 with paramedian sutures. Complete tergite margination from T7. Tergite of ultimate leg-bearing segment without depression or suture. Complete or incomplete paramedian sutures on sternites. Coxopleural process with two apical spines, absent lateral and dorsal spines. Ultimate leg prefemora with 0–1 M, 0–1 DM and prefemoral process with 1–4 spines. One tarsal spur on legs 1–19(20).

##### Composite description.

Body length up to more than 25 cm (collections from Java, NHMW). Varied colouration; cephalic plate and segments monochromatic. Tergites usually brownish-orange with or without dark band on posterior border of tergites. Cephalic plate with small punctate on anterior part; median sulcus present. Posterior part of cephalic plate without paramedian sulci.

Antenna usually with 18 articles (17, 19 and 21 articles in some specimens), basal 6 subcylindrical and glabrous dorsally (Fig. 21A), 5–5.5 glabrous ventrally. Antennae reach segment 3–4. Forcipular trochanteroprefemoral process bearing denticles in two groups, one apical and 2–3 inner. Tooth-plates wider than long or nearly equivalent, 4–5 teeth on each side (Figs 21B, 23C–D; rarely 3, 7, 8 or 10). Tooth-plate with straight, transverse basal suture. Coxosternite smooth, without median suture (Fig. 21C, 23A). Article 2 of second maxillary telopodite with spur.

**Figure 19.**
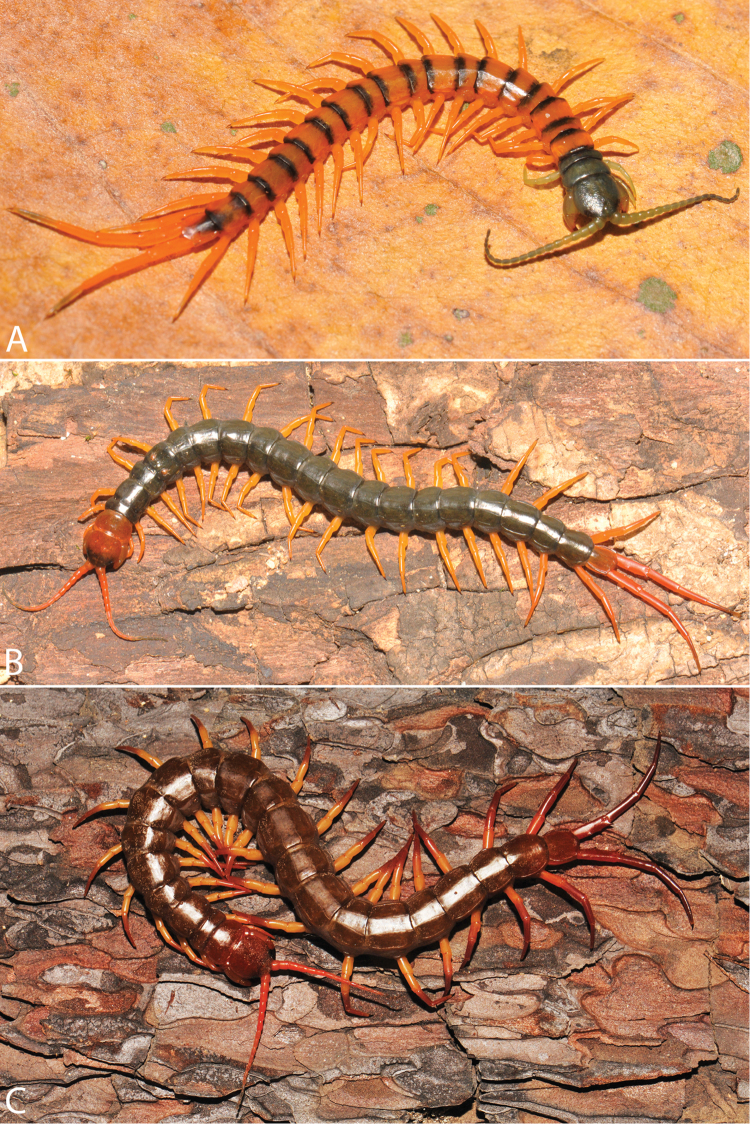
Colouration changes and patterns during developmental stages of *Scolopendra dehaani*: **A** Juvenile stage **B** Sub-adult stage **C** Adult stage (specimen from northern Thailand).

**Figure 20.**
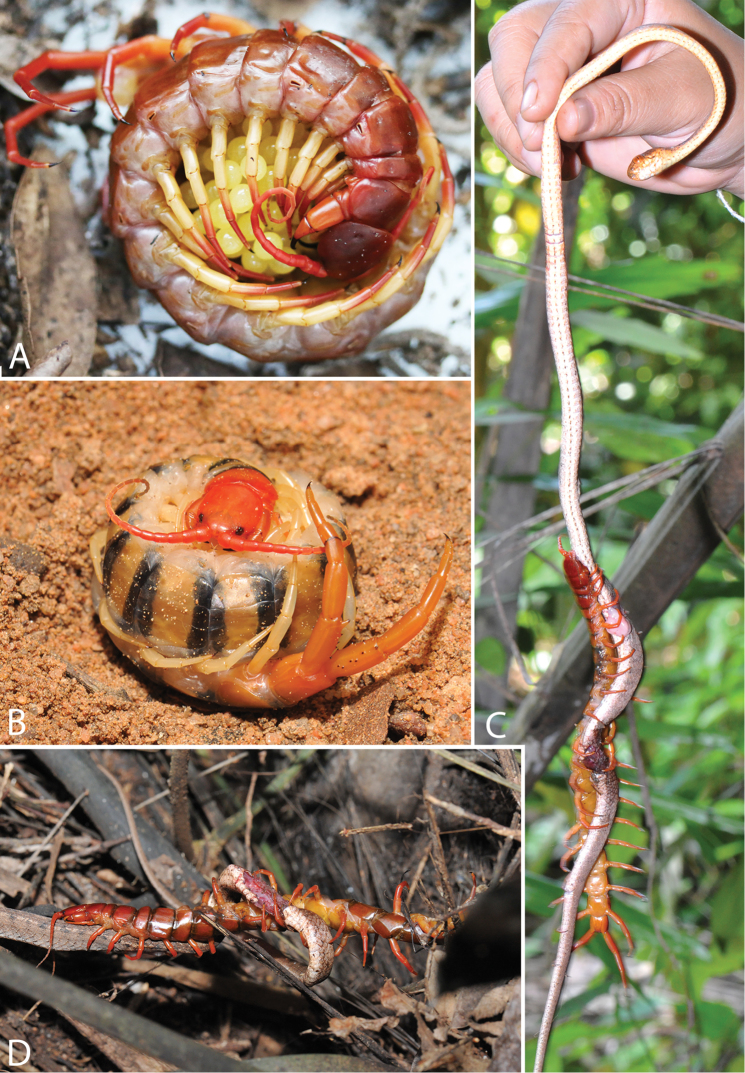
Brooding and feeding behaviours: **A** *Scolopendra dehaani* exhibiting simple coiling with cluster of embryonic stadia (photograph by Natdanai Likhitrakarn) **B** *Scolopendra morsitans* exhibiting double coiling with post-embryonic stadia **C–D** *Scolopendra dehaani* preying on snail-eating snake *Pareas carinatus* **D** Flexibility of trunk segments during predation.

**Figure 21.**
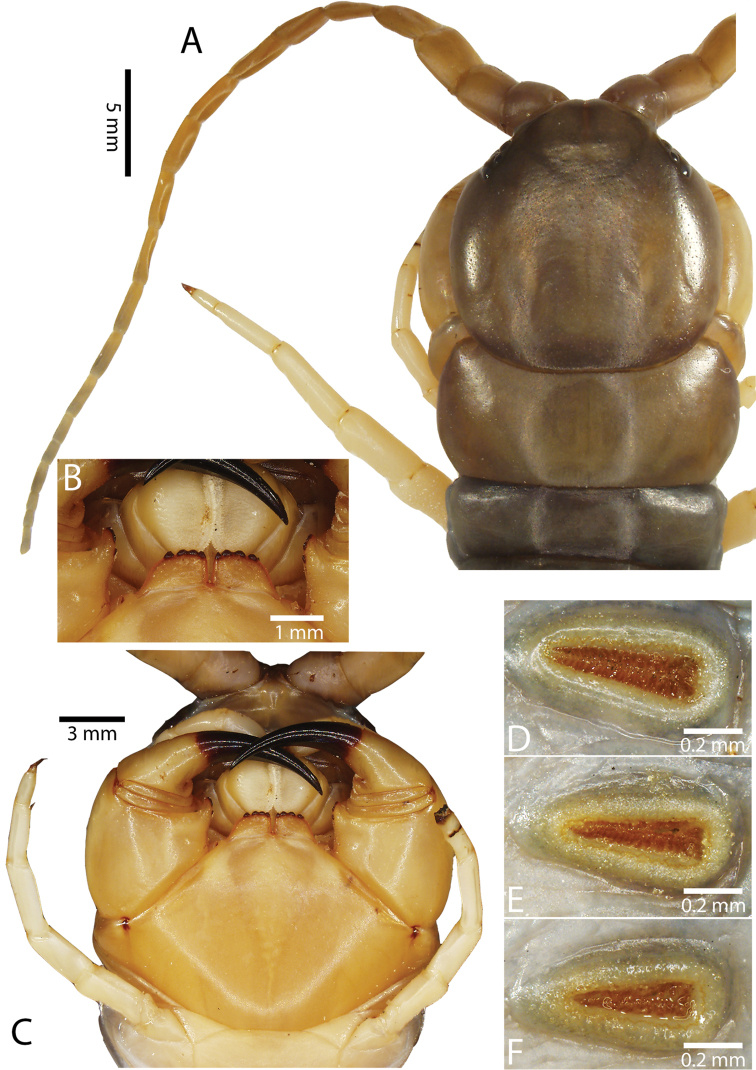
*Scolopendra dehaani* (CUMZ 00282): **A** Cephalic plate and trunk segments 1–2 **B** Tooth-plates **C** Forcipular segment **D–E** Spiracles 3, 5 and 8, respectively.

**Figure 22.**
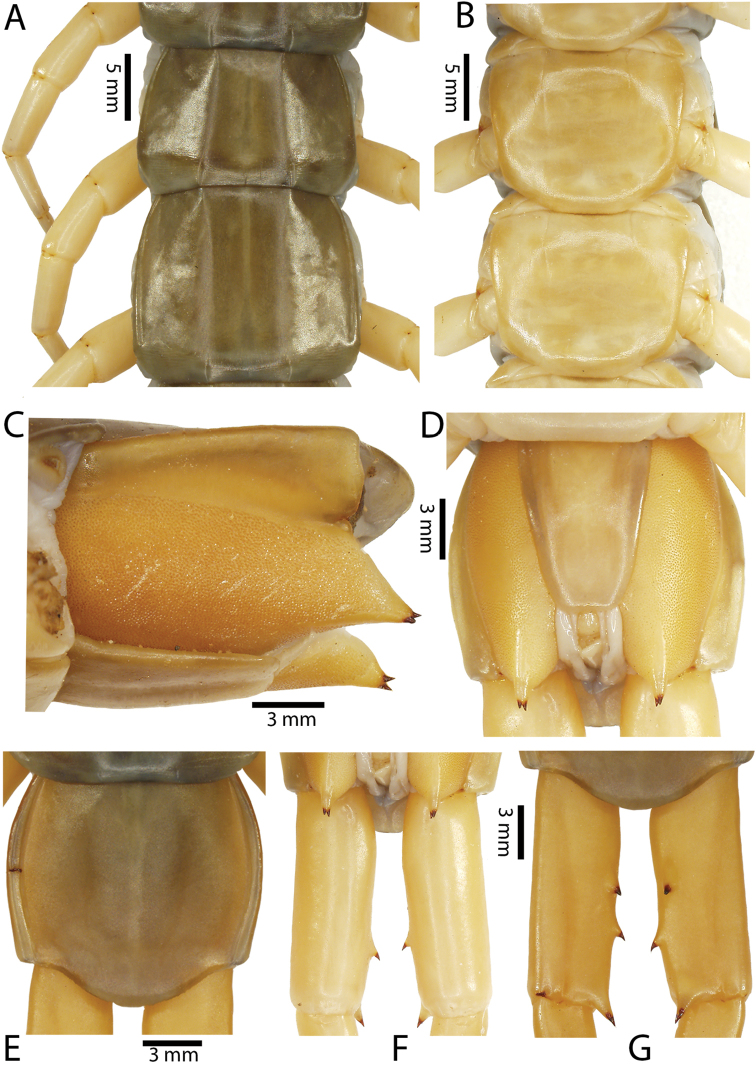
*Scolopendra dehaani* (CUMZ 00268 and 00282): **A** Tergites 10–11 **B** Sternites 10–11 **C** Lateral view of coxopleuron **D** Sternite of ultimate leg-bearing segment and coxopleura **E** Tergite of ultimate leg-bearing segment **F–G** ventral and dorsal view of ultimate leg prefemora.

**Figure 23.**
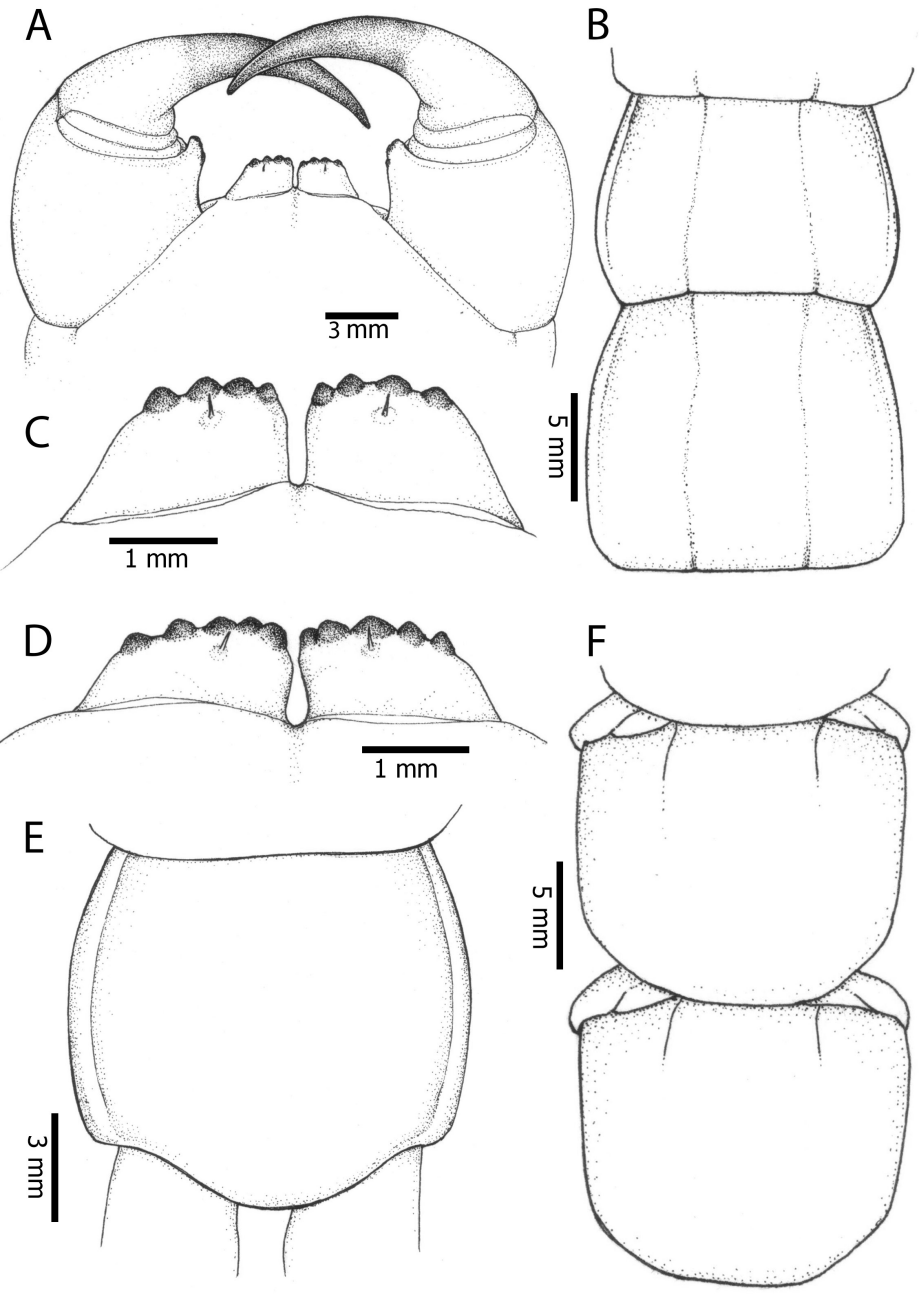
*Scolopendra dehaani* (CUMZ 00365): **A** Forcipular segment **B** Tergites 9 and 10 **C–D** Variation in teeth on tooth-plates **E** Tergite of ultimate leg-bearing segment **F** Sternites 9 and 10.

**Figure 24.**
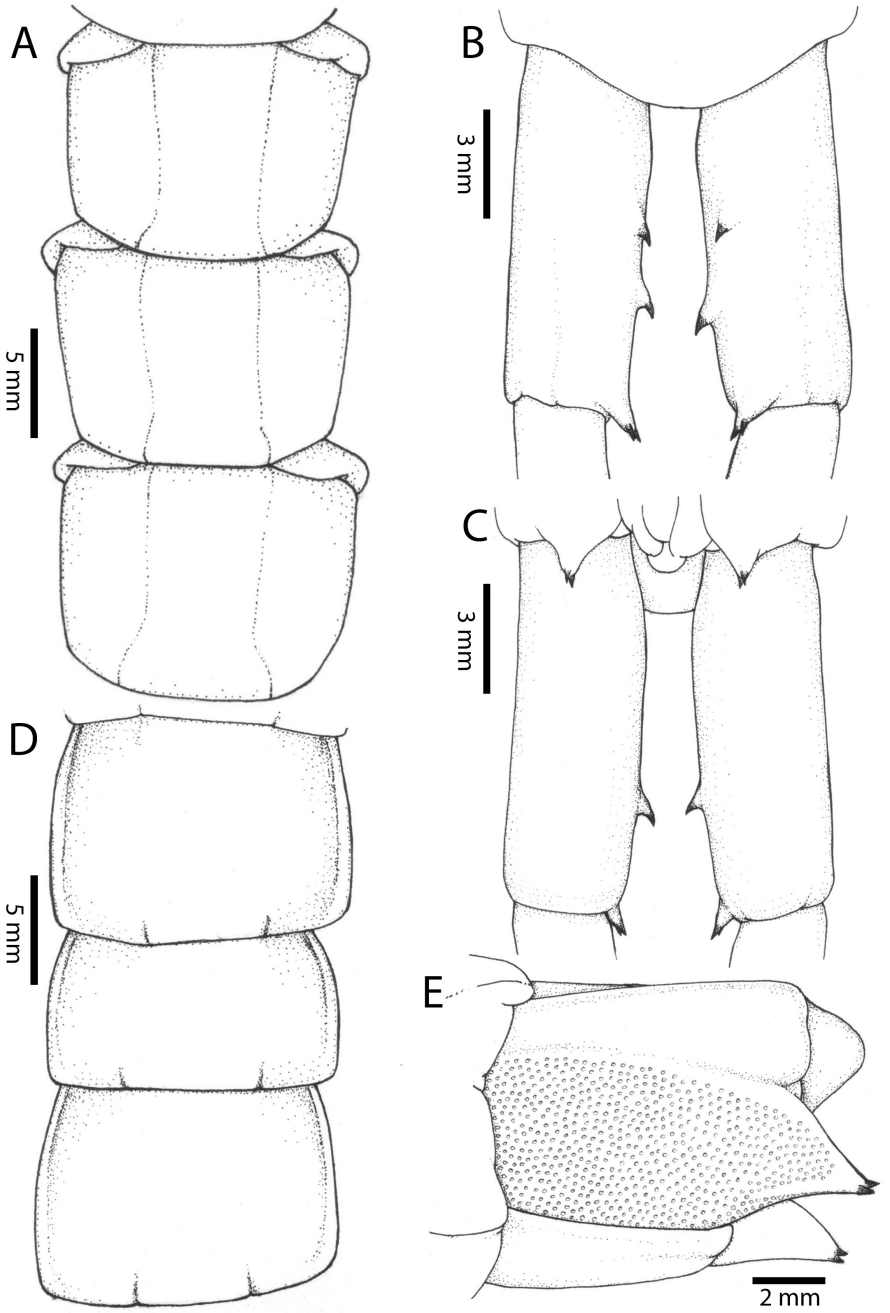
*Scolopendra dehaani* (CUMZ 00365): **A** Sternites 9–11 **B–C** Spines on ultimate leg prefemora (dorsal and ventral views, respectively) **D** Tergite 9–11 **E** Lateral view of coxopleuron.

Anterior margin of T1 underlying cephalic plate (Fig. 21A). Complete paramedian sutures from T4; margination typically starting on TT9–12 (14 in one spm. from Burma; NHMUK 1889.5.1.7). Tergite surface (Figs 22A, 23B, 24D) smooth, without median sulci. Tergite of ultimate leg-bearing segment (Figs 22E, 23E) curved posteriorly, without median furrow or depression; ratio of width: length of tergite of ultimate leg-bearing segment 0.84:1. Paramedian sutures usually complete on sternites (Figs 22B, 24A), atypically confined to anterior 30–50% (Fig. 23F). Surface of sternite smooth (atypically with small pit on posterior part). Sternite of ultimate leg-bearing segment (Fig. 22D) with sides converging posteriorly; surface of sternite without depression. Pore-field on coxopleuron terminating well beneath margin of tergite of ultimate leg-bearing segment, anterior part of pore area widest (Figs 22C, 24E).

Coxopleural process moderately long or short with two apical spines (atypically 0–1 spines); pore-free area extending 90–100% length from distal part of coxopleural process to margin of sternite of ultimate leg-bearing segment (Fig. 22D).

All legs without setae and tibial spur. One tarsal spur on legs 1–19 or 20 in equal frequency. Ultimate legs: moderately long and slender, with ratios of lengths of prefemur and femur 1.1:1, femur and tibia 1.2:1, tibia and tarsus 2 1.6:1.; tarsus 1 and tarsus 2 2.3:1. Prefemora long and slender, flattened dorsally (Fig. 22G). Prefemoral spines (Figs 22F–G, 24B–C): 0 VL, 0–1 M, 0–1 DM and prefemoral process with 1–4 spines. Posterior margin of prefemur with shallow median groove.

**Figure 25.**
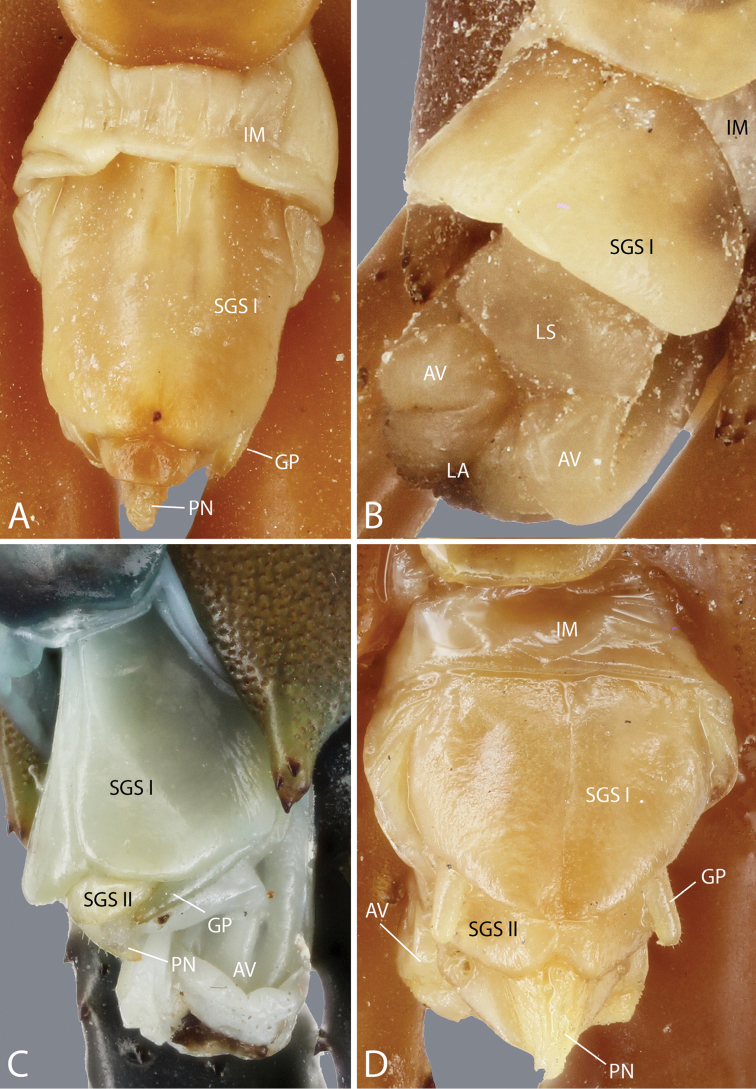
Genital segment(s) in some preserved *Scolopendra* specimens: **A** *Scolopendra dehaani* (male) **B** *Scolopendra calcarata* (female) **C** *Scolopendra pinguis* (male) **D** *Scolopendra morsitans* (male).

Genital segments well developed, reaching longer than distance between posterior margin of sternite of ultimate leg-bearing segment and distal part of coxopleural process. Sternite of genital segment 1 round and convex posteriorly, with median suture (Figs 7A, 25A). In male, sternite of genital segment 2 attached to penis. Tergites of genital segments without small setae. Gonopods with or without small setae. Penis with apical bristle.

**Colouration.** This species is among those that exhibited the most varied colouration patterns. Chao (2008) suggested two major types of *Scolopendra* colouration, referred to as monochromatic and dichromatic. The variability in colouration of *Scolopendra dehaani* has been reported in Southeast Asia over the past century (Flower 1901). In the present study, the colour patterns of growth stages have been photographed (Fig. 19). Recently, ontogenetic variation including specific changes in colouration has been discussed with reference to geographical distribution (Siriwut et al. 2015a). These findings indicated that the colouration change might involve a heritable component among populations. A descriptive classification of colouration in *Scolopendra dehaani* is as follows:

**Colour morph 1**: Dichromatic. All segments including cephalic plate dark brownish. Posterior border of tergites with a dark band. Antenna reddish brown. Pleuron with pale grey integument, all pleurites brownish. Legs chestnut brown, tibiae and tarsi dark purplish.

**Colour morph 2**: Dichromatic. All segments brown or yellowish orange. Posterior border of tergites with a dark band. Antenna yellowish orange. Pleuron with pale grey integument, pleurites pale grey. Legs dark brownish, tibiae and tarsi dark purplish.

**Colour morph 3**: Monochromatic. All segments including cephalic plate reddish brown. Antenna reddish brown. Pleuron with pale grey integument, pleurites brownish. Legs 1–18 yellowish, tibiae and tarsi reddish brown. Leg 20 and ultimate legs entirely reddish brown.

**Colour morph 4**: Dichromatic. Cephalic plate, TT1–2 and 19–21 bright reddish, rest of tergites brown. Posterior border of tergites with a dark band. Antenna yellowish or bright orange. Pleuron with pale grey integument, pleurites orange. Legs 1–19 yellowish, without secondary colouration on distal part. Leg 20 and ultimate legs entirely reddish.

**Colour morph 5**: Dichromatic. All segments including cephalic plate cherry reddish. Posterior border of tergites with a dark band. Antenna reddish or orange. Pleuron with grey integument, pleurites orange. All legs reddish.

##### Discussion.

This is the largest centipede in Asia. A consistent character that is treated as diagnostic for this species is the absence of ventral spines on the ultimate leg prefemur. *Scolopendra dehaani* possesses characters of the *Scolopendra subspinipes* s.l. sensu Lewis (2010) but after morphological survey (Kronmüller 2012) and molecular delimitation (Siriwut et al. 2015b) a species rank has been conferred to this name. The morphological variability in the extent of paramedian sutures recorded in some specimens might demand further examination. Three morphotypes of *Scolopendra dehaani* within the examined collections can de delimited as follow:

**Morphotype 1**: Complete paramedian sutures on tergites and sternites. This is the typical form of *Scolopendra dehaani*, according to Attems (1930b) and Jangi and Dass (1984), observed throughout the geographic range of the species.

**Morphotype 2**: Paramedian sutures complete on tergites, confined to 20–30% length of sternites. This morphotype is observed in a specimen apparently from Bangladesh.

**Morphotype 3**: Paramedian sutures lacking on tergites on all segments (Fig. 24D) and confined to 10–20% length of sternites (Fig. 23F). This morphotype has been observed in spec. from Hong Kong and northern India and is common in Java.

Based on these morphotypes, we surmise that this variability might have a geographic basis and could suggest evidence of cryptic speciation. Molecular data are presently lacking for morphotype 3 in particular and, presently, we apply the specific name *Scolopendra dehaani* Brandt, 1840, throughout the entire geographic range. A morphological comparison through the species’ geographical range is given in Table 7. In addition, we recorded some brooding and feeding behaviour of individuals in their natural habitat. *Scolopendra dehaani* exhibited double coiling when guarding offspring in the brood chamber (Fig. 20A), similar to *Scolopendra morsitans* (Fig. 20B). Two stages of feeding behaviour have been observed, which may be described as late foraging (Fig. 20D) and consumption stages (Fig. 20C). The centipede attached itself to the posterior part of the body of the snail-eating snake *Pareas carinatus* Boie, 1828 and then advanced to the anterior part of the prey’s body, stabbing the snake several times. The posterior part of the centipede hung twisted with a palm trunk by using the locomotory and ultimate legs. The period of consumption of the prey lasted approximately one to two hours after the initial recording; all somatic tissue was completely eaten by the centipede.

**Table 7.** Morphological survey of geographical variation of *Scolopendra dehaani* populations from different regions.

Characters	Geographical distribution area						
	Java^1,2^	Thailand, Laos and Cambodia^1^	Burma^1^	Malay Peninsula^1^	India^3^	China- Japan^1^	Mexico^1^
Number of antennal articles	14–18	18–21	17–19	17–19	18–19	18	18
Number of glabrous articles	6	6	6	6	6	6	6
Teeth on tooth-plate	5+4,5+5	5+5	5+5,5+6,6+6	5+5,6+6,7+8,8+10	6+6	6+7	6+6
First tergite with complete paramedian sutures	incomplete	4	2–6	3–4	4	3–5 or incomplete	incomplete
First tergite with margination	7–10	13	10–14	7–13	5	8	7
Tergite surface	smooth	smooth	smooth	smooth	smooth	smooth	smooth
Median furrow on tergite of ULBS	absent	absent	absent	absent	absent	absent	absent
Extent (percentage) of paramedian sutures on sternites	10–15% (rarely with complete PS)	90–100%	100%	30–100%	80–100%	80–100%	20%
Sternite of ultimate leg-bearing segment	with or without pit- like median depression	with pit- like median depression	with or without pit- like median depression	with or without pit-like median depression	without pit-like median depression	without pit-like median depression	without pit-like median depression
Spines on coxopleural process	AP: 2 SAP: 0–1	AP: 2	AP: 2	AP: 0–2	AP: 2	AP: 2	AP: 1 SAP: 0–1
Spinulation formula on prefemora of ult. legs	M: 0–2 DM: 0–2 SP: 2–4	M: 1 DM: 1 SP: 2–3	M: 0–2 DM: 0–2 SP: 2	M: 1 DM: 1 SP: 1–3	DM: 1 SP: 2	M: 0–1 DM: 1–2 SP: 1–2	M: 0–1 DM: 1 SP: 2
Legs with one tarsal spur	1–20	1–19	1–19 (20)	1–19 (20)	1–20	1–20	1–20

**Note**: each superscript number refers to data from recent and earlier studies as follow: ^1^ = This study, ^2^ = Brandt (1840), ^3^ = Jangi and Dass (1984).

One new subjective synonym is proposed for *Scolopendra dehaani*. The holotype and sole known specimen of *Scolopendra arborea* Lewis, 1982 (NHMUK 1952.9.8.616) is approximately 40 mm in length, from an elevation of 2,000 ft. at Koyan Forest, Sarawak. It is here regarded as an immature specimen of *Scolopendra dehaani*. Lewis (1982, 2010) noted that morphological characteristics of *Scolopendra arborea* are similar to some other Asian and one Pacific island species, namely *Scolopendra dehaani*, *Scolopendra puensis* Jangi & Dass, 1984, *Scolopendra gracillima*, *Scolopendra hardwickei* Newport, 1844, and *Scolopendra metuenda* Pocock, 1895. In its original description, *Scolopendra arborea* was noted to have a colouration pattern similar to juveniles of *Scolopendra dehaani*, i.e., the cephalic plate and last two segments black, the other tergites bright orange, the legs bright blue with blackish lateral lines. Its taxonomic characters appear very close to *Scolopendra dehaani*, the apparent differences being the number of spines on the coxopleural process (one in *Scolopendra arborea* versus two in *Scolopendra dehaani*) and tergite margination starting from T20 or only T21 margination (reaching more forward to anterior segments in *Scolopendra dehaani*). According to our study, the number of apical spines on the coxopleural process of adults of *Scolopendra dehaani* is quite strictly two, but on some occasions a single minute apical spine is present in small sub-adult and juvenile specimens. Morover, our re-examination of the holotype of *Scolopendra arborea* indicated that tergite margination starts from T15, which decreases the distinction from *Scolopendra dehaani*. An unusual morphological feature of *Scolopendra arborea* is the expansion of the peritrema on the spiracle of segment 3 to cover part of the tri-crescentic flaps. This atypical feature may be ontogenetic variation or the effect of muscle contraction or extension around the spiracle margin which might be affected by fixation. Without other significant diagnostic characters to distinguish this species from *Scolopendra dehaani*, which is abundant on the mainland and on some islands in Southeast Asia, we synonymise *Scolopendra arborea* with *Scolopendra dehaani*.

##### Distribution.

Widespread species in the Southeast Asian mainland and some islands (Fig. 18). The first occurrence was reported from Java, Indonesia. In this study, we provide all recorded localities of this species in Asian territory, together with some localities in which the species might be introduced, as follows: **Southeast Asia**: probably this is the native distribution area of this species according to population abundance and genetic structure. Population density is high throughout mainland territory especially in synanthropic areas but populations are more scattered in montane areas. The currently known distribution is as follows: Thailand (entirely), Laos (Vientiane, Khammouane, Phongsaly and Savannakhet), Cambodia (Siem Reap), Vietnam (fide Tran et al. 2013: Poulo Condore, Quang Binh, Nghe An, Dak Lak, Kon Tum, Ba Ria), Myanmar (Mergui Archipelago, Rangoon (Yagon) and Mandalay), Malaysia (Kelantan, Perak, Penang, Kedah, Selangor, Johore and Sarawak), Singapore, Indonesia (Kota Bogor (Java), Solok, Si-Rambe?, Singgalang Mountain (Sumatra), Anambas and Ambon islands) and Philippines. **East Asia**: China (Hong Kong and Hainan), Japan (Okinawa) and Taiwan (Hualien). **South Asia**: India (Himachal Pradesh (Chamba Himalaya), West Bengal (Calcutta), Karnakata, Andaman and Nicobar Islands), Bangladesh and Sri Lanka. **Central America**: Mexico (probably introduced).

#### Scolopendra multidens

Taxon classificationAnimaliaScolopendromorphaScolopendridae

Newport, 1844

**Figure 26.**
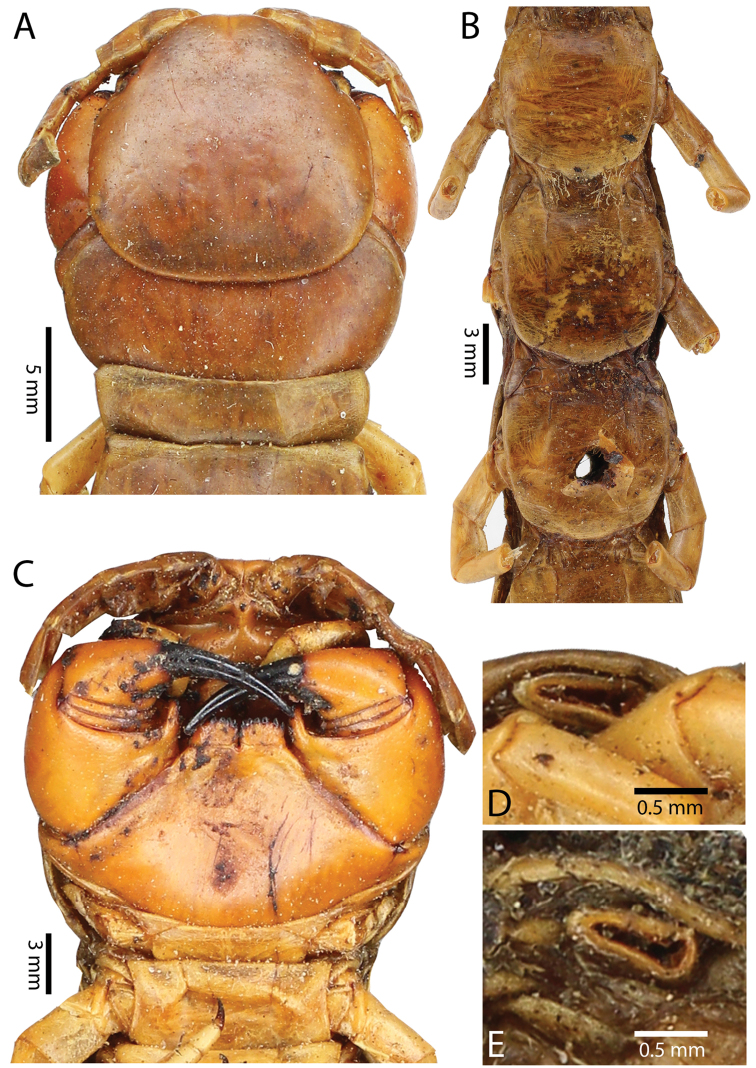
*Scolopendra multidens* (Holotype NHMUK): **A** Cephalic plate and trunk segments 1–3 **B** Sternites 9–11 **C** Forcipular segment **D–E** Spiracles 3 and 5, respectively.

**Figure 27.**
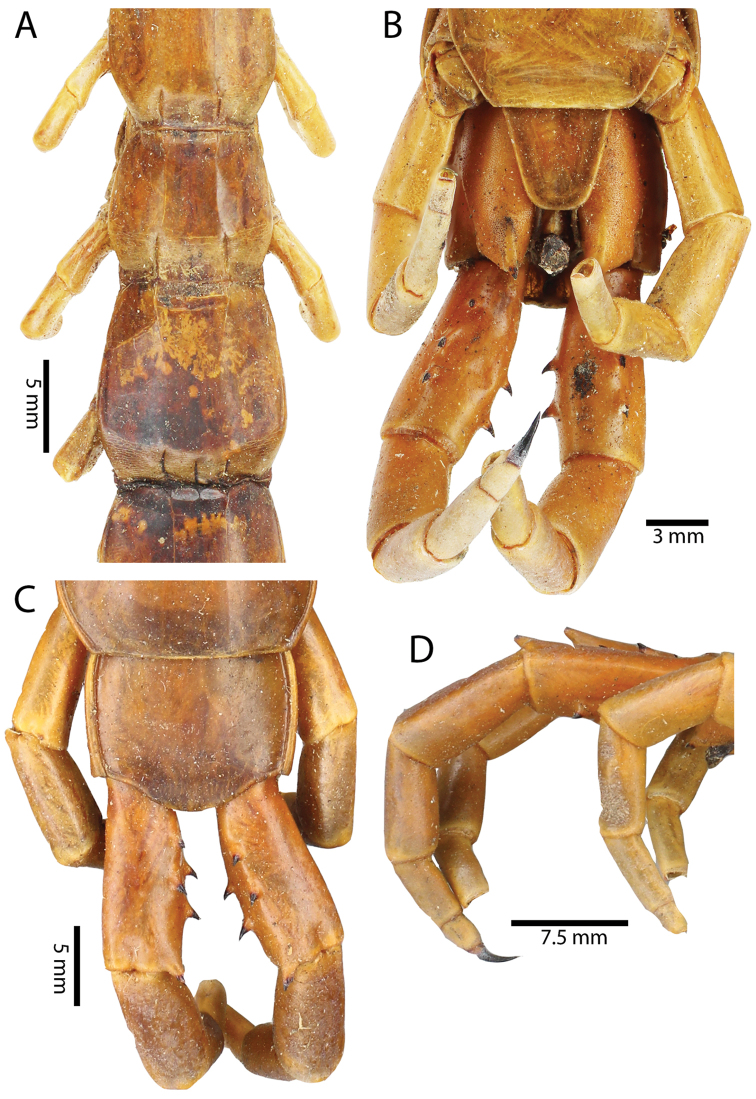
*Scolopendra multidens* (Holotype NHMUK): **A** Tergites 9–10 **B** Sternite of ultimate leg-bearing segment, coxopleura, legs 20 and ventral view of ultimate legs **C** Tergite of ultimate leg-bearing segment and dorsal view of leg 20 and ultimate legs **D** Lateral view of ultimate leg and legs 20.

**Figure 28.**
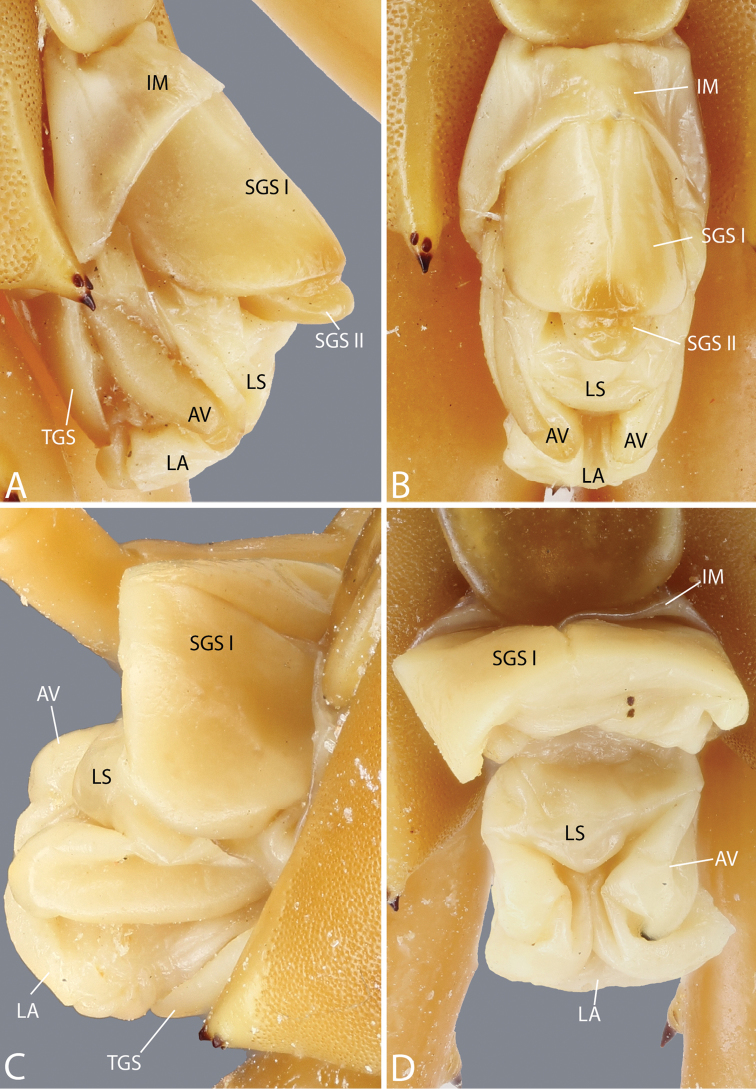
Genital segment(s) of **A–B** *Scolopendra multidens* (male; lateral and ventral views, respectively) **C–D** *Scolopendra cataracta* (female; lateral and ventral views, respectively).

**Figure 29.**
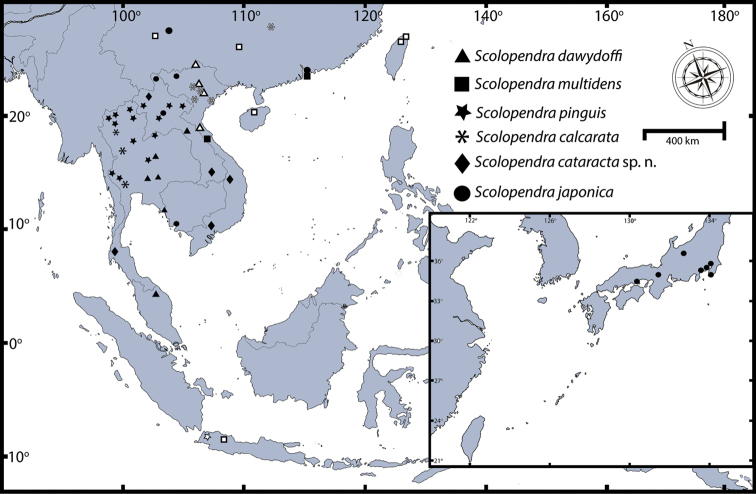
Distribution map of six *Scolopendra* species in Southeast Asia and China-Japan Sea (small map): Filled and blank colours refer to localities from the present study and in the literature, respectively.

Figs 26 , 27 , 28A–B , 29

Scolopendra multidens Newport, 1844: 97, 1845: 391. Gervais 1847: 288. Kohlrausch 1881: 101. Haase 1887: 46, pl. 3, fig. 46. Chao 2003: 7, 2008: 30, table 2, figs 31–36. Lewis 2010b: 108. Kronmüller 2012: 26. Siriwut et al. 2015a: 22.
Scolopendra rugosa Meinert, 1886: 202.
Scolopendra subspinipes multidens — Kraepelin 1903: 264. Attems 1907: 81, 1914a: 107, 1914b: 568, 1930b: 31. Muralewicz 1913: 201. Takakuwa 1942: 359, 1943: 171. Takashima 1949: 11. Takashima and Shinohara 1952: 4. Wang 1955: 16, 1956: 158, 1962: 101. Zhang 1992: 8, fig. 1.

##### Type locality.

Not designated.

##### Material.

**Holotype** NHMUK, one adult, dry condition, Newport’s collection (Figs 26, 27).

##### Additional material.

NHMUK, one spm., Qiang Binh, Vietnam (17.47001°N 106.38168°E), leg. F. Naggs and J. Ablett, 4/3/2012. NHMUK, one spm., Annam [Vietnam], leg. A. Graham (C.). NHMUK, one spm., in bottle “*Scolopendra multidens*”, Hong Kong, July 1954, leg. I.D. Romer. NHMUK, spm. numbers 2, 6, 8, 11, 13 and 14 in bottle “*Scolopendra multidens*”, Hong Kong.

##### Diagnosis.

17–18 antennal articles, 6 basal articles glabrous dorsally. Each tooth-plate with 5–10 teeth. Tergites 2(3)–20 with paramedian sutures. Complete tergite margination from TT12 (14)–21. Tergite of ultimate leg bearing segment without depression or suture. Paramedian sutures 20–60% on anterior part of sternites. Coxopleural process with 2–3 apical spines. Ultimate leg prefemora with 2 VL, 1 M, 1–2 DM and 1–3 spines on prefemoral process. One tarsal spur on legs 1–19.

##### Composite description.

Body length 11.4 cm in syntype. Dried holotype brownish on entire body. Cephalic plate with small punctae; median sulcus present. Posterior part of cephalic plate without paramedian sulci.

Antennae with 17–18 articles, basal 6 subcylindrical and glabrous dorsally on left side, 6 articles glabrous ventrally. Antennae reach segment 4. Forcipular trochanteroprefemoral process with denticles in two groups, one apical and three inner. Tooth-plates quadrate, with 5 teeth (Fig. 26C; total of 12–14 teeth in original description). Tooth-plate with straight, transverse basal suture. Coxosternite smooth, without median suture. Article 2 of second maxillary telopodite with spur.

Anterior margin of T1 underlying cephalic plate (Fig. 26A). Complete paramedian sutures on TT2; margination typically starting on T9 (T13 in specimen from Hong Kong; NHMUK). Tergite surface (Fig. 27A) smooth, with median sulci on posterior part. Tergite of ultimate leg-bearing segment relatively broad (Fig. 27C), curved posteriorly, without median furrow or depression; ratio of width: length of tergite of ultimate leg-bearing segment 0.72:1. Sternites (Fig. 26B) with short paramedian sutures on approximately 40–60% of anterior part. Surface of sternites smooth. Sternite of ultimate leg-bearing segment (Fig. 27B) with sides converging posteriorly, surface without depression. Pore-field on coxopleuron terminating beneath margin of tergite of ultimate leg-bearing segment, dorsal margin of pore area sinous.

Coxopleural process moderately long or short with 1–2 apical and 1–2 subapical spines; pore-free area extending 80% length from distal part of coxopleural process to margin of sternite of ultimate leg-bearing segment (Fig. 27B).

All legs without setae and tibial spur. One tarsal spur on legs 1–19; holotype lacking leg 20. Ultimate legs (Fig. 27B–D) thick and moderately long, with ratios of lengths of prefemur and femur 1.2:1, femur and tibia 1.7:1, tibia and tarsus 1 1.8:1; tarsus 1 and tarsus 2 1.5:1. Prefemora flattened dorsally, atypically rounded, with enlarged blackish spines. Prefemoral spines as follow: 3 VL, 2 M, 2 DM, prefemoral process with 3 spines (3 V, 2 D, prefemoral process with 3 spines in original description). Posterior margin of prefemur with shallow median groove.

Genital segments well developed, reaching longer than distance between posterior margin of sternite of ultimate leg-bearing segment and distal part of coxopleural process (Fig. 28A). Sternite of genital segment 1 round and convex posteriorly, with median suture (Fig. 28B). In male, sternite of genital segment 2 well-developed. Tergite of genital segment without small setae. Gonopods and penis absent (Fig. 28A–B).

**Colouration.** According to Chao (2008), juvenile specimens have a reddish orange cephalic plate and T1. Basal part of antenna reddish orange, distal part greenish. The remaining tergites dark green. All legs reddish orange. In adult, all tergites reddish orange.

##### Discussion.

Morphological characters are similar to *Scolopendra subspinipes* sensu Chao, 2008. The validity of *Scolopendra multidens* as a separate species was re-established by the absence of gonopods on the first genital segment in males (Chao 2008). Two species of *Scolopendra* in the region, *Scolopendra hainanum* Kronmüller, 2012 from Hainan Island, China, and *Scolopendra multidens*, distributed in eastern coastal Asia, have been reported to lack gonopods. Study of the type specimens of both *Scolopendra multidens* and *Scolopendra dawydoffi* indicates a close relationship between these two species. The lack of gonopods in males of *Scolopendra dawydoffi* from Thailand was found in the present study, which might provide a synapomorphic character for a clade composed of these species. However, the species boundaries between these taxa are complicated by disjunct distributional data, with previous records indicating that *Scolopendra multidens* mostly occurs in the East China Sea and possibly ranges as far as Japan. Vahtera et al. (2013) reported the occurrence of *Scolopendra multidens* from northern Vietnam and provided DNA sequences for a specimen. In this present study, we analyzed molecular data for numerous specimens of *Scolopendra dawydoffi* as well as the Vietnamese specimen of *Scolopendra multidens* to explore their relationships. The phylogenetic tree based on mitochondrial and nuclear genes indicated that *Scolopendra multidens* is sister taxon to *Scolopendra dawydoffi* and both are genetically distinct from other members of the *Scolopendra subspinipes* group (Fig. 1). Based on to these results, an absence of gonopods is corroborated as a synapomorphy for this clade. Only geographical distribution and molecular data (i.e., branch length) can be used to distinguish these two species. COI is the only molecular marker available for *Scolopendra multidens* from East Asia (unpublished results from sequences in GenBank from Taiwan), and analyses of our COI data with these included groups the Vietnamese specimen together with other *Scolopendra multidens*. Because of their genetic distinctness and reciprocal monophyly, we regard *Scolopendra multidens* and *Scolopendra dawydoffi* as valid species until further morphological examination throughout their distribution ranges has been done to clarify species boundaries.

##### Distribution.

Widespread species in Asia (Fig. 29). The original description did not designate a type locality. Kohlrausch (1881) reported the collecting locality of this species as China based on a specimen in the Godeffroy collection, Hamburg, Germany. Subsequent publications reported further localities of this species as follows: **Southeast Asia**: Vietnam, Philippines (Mindanao) and Indonesia (Java and north New Guinea?). **East Asia**: China (Yunnan, Guanxi, Hainan, Hong Kong and Taiwan (Keelung, Nuannuan and Taipei)).

#### Scolopendra calcarata

Taxon classificationAnimaliaScolopendromorphaScolopendridae

Porat, 1876

Figs 9B , 25B , 29 , 30 , 31 , 32 , 33 , 34 , 35

Scolopendra calcarata Porat, 1876: 10. Haase 1887: 51. Attems 1930b: 33. Zhang 1992: 6, map 1. Schileyko 1995: 77, fig. 3, 1998: 268, 2001: 434, 2007: 74. Lewis 2010b: 98. Tran et al. 2013: 227.

##### Type locality.

China.

##### Material.

**Holotype**: NHRS-KASI 000000042, one female with label “*Scolopendra calcarata* v. Por” from Kina [China] in Kinberg collection (Figs 32, 33).

**Figure 30.**
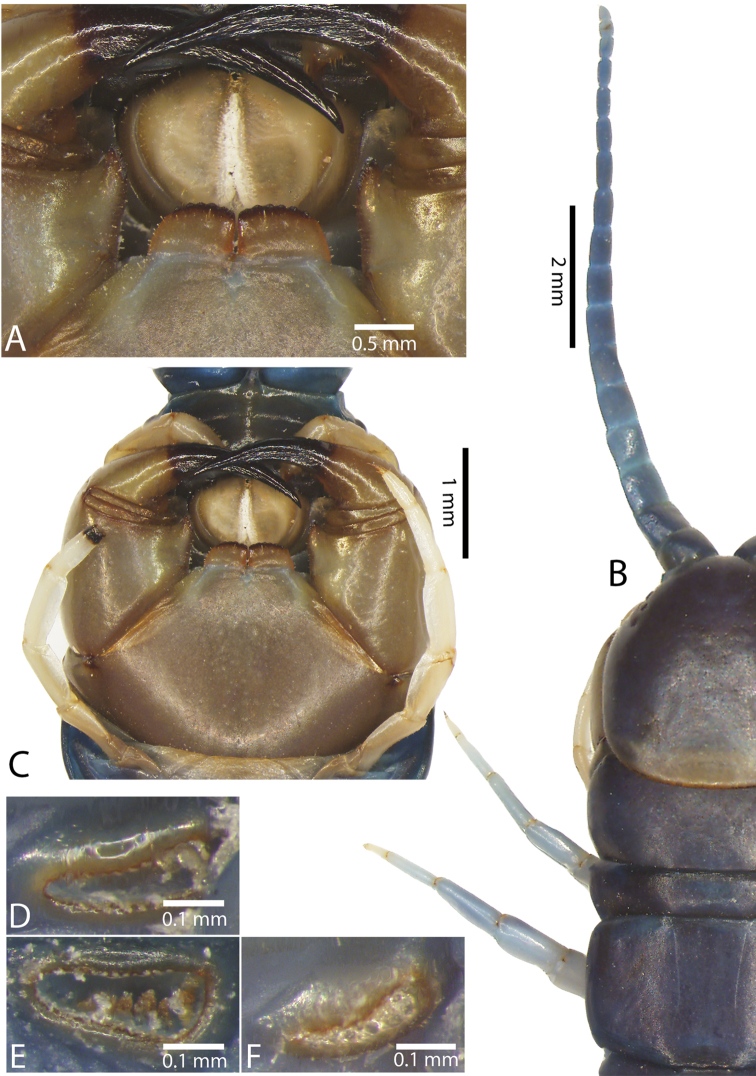
*Scolopendra calcarata* (CUMZ 00418): **A** Tooth-plates **B** Cephalic plate and trunk segments 1–3 **C** Forcipular segment **D–F** Spiracles 3, 5 and 8, respectively.

**Figure 31.**
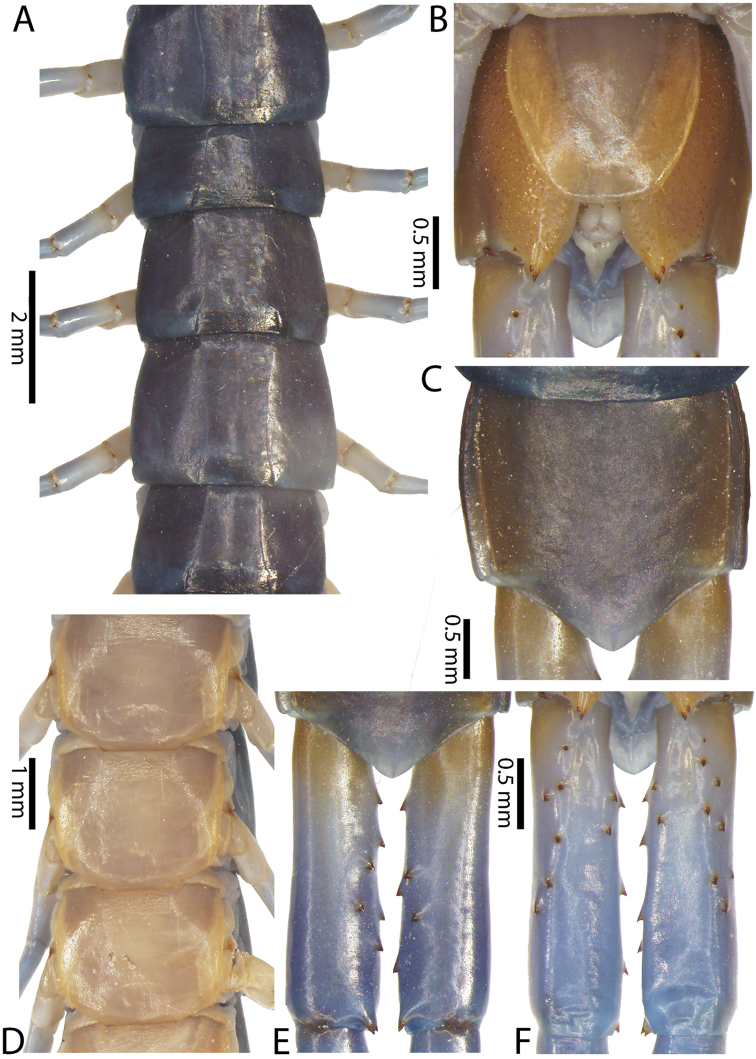
*Scolopendra calcarata* (CUMZ 00418): **A** Tergites 8–12 **B** Sternite of ultimate leg-bearing segment and coxopleura **C** Tergite of ultimate leg-bearing segment **D** Sternites 10–12 **E–F** Dorsal and ventral views of ultimate leg prefemora.

**Figure 32.**
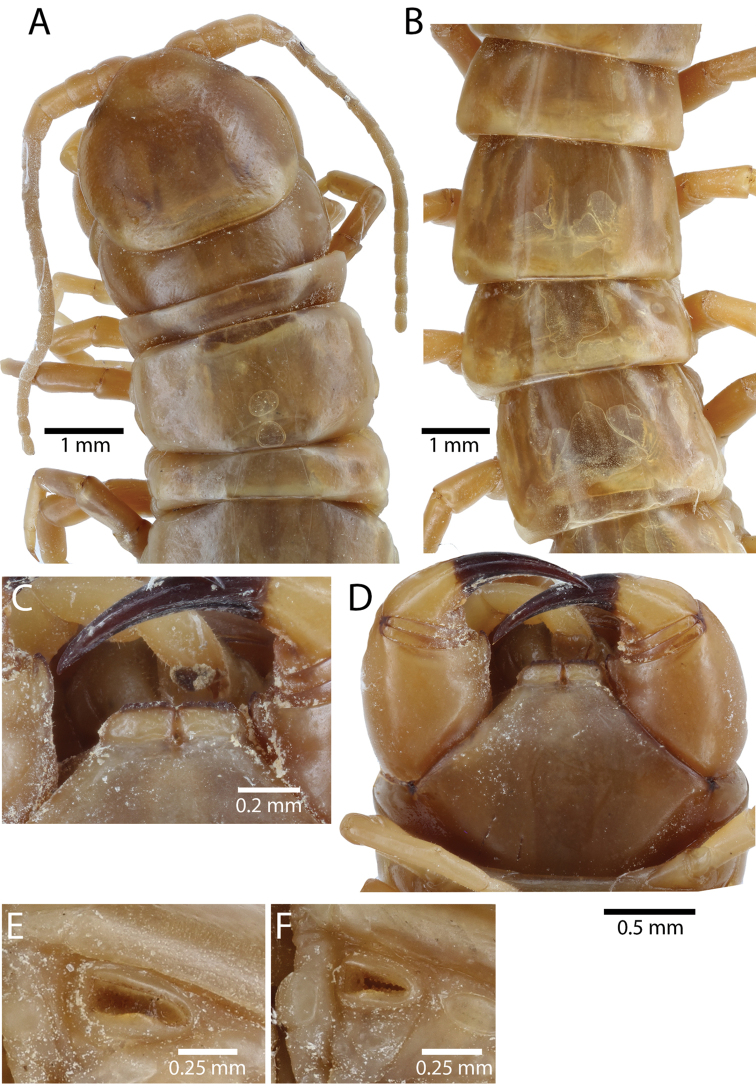
*Scolopendra calcarata* (Holotype NHRS–KASI 000000042): **A** Cephalic plate and trunk segments 1–5 **B** Tergites 8–11 **C** Tooth-plates **D** Forcipular segment **E–F** Spiracles 3 and 5, respectively.

**Figure 33.**
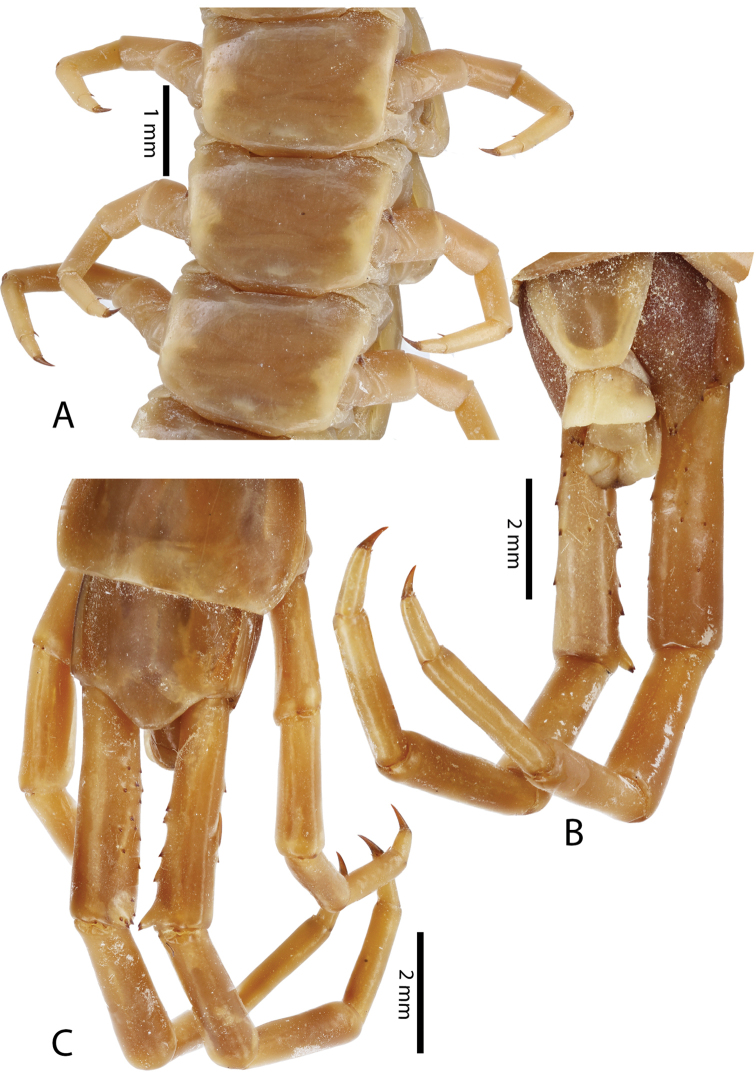
*Scolopendra calcarata* (Holotype NHRS–KASI 000000042): **A** Sternites 10–12 **B** Sternite of ultimate leg-bearing segment, coxopleura and ventral view of ultimate legs **C** Tergite of ultimate leg-bearing segment and dorsal view of legs 20 and ultimate legs.

##### Additional material.

**Thailand** — CUMZ 00417, one spm., Wat Mae Long, Mae Chaem, Chiang Mai (18°13'42.315"N, 98°26'23.003"E). CUMZ 00418, one spm., Lan Sang Waterfall, Mueang, Tak (16°46'36.861"N, 99°0'39.441"E). CUMZ 00312, one spm., Chong Kao Khat (Hellfire Pass), Kanchanaburi (14°22'47.6"N, 98°55'47.7"E).

##### Diagnosis.

17 antennal articles, 4–6 basal articles glabrous dorsally. Each tooth-plate with 5–8 teeth. Tergites 3–20 with paramedian sutures. Complete margination only on tergite of ultimate leg-bearing segment. Tergite of ultimate leg-bearing segment without depression or median suture. Incomplete paramedian sutures on sternites. Coxopleural process with 3–4 apical, 0–3 subapical and 0–1 lateral spines, without dorsal spine. Ultimate leg prefemora with 4–12 VL, 0–12 VM, 1–5 M, 2 DM, prefemoral process with 3–5 spines. One tarsal spur on legs 1–21.

##### Composite description.

Body length up to 5.3 cm. Blackish colouration on most of dorsal part of body. Cephalic plate dichromatic; anterior part of cephalic plate dark blue or black, posterior margin green yellowish (Fig. 30B). Antenna dark blue. Tergites dark blue or nearly blackish. All legs light blue, their basal part yellowish. Cephalic plate with median sulcus on anterior part. Posterior part of cephalic plate without paramedian sulci.

**Figure 34.**
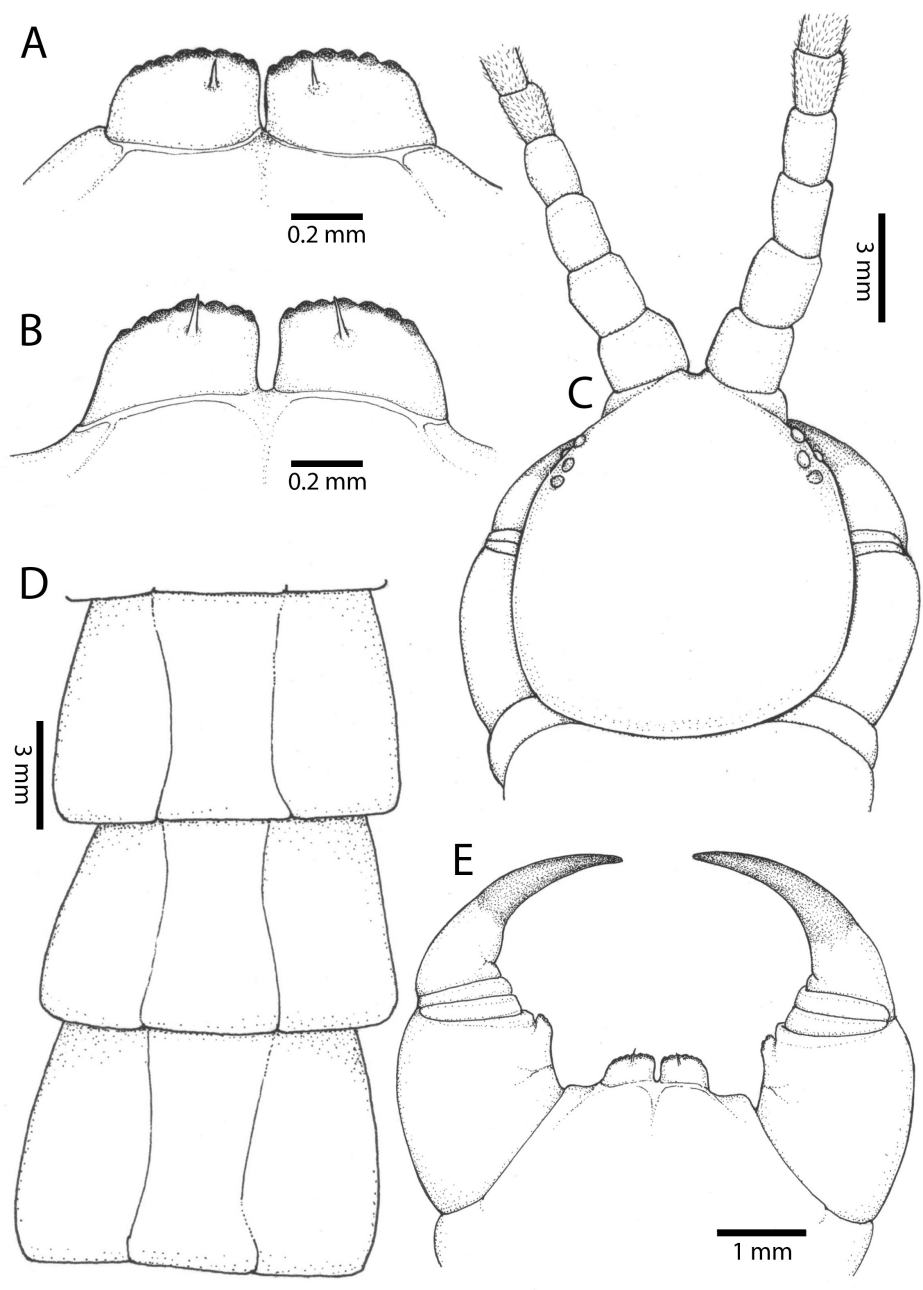
*Scolopendra calcarata* (CUMZ 00418): **A–B** Variation of teeth on tooth-plates **C** Cephalic plate and basal antennal articles **D** Tergites 9–11 **E** Forcipular segment.

Antenna usually with 17 articles, basal 4–6 subcylindrical, glabrous dorsally (Fig. 34C), 3.3 glabrous articles ventrally. Antenna reaching segments 3–4. Forcipular trochanteroprefemoral process with denticles in two groups, one apical and 2–3 inner. Tooth-plates with 5–8 teeth (Figs 30A, 34A–B). Tooth-plate with straight, transverse basal suture. Coxosternite smooth, without median suture (Figs 30C, 34E). Article 2 of second maxillary telopodite with spur.

**Figure 35.**
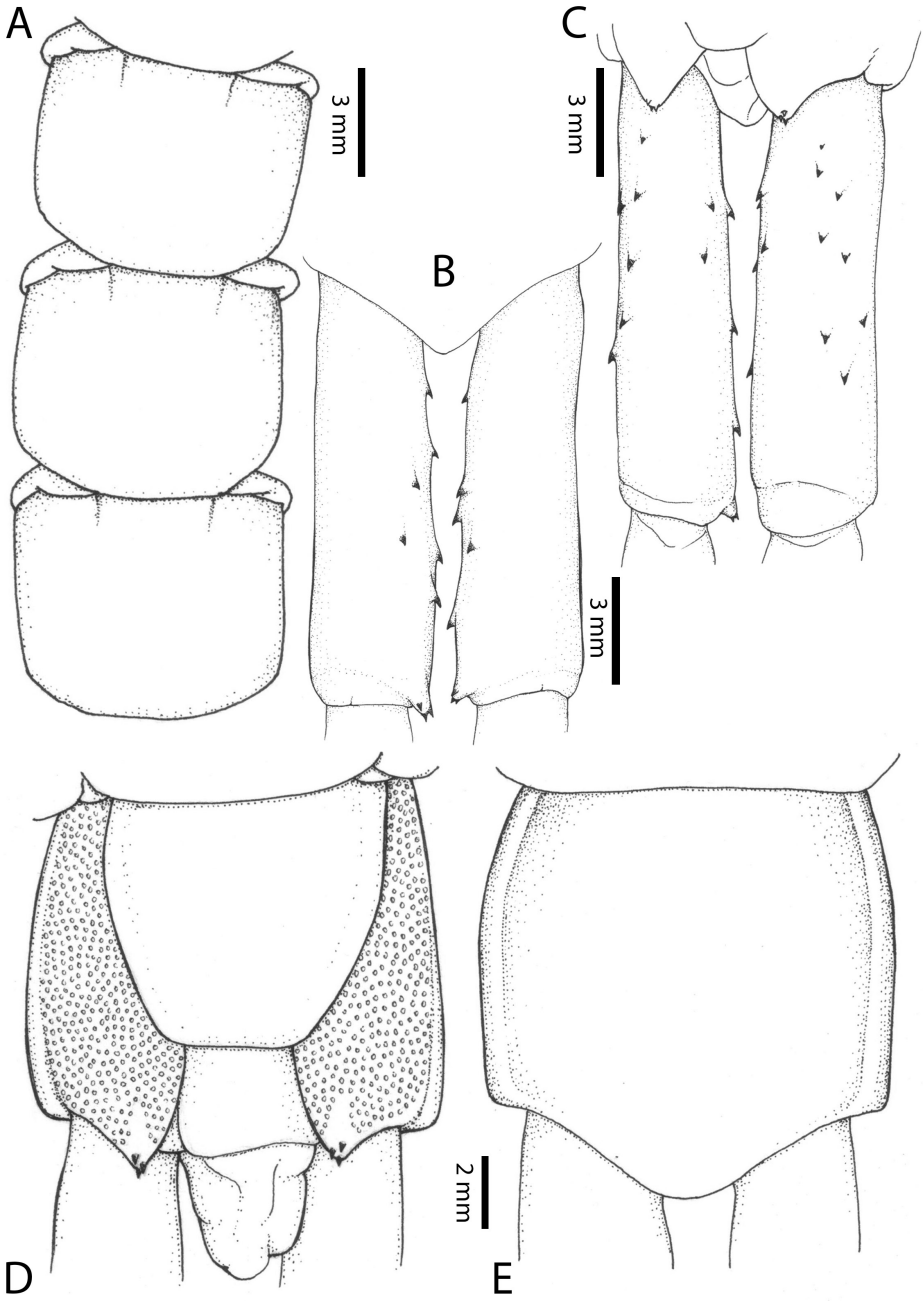
*Scolopendra calcarata* (CUMZ 00418): **A** Sternites 9–11 **B–C** Dorsal and ventral views, respectively, of ultimate leg prefemora **D** Sternite of ultimate leg-bearing segment and coxopleura **E** Tergite of ultimate leg-bearing segment.

Anterior margin of T1 underlying cephalic plate (Figs 30B, 34C). Complete paramedian sutures from TT3–4; tergite margination only on T21 (starting on T15 in holotype). Tergite surfaces (Figs 31A, 34D) smooth, without median sulci. Tergite of ultimate leg-bearing segment with curved, acute posterior margin (Figs 31C, 35E), lacking median furrow or depression; ratio of width: length of tergite of ultimate leg-bearing segment 0.87:1. Sternites (Figs 31D, 35A) with incomplete paramedian sutures occupying anterior 10–30%. Surface of sternites smooth, without depression. Sternite of ultimate leg-bearing segment (Figs 31B, 35D) with sides converging posteriorly. Pore-field on coxopleuron terminating well beneath margin of tergite of ultimate leg-bearing segment, dorsal margin of pore area elevated equally along its length.

Coxopleural process moderately long, with 3–4 apical, 0–3 subapical and 0–1 lateral spines, without dorsal spine. Pore-free area extending 50–75% length from distal part of coxopleural process to margin of sternite of ultimate leg-bearing segment (Figs 31B, 35D).

All legs with small setae, without tibial spur. One tarsal spur on legs 1–21. Ultimate legs moderately long and slender, with ratios of lengths of prefemur and femur 1.2:1, femur and tibia 1.2:1, tibia and tarsus 2 1.4:1.; tarsus 1 and tarsus 2 2.6:1. Prefemoral spines (Figs 31E–F, 35B–C): 4–7 VL, 0–3 VM, 1–5 M, 1–7 DM, prefemoral process with 2–5 spines.

Genital segments well developed, reaching longer than distance between posterior margin of sternite of ultimate leg-bearing segment and distal part of coxopleural process. Sternite of genital segment 1 round and convex posteriorly, with median suture (Fig. 25B). In male, sternite of genital segment 2 well developed. Tergites of genital segments with small setae.

##### Discussion.

This montane species was sometimes collected together with other scolopendrids such as species of *Otostigmus* and *Rhysida*. External phenotypic characters are similar to *Scolopendra pinguis* but the unique, diagnostic character that permits species identification is the presence of a tarsal spur on the ultimate legs, which is atypical for *Scolopendra*. However, this character has been reported in some individuals of a few other *Scolopendra* species in Southeast Asia, notably *Scolopendra subcrustalis* (see Kronmüller 2009, Lewis 2010b). We also recorded the occurrence of a tarsal spur on the ultimate leg in two juveniles of *Scolopendra subspinipes* from Yokohama, Japan (NHMW 758). For this reason, variation in tarsal spurs on legs needs to be used cautiously for justification of species boundaries when sample size is limited. However, *Scolopendra calcarata* is readily distinguished morphologically from *Scolopendra subspinipes* s.l. and *Scolopendra subcrustalis* by the number of glabrous antennal articles (four glabrous dorsally), only the tergite of the ultimate leg-bearing segment showing margination, sternites with incomplete paramedian sutures, and 4–5 apical and subapical spines on the coxopleural process. All specimens from Thailand and the description of Vietnamese populations by Schileyko (1995) consistently exhibited a tarsal spur on the ultimate legs. However, some characteristics seem to be variable between these two populations, such as a count of six glabrous antennal articles in Vietnam versus four in Thailand (four in the original description), the last 4–6 tergites marginated versus only the tergite of the ultimate leg-bearing segment (but in original description, margination starting from TT12 (13)), the sternite of the ultimate leg-bearing segment with a median depression versus its absence, and the arrangement of spines on the ultimate leg prefemur. With respect to the latter, Vietnamese populations exhibited 9–12 VL, 11–12 VM, 2–3 both M and DM, and 2 spines on the prefemoral process versus 4–7 VL, 0–3 VM, 1–2 M, 1–2 DM, and 2–4 spines on the prefemoral process in Thai populations.

Morphological similarity between *Scolopendra calcarata* and *Scolopendra pinguis* is indicated by several characteristics, including the number of antennal articles, the shape of teeth on the forcipular tooth-plates (these being in the form of minute denticles), and the number of spines on the coxopleural process. In addition, the habitat preferences of these two species resemble each other, both of them being found only in montane territory, and they also show similar dichromatic colouration patterns. There is no evidence from our survey that these two species are distributed sympatrically. These characters are consistent with the molecular phylogeny, which resolves these two species as sister taxa.

##### Distribution.

This species is quite rare in tropical mainland Southeast Asia, usually distributed along mountain ranges in the western territory of Thailand (Fig. 29). A few specimens were found in the eastern plateau. Schileyko (1995, 2007) also reported material from northern Vietnam, and Zhang (1992) recorded *Scolopendra calcarata* in southern China. We combine all records of this species from previous literature and our new collections to provide and updated distribution as follows: **Southeast Asia**: Thailand (Kanchanaburi, Tak and Chiang Mai) and Vietnam (fide Schileyko (2007): Mai Chau, Tam Dao National Park, Cat Ba National Park and Ba Vi National Park). **East Asia**: South China.

#### Scolopendra japonica

Taxon classificationAnimaliaScolopendromorphaScolopendridae

Koch, 1878

Figs 7D , 9C , 29 , 36 , 37 , 38 , 39 , 40 , 41

Scolopendra japonica Koch, 1878: 790. Daday 1889: 149. Haase 1887: 48, pl. 3, fig. 48. Takakuwa 1942: 41, 1943: 171, 1947: 938. Kronmüller 2012: 24, figs 3D, 4B.
Scolopendra subspinipes japonica – Kraepelin 1903: 263. Attems 1909: 10, 13, 1914: 107, 1930b: 30. Shinohara 1949: 81, 1961: 75. Takashima 1949: 11, 1952: 4. Chamberlin and Wang 1952: 180. Miyosi 1955: 151. Wang 1955: 16. Takano 2001: 211. Lewis 2010b: 114.
Otostigmus politoides Attems, 1953: 147. Lewis 2004: 32, Figs 14–17. Kronmüller 2012: 25.
Otostigmus puncticeps Attems, 1953: 146, figs 16–17. Lewis 2004: 30, figs 8–13. Kronmüller 2012: 25.

##### Type locality.

Japan.

##### Material.

**Syntype**: NHMW Inv. No. 5368, one female from Japan, leg. Roletz, don. Latzel, 1919, det. Attems C (Figs 38, 39). **Probable syntypes**: NHMW Inv. No. 762, seven spms., Japan, leg. Roletz, red (type) label in bottle.

**Figure 36.**
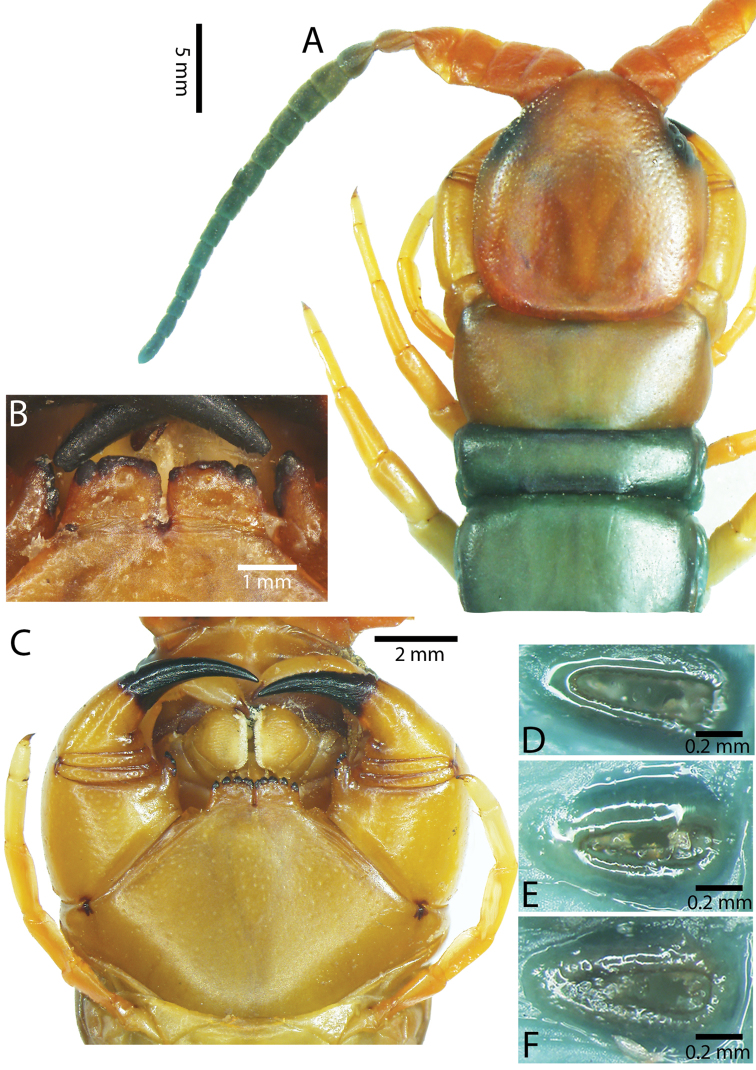
*Scolopendra japonica* (CUMZ 00297, 00298): **A** Cephalic plate and trunk segments 1–3 **B** Tooth-plates **C** Forcipular segment **D–F** Spiracles 3, 5 and 8, respectively.

**Figure 37.**
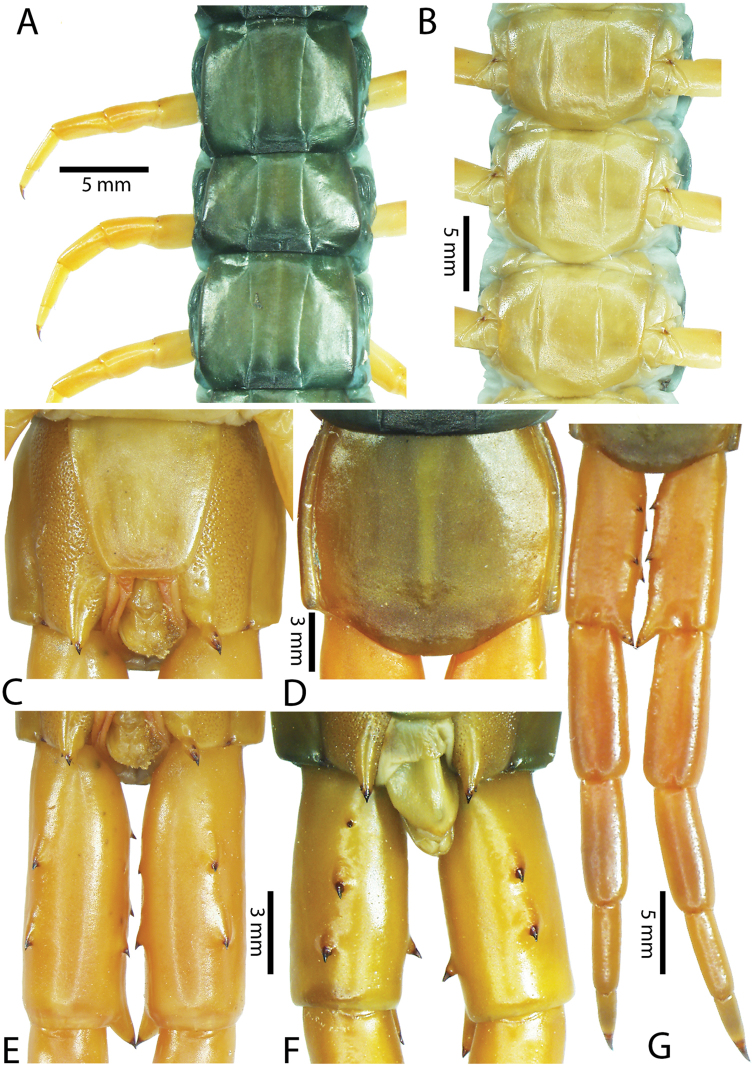
*Scolopendra japonica* (CUMZ 00297, 00298): **A** Tergites 9–11 **B** Sternites 9–11 **C** Sternite of ultimate leg-bearing-segment and coxopleura **D** Tergite of ultimate leg-bearing segment **E** and **F** Variation in ventral spines on ultimate leg prefemora **G** Dorsal view of ultimate legs.

**Figure 38.**
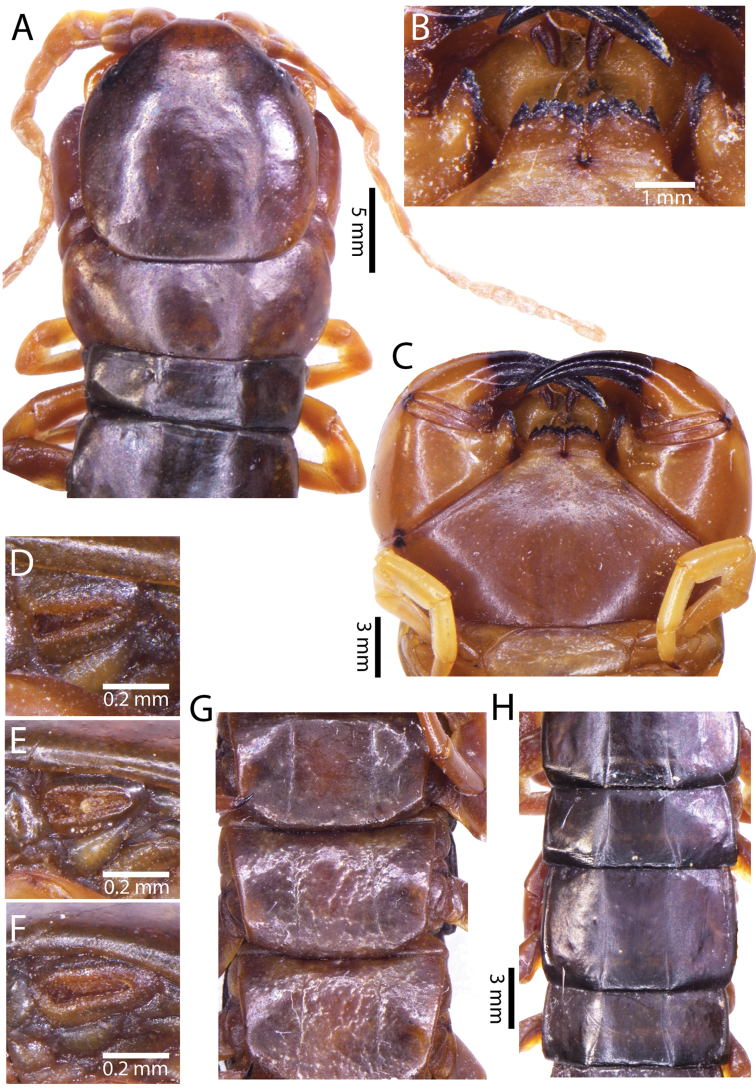
*Scolopendra japonica* (Syntype NHMW 5368): **A** Cephalic plate and trunk segments 1–3 **B** Tooth-plates **C** Forcipular segment **D–F** Spiracles 3, 5 and 8, respectively **G** Sternites 9–11**H** Tergites 8–11.

**Figure 39.**
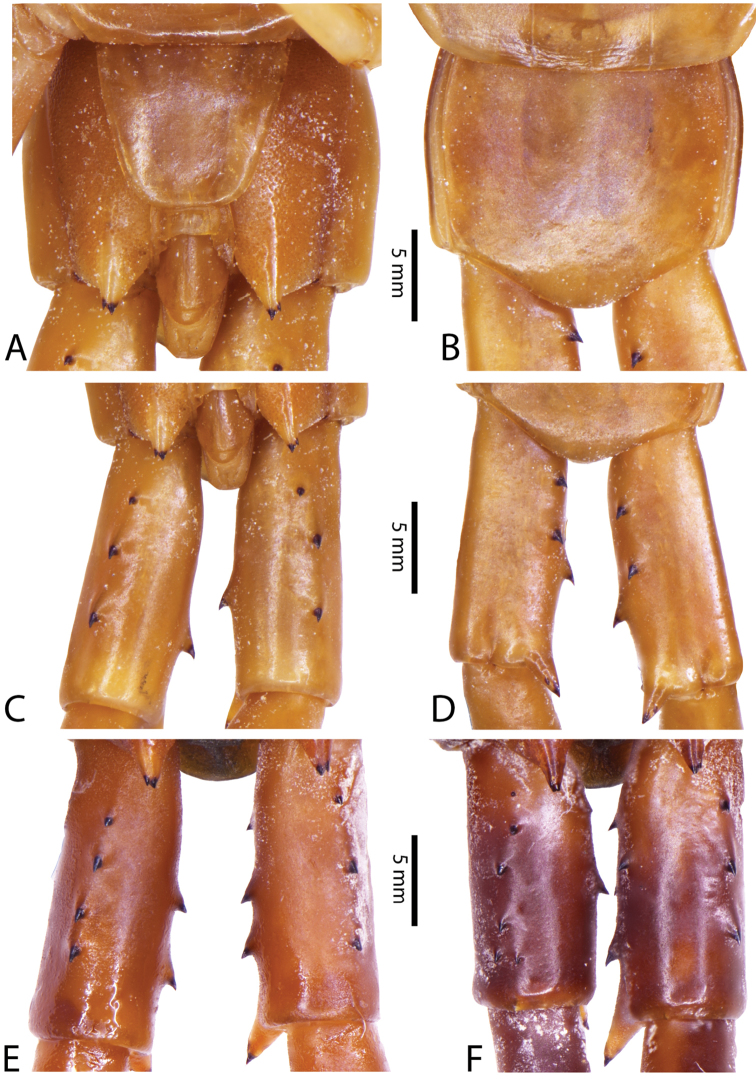
*Scolopendra japonica* (Syntype NHMW 5368): **A** Sternite of ultimate leg-bearing segment and coxopleura **B** Tergite of ultimate leg-bearing segment **C–D** Ventral and dorsal views of ultimate leg prefemora **E–F** Variation in ventral spines of ultimate leg prefemora.

##### Additional material.

**Laos** — CUMZ 00297.1-3, two spms., Phu Fah Mountain, Phongsaly, Laos (21°41'19.6"N, 102°06'30.4"E). CUMZ 00298.1-5, five spms., Plain of Jar, Xiang Khouang, Laos (19°25'51.5"N, 103°09'10.4"E).

**Japan** — CUMZ 00319, one spm., Shinshu University, Matsumoto, Japan (36°13'22.4"N, 137°54'35.0"E). NHMUK 1893.1.15.3, one spm., Tokyo. NHMUK 1912.12.12.914, two spms., Izu Peninsula. NHMUK 1937.9.9.59, one spm., Japan, det. K.W. Verhoeff. NHMW Inv. No. 758, six spms., Yokohama. NHMW Inv. No. 755, seven spms., Japan. NHMW Inv. No. 757, one spm., Okayama, leg. H. Sauler. NHMW Inv. No. 759, one spm., Kioto [Kyoto] with anther label “344”. NHMW Inv. No. 760, two spms., Kioto [Kyoto], leg. H. Sauler. NHMW Inv. No. 756, two spms., Kanagava [Kanagawa], leg. H. Sauler, 17/11/1905, bottle with labels, “Hans Lauter 4122, Kanagawa 17/11/1905 Jan Haus” and “Hans Lauter 3236, Kanagawa 25/6/1905 Sichen”.

**Indonesia** — NHMUK 1882.62, one spm., Sumatra.

**China** — NHMUK, one spm., Loc. 273, Peak of Flat No. 2, Hong Kong. NHMUK 1904.7.23.5–8, two spms., Yunnan-Fu [Kunming], South China.

**Undetermined** — NHMW Inv. No. 761, 17 spms., Kuile? [Possibly referring to Korea?].

**Diagnosis.** 17–19 antennal articles, 6 basal articles glabrous dorsally. Each tooth-plate with 4–6 teeth. Tergites (3)4–20 with paramedian sutures. Complete margination from TT(10)12–21. Tergite of ultimate leg-bearing segment without depression or suture. Complete paramedian sutures on sternites 2–20. Coxopleural process with 3 apical spines. Ultimate leg prefemora with 2–3 VL, 1–2 M, 1–3 DM and prefemoral process with 0–5 spines. One tarsal spur on legs 1–19(20).

##### Composite description.

Body length up to 12.9 cm. Two colour morphs; morph 1 with antenna and legs 1–20 yellowish, morph 2 with antenna and legs 1–20 reddish. All tergites greenish brown. Cephalic plate with median sulcus. Paramedian sulci or sutures absent on posterior part.

**Figure 40.**
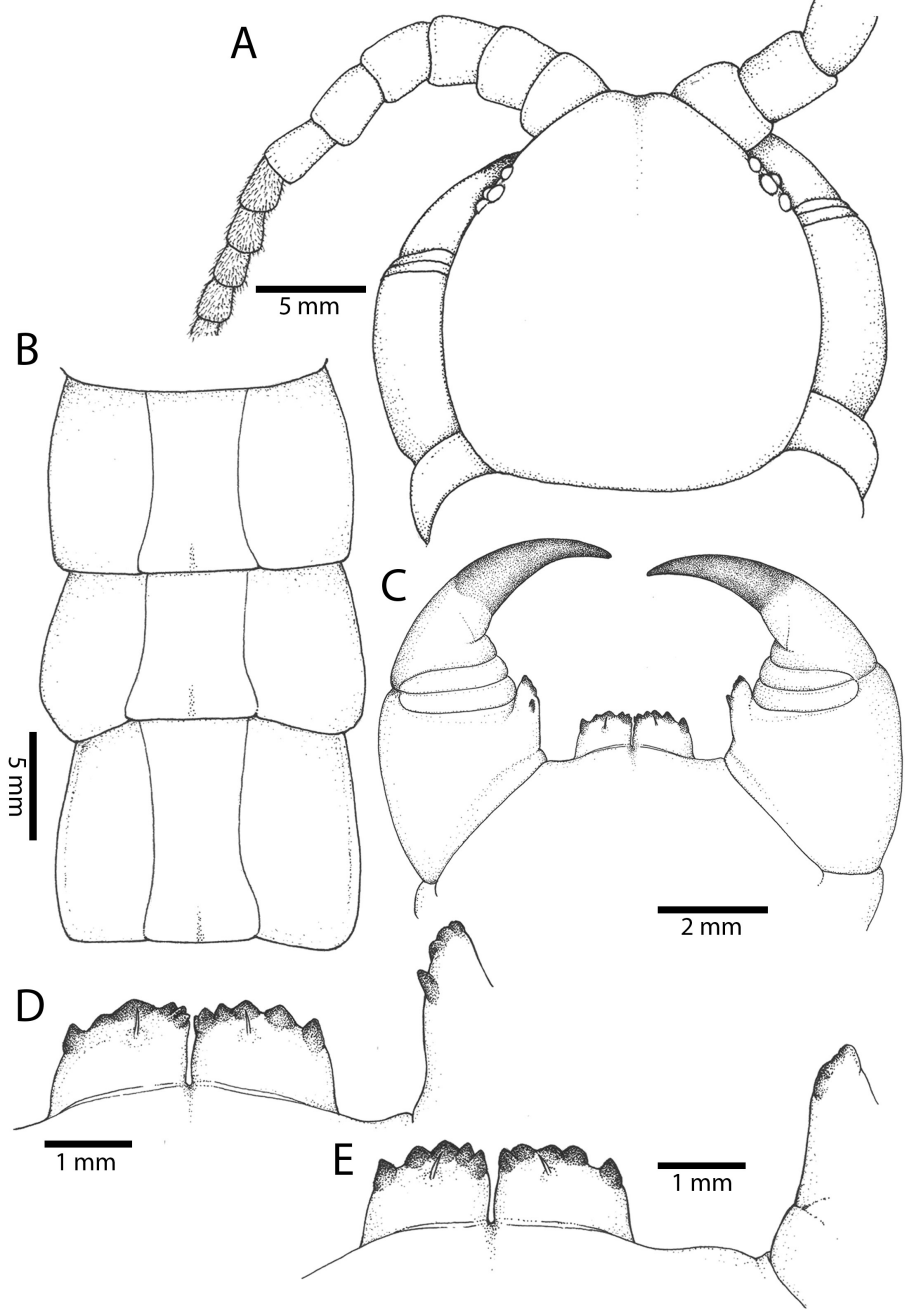
*Scolopendra japonica* (CUMZ 00297): **A** Cephalic plate and basal antennal articles **B** Tergites 9–11 **C** Forcipular segment **D–E** Variation in teeth on tooth-plates and trochanteroprefemural process.

Antenna usually with 18 articles (atypically with 17 or 19), basal 6 subcylindrical and glabrous dorsally (Fig. 40A), the rest spherical. Antennae reach to tergite 2 (Fig. 36A). Forcipular trochanteroprefemoral process bearing denticles in two groups, one apical and 2–3 inner (Fig. 36B–C). Tooth-plates wider than long, with 4–6 teeth (Figs 36B, 40D–E). Tooth-plate with straight, transverse basal suture. Coxosternite smooth without median suture (Figs 36C, 40C). Article 2 of second maxillary telopodite with spur.

**Figure 41.**
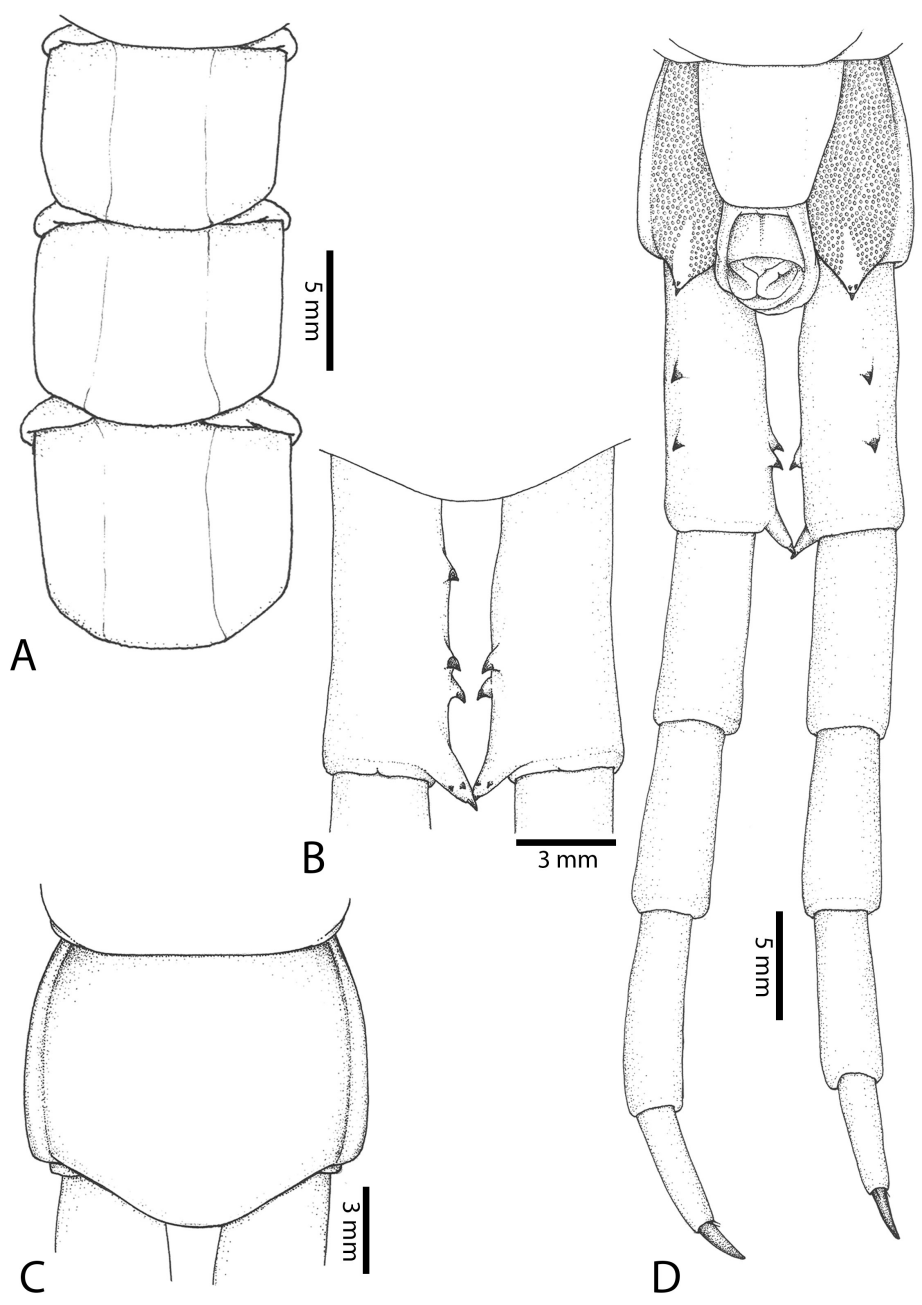
*Scolopendra japonica* (CUMZ 00297, 00298): **A** Sternites 9–11 **B** Dorsal view of ultimate leg prefemora **C** Tergite of ultimate leg-bearing segment **D** Sternite of ultimate leg-bearing segment, coxopleura and ultimate legs.

Anterior margin of T1 underlying cephalic plate (Fig. 36A). Complete paramedian sutures from TT3–4; margination typically starting on T10. Tergite surface (Figs 37A, 40B and 41A) with median posterior sulci in TT10–20. Tergite of ultimate leg-bearing segment (Figs 37D, 41B) curved posteriorly, without median furrow or depression; ratio of width: length of tergite of ultimate leg-bearing segment 0.82:1. Sternites (Figs 37B, 41C) with complete paramedian sutures, without depression or pit on surface. Sternite of ultimate leg-bearing segment (Fig. 37C) with sides converging posteriorly, surface with obscure depression on median part. Lateral part of coxopleuron with pore-field terminating beneath margin of tergite of ultimate leg-bearing segment.

Coxopleural process moderately long, usually with three apical spines (Fig. 39A); pore-free area extending 70–90% length from distal part of coxopleural process to margin of sternite of ultimate leg-bearing segment.

All legs without setae and tibial spur. One tarsal spur on legs 1–19 (on leg 20 in one spm.). Ultimate legs: moderately thick and long (Figs 37G, 41E), with ratios of lengths of prefemur and femur 1.1:1, femur and tibia 1.3:1, tibia and tarsus 2 1.4:1; tarsus 1 and tarsus 2 1.8:1. Prefemoral spines (Figs 37E–F, 41D–E): 2–3(4) VL, 1–2 VM, 1–3 DM, prefemoral process with 0–5 spines.

Genital segments well developed, reaching longer than distance between posterior margin of sternite of ultimate leg-bearing segment and distal part of coxopleural process. Sternite of genital segment 1 round and convex posteriorly, with median suture (Fig. 7D). Tergites of genital segment without small setae. Gonopod present in male.

##### Discussion.

The validity of *Scolopendra japonica* at the species level was defended by Kronmüller (2012) from morphological surveys of former subspecies of *Scolopendra subspinipes*. Diagnostic characters of this species by comparison to the other former subspecies are the number of apical spines on the coxopleural process (*Scolopendra subspinipes* s.str. having two apical spines versus *Scolopendra japonica* having three spines, including a subapical spine), and the number of ventral spines on the prefemur of the ultimate legs. With respect to the latter, *Scolopendra japonica* has three spines whereas *Scolopendra subspinipes* s.str. has only two, although in this study an asymmetrical number of spines in *Scolopendra japonica* was also found in syntypes (Fig. 39C–F). Four specimens among the *japonica* syntypes have been recorded as presenting only two or three ventral spines on the prefemur of the ultimate leg either on only one side or on both (six spines is the observed maximum). This variability might represent ontogenetic variation more so than geographical variation. Chao (2008) cited the lack of a tarsal spur on leg 20 as an additional diagnostic character for this species but from our study one specimen of *Scolopendra japonica* from the Izu Peninsula, Japan (NHMUK 1912.12.12.914), also exhibits a tarsal spur on leg 20. As such, the occurrence of a spur on leg 20 and number of ventral spines on the ultimate leg prefemur are not completely reliable for diagnosing *Scolopendra japonica*.

The sympatric distribution of this species and former subspecies of *Scolopendra subspinipes* as well as other Asian temperate *Scolopendra* complicates morphological delimitation of species boundaries except using the three phenotypic characters discussed above. In this paper, we compared taxonomic characters based on collections in the NHMUK and NHMW (Table 8). Additional characters of these two species that might be useful for species identification are the proportions of the ultimate leg podomeres and the number of spines on the prefemoral process on the ultimate leg. In *Scolopendra subspinipes* s.str., two spines are usually present on the prefemoral process whereas in *Scolopendra japonica* there are typically three. The length of the antenna also permits a distinction berween these two species; the antenna extends backwards only as far as TT2–3 in *Scolopendra japonica* whereas it can reach to TT4–5 in *Scolopendra subspinipes* (Figs 10A, 14B and 36A). Moreover, molecular analysis of three combined genes (COI, 16S and 28S) indicated a genetic distinction between *Scolopendra japonica* and *Scolopendra subspinipes* (Fig. 1), and the validity of these species has been corroborated herein. Ratios of ultimate leg podomeres have been used as diagnostic characters of some putative species of *Scolopendra* in Asia, such as *Scolopendra negrocapitis* Zhang and Wang, 1999 from Jingshan (northeast coastal area of China). The authors mentioned the close similarity between that species and *Scolopendra japonica* but the Chinese species can be distinguished from the latter only by the width:length ratio of the ultimate leg prefemur, which is twice as long as broad. The lack of further information from fresh material from the type locality and molecular data from *Scolopendra negrocapitis* renders the status of these two closely related species questionable.

**Table 8.** Morphological comparison of *Scolopendra japonica* populations in the present study and the related species *Scolopendra cingulata*.

Characters	*Scolopendra japonica*			*Scolopendra cingulata*
	Japan (Syntypes)	Japan-China	Laos	
Number of antennal articles	17–19	17–19	12–18	17–22
Number of glabrous articles	6	6	6	6
Teeth on tooth-plate	5+5	4+5, 5+5	6+6, 5+5	4+4,5+5
First tergite with complete paramedian sutures	3–4	3–4	4	2–3
First tergite with margination	10–13	11–15	12	7–12
Tergite surface	smooth	smooth	short median furrow on posterior part of TT7–19	smooth
Median furrow on tergite of ULBS	absent	absent	absent	absent
Paramedian sutures on sternites	complete	complete	complete	complete
Sternite of ultimate leg-bearing segment	sides converging posteriorly	sides converging posteriorly	sides converging posteriorly	sides converging posteriorly
Spines on coxopleural process	AP: 0–3 SAP: 0–1	AP: 0–3 SAP: 0–1	AP: 3	AP+SAP: 1–5
Spine formula on prefemora of ultimate legs	VL: 2–3 M: 1–2 DM: 1–3 SP: 0–3	VL: 2–3 M: 1–2 DM: 1–2 SP: 1–5	VL: 2–3 M: 1 DM: 2 SP: 3–4	VL: 1–2 M+DM: 4–8 SP: 1–11
Legs with one tarsal spur	1–19 (20)	1–19 (1–20)	1–19	19

In the current phylogenetic framework of *Scolopendra*, *Scolopendra japonica* is resolved in the same clade as *Scolopendra cingulata* Latreille, 1829. The two species are morphologically similar despite their markedly disjunct distributions, i.e., *Scolopendra cingulata* in the Mediterranean versus *Scolopendra japonica* in East Asia (Table 8). However, exploration of micro-refugia of populations of *Scolopendra cingulata* during glacial maxima in Europe (Simaiakis et al. 2012, Oeyen et al. 2014) and a record of *Scolopendra japonica* in the northern part of Laos could indicate that these two species may be more widespread than previously recognised. However, distributional data for *Scolopendra japonica* are patchy due to incomplete faunistic surveys in several parts in Asia. For these reasons, the relationship between these two species warrants further scrutiny in both morphological and molecular studies.

##### Distribution.

Probably distributed throughout the temperate zone of East Asia including mainland and insular territory (Fig. 29). The distribution range is likely to be sympatric with several Asian species (*Scolopendra subspinipes*, *Scolopendra multidens*, *Scolopendra dawydoffi* and *Scolopendra dehaani*). Previous study on the subspecies complex of *Scolopendra subspinipes* indicated that *Scolopendra japonica* might occur in northern Vietnam (Tonkin) and Cambodia, based on the type localities of “*Otostigmus politoides* Attems, 1953” and “*Otostigmus puncticeps* Attems, 1938” (= *Scolopendra subspinipes* fide Lewis 2004), the types of both of which are adolescent stages (Kronmüller 2012) that are compatible with *Scolopendra japonica*. The current distribution of this species gathered from previous literature and this study is as follows: **Southeast Asia**: Vietnam (Chapa, Tonkin [probably referring to Sa Pa, Lao Cai Province, northern Vietnam]), Laos (Phongsaly and Xieng Khuang), Cambodia (Ream, Koh Kong Island and Sre Umbell [Sre Ambel]), Indonesia (Sumatra). **East Asia**: Japan (Yokohama, Enoshima, Murayama, Tokyo, Matsumoto, Kanakawa, Sendai, Kii, Hachijo island and Izu Peninsula), Taiwan and China (Hong Kong, Yunnan-Fu [Kunming] and Ningbo).

#### Scolopendra pinguis

Taxon classificationAnimaliaScolopendromorphaScolopendridae

Pocock, 1891

Figs 7E–F , 25C , 29 , 42A–B , 43 , 44 , 45 , 46 , 47

Scolopendra pinguis Pocock, 1891b: 411, 1894: 312, pl. 19, fig. 4. Kraepelin 1903: 249. Attems 1907: 80, 1914a: 106, 1930b: 27. Lewis 2010b: 109.

##### Type locality.

1,000–2,000 ft., Carin Mountain, Cheba District, Burma [Kayah-Karen Mountains, Myanmar].

##### Material.

**Type material.** This species was described based on one specimen and the holotype was probably destroyed. It was collected during a field expedition to Burma (Myanmar) by Leonardo Fea, the assistant zoologist at the Museo Civico di Storia Naturale di Genova, Genova, Italy. In 1891, Pocock published on the myriapods of Burma based on Fea and Oates’s collections. Subsequently most of Oates’s collection was deposited in the NHMUK while Fea’s collection was sent back to Genova. The holotype of *Scolopendra pinguis* was explicitly identified as part of Fea’s collection (Pocock 1891: 411). In 1970, the basement of the museum in Genova was flooded and parts of the collection were irreparably damaged. The holotype of *Scolopendra pinguis* cannot presently be found (M. Tavano, written comm., November 2015) and is presumed to have been lost during that flood.

##### Additional material.

**Thailand** — CUMZ 00314, one spm., Phamone Cave, Pangmapha, Maehongson (19°30'01.6"N, 98°16'43.5"E). ZMUC 00101107, one spm., Siribhum Waterfall, Chomthong, Chiang Mai (18°32'49"N, 98°30'57"E), 1315 m, leg. C. Sutcharit, 13/10/2009. ZMUC, four specimens, 0–1.400 m, Doi Suthep National Park, leg. Bergit Degerbol, specimen Nos. 1841, 1828, 694 Loc. 3 and 2026. CUMZ 00313, one spm., Ban Pang Pan, Maetaeng, Chiang Mai (19°12'17.4"N, 98°40'00.7"E).CUMZ 00305, one spm., Phusang Waterfall, Phayao (19°37'10.2"N, 100°21'54.7"E). CUMZ 00307, one spm., Hui Nam-Un, Wiangkhum, Nan (18°30'22.8"N, 100°31'49.1"E). CUMZ 00311, one spm., Tat Ton Waterfall, Chaiyaphum (16°01'05.2"N, 102°01'24.4"E). CUMZ 00303, one spm., Wat Tham Lijia, Sangkhlaburi, Kanchanaburi (15°04'12.8"N, 98°33'56.4"E).

**Laos** — CUMZ 00304, one spm., Wiang Thong Hot Spring, Mueang Ieam, Houaphanh (20°04'45.2"N, 103°44'33.3"E). CUMZ 00310, two spms., Kra Cham Waterfall, Luang Prabang (19°32'27.3"N, 101°59'02.3"E). CUMZ 00306, one spm., Ban Na-Ton, Muang Khun, Xieng Khouang (17°52'31.4"N, 104°51'44.7"E). CUMZ 00309, one spm., Kao Rao Cave, Bo Kaeow (20°41'56.6"N, 101°05'46.8"E).

##### Diagnosis.

17 antennal articles, 3–4 basal articles glabrous dorsally. Each tooth-plate with 6 teeth. Tergites 3–20 with paramedian sutures. Complete tergite margination from TT16 (18)–21. Tergite of ultimate leg-bearing segment without depression or suture. Paramedian sutures on anterior 10–30% of sternites. Coxopleural process with 3–7 apical + subapical, 1–2 lateral and 0–1 dorsal spines. Ultimate leg prefemora with 6–12 VL, 1–12 VM, 2–3 M, 3–4 DM and prefemoral process with 3–4 spines. One tarsal spur on legs 1–19(20).

##### Composite description.

Body length up to 85 mm. Darkish blue colouration on entire body. Cephalic plate dichromatic in some populations. Tergites dark blue or nearly black. Cephalic plate with small punctae on anterior part, median sulcus present. Posterior part of cephalic plate without paramedian sulci.

Antenna usually with 17 articles, basal 3–4 subcylindrical and glabrous dorsally (Fig. 46A), 5–5.5 articles glabrous ventrally. Antennae reach segment 4. Forcipular trochanteroprefemoral process bearing denticles in two groups, one apical and 2–3 inner (Fig. 46D). Tooth-plates wider than long or nearly equivalent, with 6 teeth (Fig. 43C). Tooth-plate with straight, transverse basal suture. Coxosternite smooth, without median suture (Figs 44B, 44D and 46B). Article 2 of second maxillary telopodite with spur.

**Figure 42.**
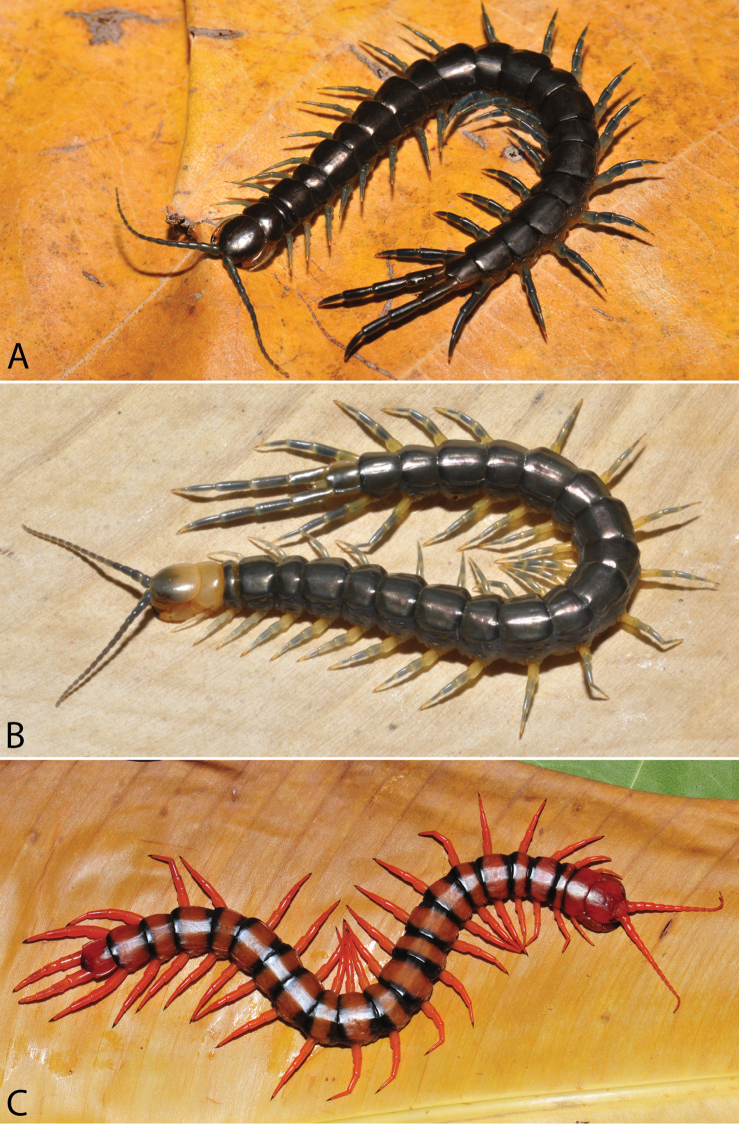
Habitus photographs of *Scolopendra* species: **A** *Scolopendra pinguis* (Colour morph 1A: CUMZ 00309) **B** *Scolopendra pinguis* (Colour morph 2B) **C** *Scolopendra dawydoffi* (CUMZ 00272).

**Figure 43.**
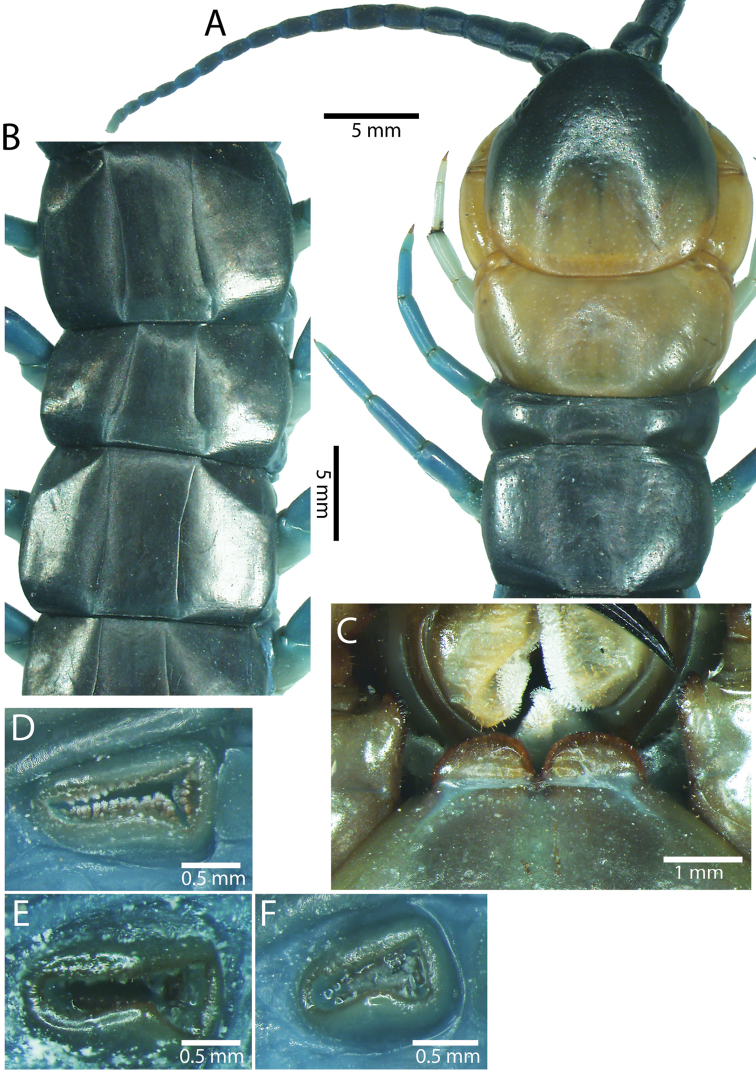
*Scolopendra pinguis* (CUMZ 00306, 00314): **A** Cephalic plate and trunk segments 1–3 (Colour morph 2) **B** Tergites 9–11 **C** Tooth-plates **D–F** Spiracles 3, 5 and 8, respectively.

**Figure 44.**
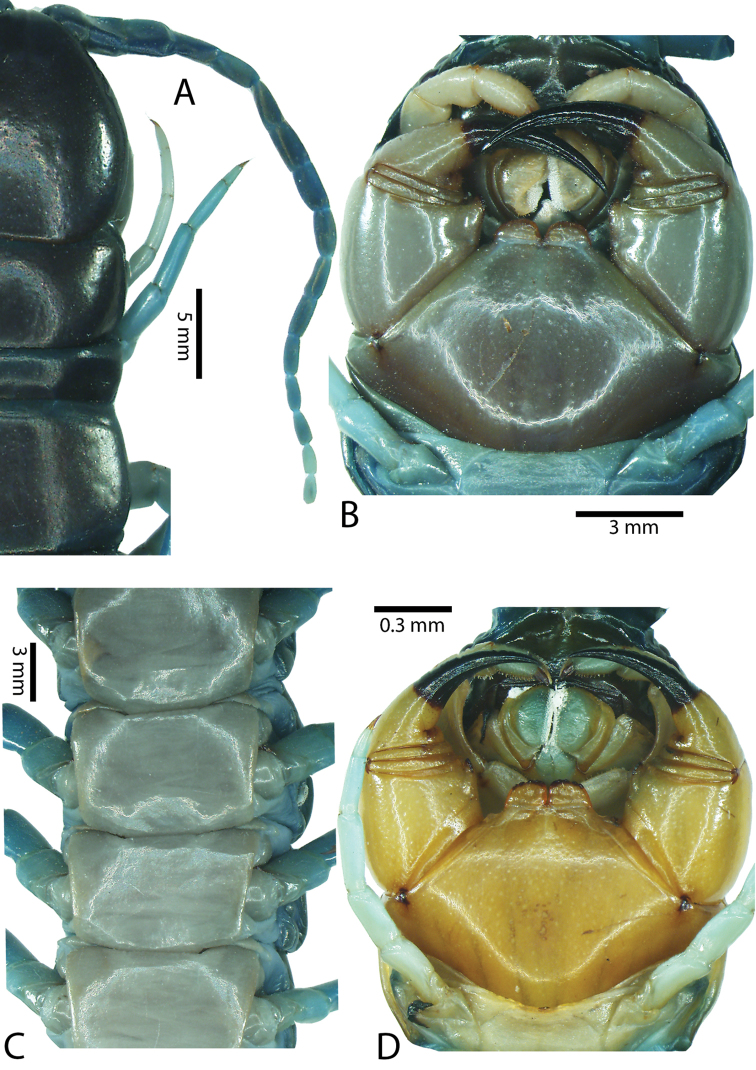
*Scolopendra pinguis* (CUMZ 00306, 00314): **A** Cephalic plate and trunk segments 1–3 (Colour morph 1) **B** Forcipular coxosternite (Colour morph 1A and 1B) **C** Sternites 9–12 **D** Forcipular coxosternite (Colour morph 2).

**Figure 45.**
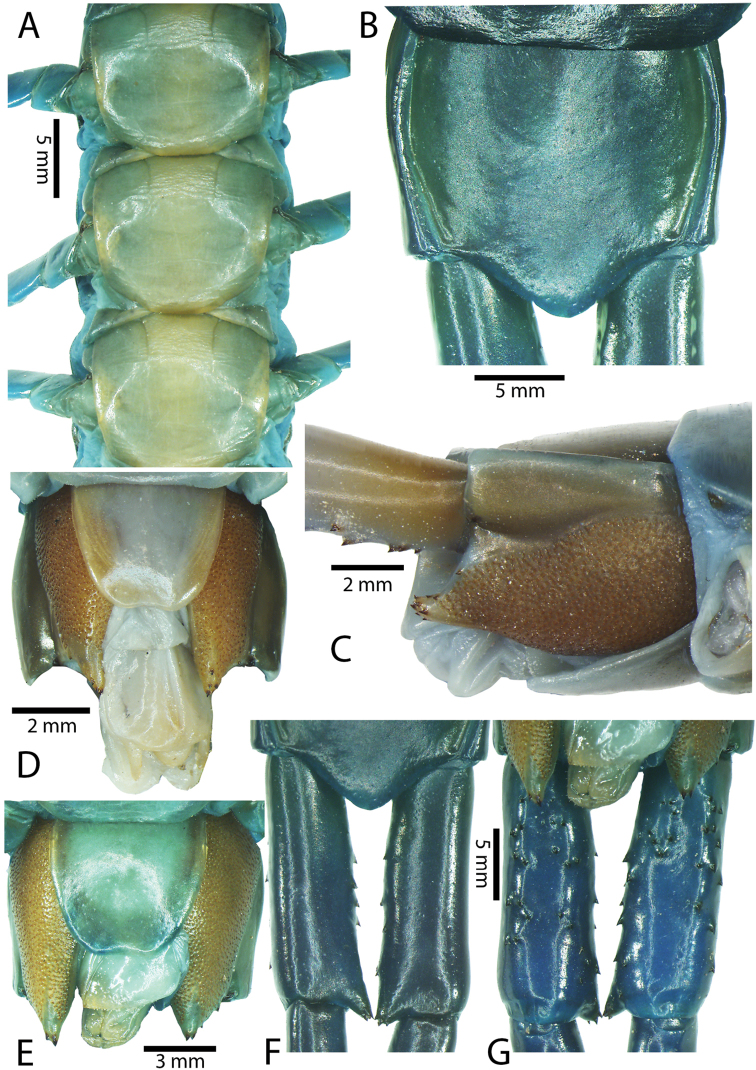
*Scolopendra pinguis* (CUMZ 00306, 00314): **A** Sternites 9–11 (Colour morph 2) **B** Tergite of ultimate leg-bearing segment **C** Lateral view of coxopleuron **D–E** Sternite of ultimate leg-bearing segment and coxopleura in male and female, respectively **F–G** Dorsal and ventral views, respectively, of ultimate leg prefemora.

**Figure 46.**
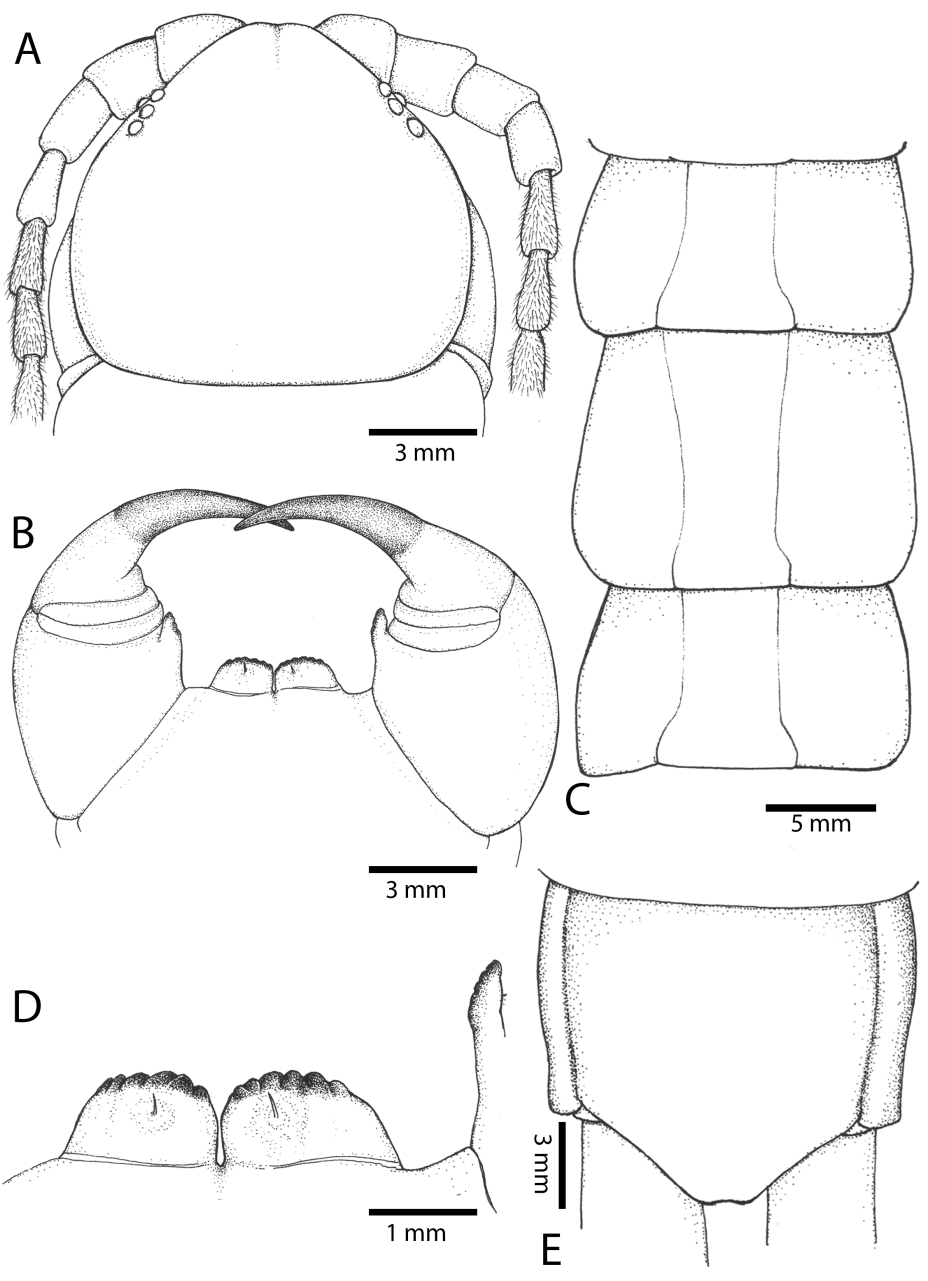
*Scolopendra pinguis* (CUMZ 00306, 00314): **A** Cephalic plate and basal antennal articles **B** Forcipular segment **C** Tergites 9–11 **D** Teeth on tooth-plates and trochanteroprefemoral process **E** Tergite of ultimate leg-bearing segment.

**Figure 47.**
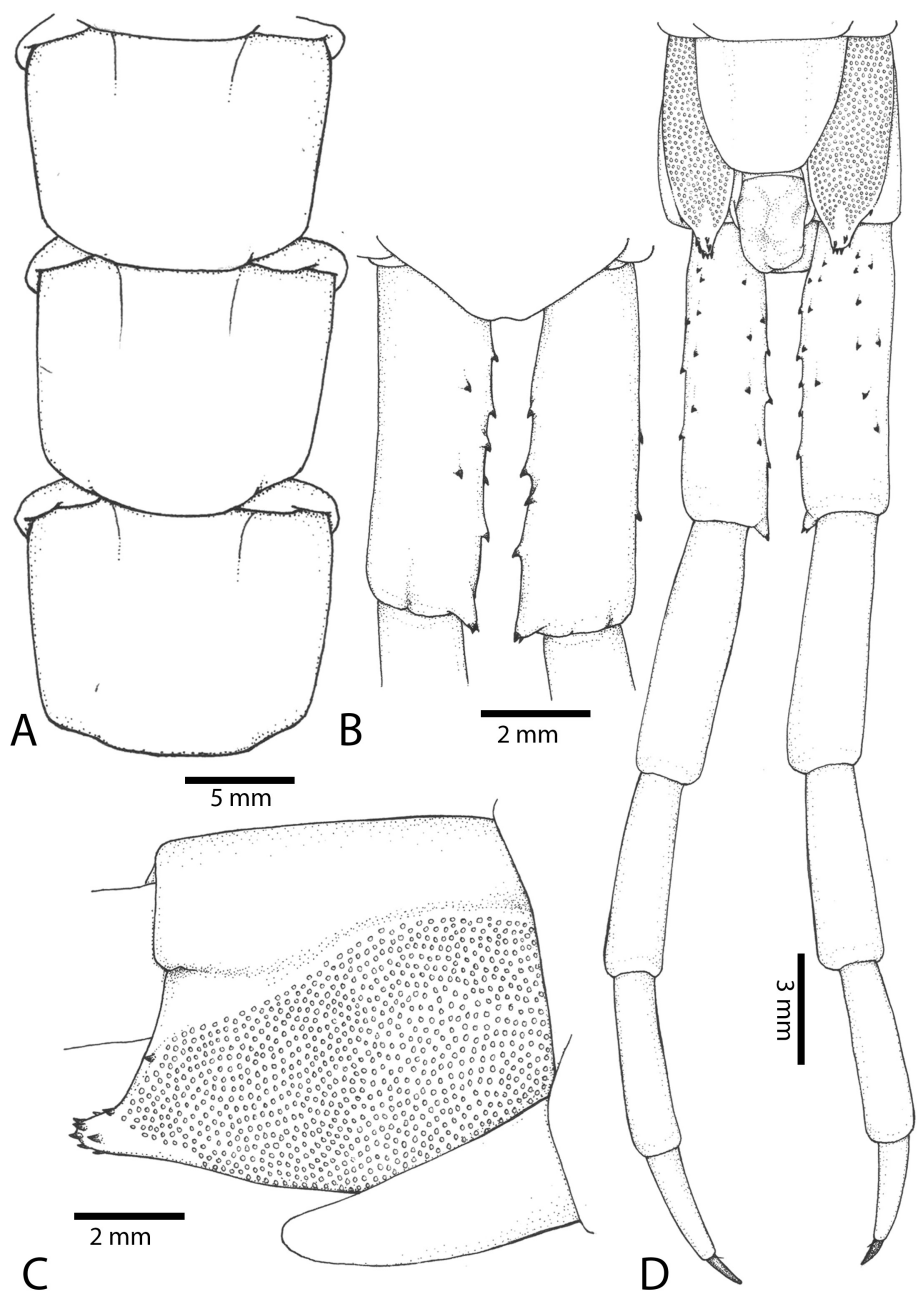
*Scolopendra pinguis* (CUMZ 00306, 00314): **A** Sternites 9–11 **B** Dorsal view of ultimate leg prefemora **C** Lateral view of coxopleuron **D** Sternite of ultimate leg-bearing segment, coxopleura and ultimate legs.

Anterior margin of T1 underlying cephalic plate (Figs 43A, 44A). Complete paramedian sutures from T3; margination typically from TT16–18 (atypically, only on tergite of ultimate leg-bearing segment in one specimen: CUMZ 00311). Tergite surface (Figs 43B, 46C) smooth, without median sulci. Tergite of ultimate leg-bearing segment (Figs 45B, 46E) curved and acute posteriorly, without median furrow or depression; ratio of width: length of tergite of ultimate leg-bearing segment 0.82:1. Anterior part of sternites (Figs 44C, 45A, 47A) with short paramedian sutures reaching approximately 20–30% length of sternite (atypically to 60%). Surface of sternites smooth. Sternite of ultimate leg-bearing segment (Fig. 45D–E) with sides converging posteriorly; without depression. Pore-field on coxopleuron reaching to margin of tergite of ultimate leg-bearing segment, dorsal margin of pore field sinuous (Figs 45C, 47C).

Coxopleural process moderately long or short with 3 apical, 0–3 subapical, 0–1 dorsal and 1 lateral spine(s). Pore-free area extending 30–45% length from distal part of coxopleural process to margin of sternite of ultimate leg-bearing segment (Figs 45D–E, 47D).

All legs with small setae on tarsus 2. Tibial spur absent on all legs. One tarsal spur on legs 1–19(20). Ultimate legs: thick and moderately long (Fig. 47D), with ratios of lengths of prefemur and femur 1.3:1, femur and tibia 1:1; tibia and tarsus 2 1.2:1; tarsus 1 and tarsus 2 2.2:1. Prefemora and femora flattened posteriorly, with robust or acute blackish prefemoral spines. Prefemoral spines (Figs 45F–G, 47B, 47D): 6–12 VL, 1–12 VM, 2–3 M, 3–4 DM, prefemoral process with 3–4(7) spines. Posterior margin of prefemur with short median groove

Genital segments well developed, reaching longer than distance between posterior margin of sternite of ultimate leg-bearing segment and distal part of coxopleural process. Sternite of genital segment 1 round and convex posteriorly, with median suture (Fig. 25C). Gonopod present in male (Figs 7E, 25C). Lamina subanalis situated between genitalia and anal valve; lamina analis between anal valve and tergite of genital segment (Fig. 7F). In male, tergite and sternite of genital segment and gonopods with small setae.

**Colouration.** According to Siriwut et al. (2015a), *Scolopendra pinguis* exhibits four colour morphs which can classified as either monochromatic or dichromatic (Fig. 42A–B). All of these patterns are specific to populations, with no mixing or sympatry found in our surveys. The full description of each colour morph is given below:

**Colour morph 1A**: Monochromatic, all segments including cephalic plate dark blue. Antenna dark blue on basal part, light blue on distal part. Pleuron with pale blue integument, all pleurites black. All legs blue, legs 19–21 dark blue. This morph has been found only in Thailand.

**Colour morph 1B**: Monochromatic, all segments including cephalic plate dark blue. Antenna dark blue on basal part, light blue on distal part. Pleuron with pale blue integument, all pleurites black. Most legs yellowish on prefemur, other podomeres dark blue with yellowish band bordering articulations; last three legs dark blue or black. This morph has been found only in Thailand.

**Colour morph 2A**: Dichromatic, cephalic plate dark blue on anterior part, yellowish on posterior part and T1. Antenna dark blue on basal part, light blue on distal part. Pleuron with pale blue integument, all pleurites black. All legs dark blue or black. This morph has been found both in Thailand and North-Central Laos.

**Colour morph 2B**: Dichromatic, cephalic plate dark blue on anterior part, yellowish on posterior part and T1. Antenna dark blue on basal part, light blue on distal part. Pleuron with pale blue integument, all pleurites black. All legs yellowish on prefemur, other podomeres light blue with yellowish band bordering articulations. This morph has been found both in Thailand and North-Central Laos.

**Discussion.** *Scolopendra pinguis* has not been revised since Pocock (1891) described the holotype from Burma. Two additional records from Batavia-Buitenzorg (Bogor, Java) expanded its geographical distribution across Southeast Asia (Pocock 1894). Kraepelin (1903) confirmed additional material from Buitenzorg; Bogor, Java. Attems’ (1930b) monograph followed Kraepelin’s description and argued that this species is similar to another Javan species, *Scolopendra gracillima*. This argument has been followed in several subsequent taxonomic reviews (Schileyko 1995, 2007, Lewis 2010b). Comparative taxonomic characters of these two species (Table 9) can, in the present state of knowledge, be used to defend species validity until further data (e.g., molecular data for both species from Java) can be considered. Another related species from northwestern India, *Scolopendra ellorensis* Jangi & Dass, 1984, was also noted to be morphologically similar to *Scolopendra pinguis*. However, this Indian species was decribed from one juvenile specimen (31 mm) that might not permit confident comparison (Lewis 2010b). Molecular phylogenetic analysis of *Scolopendra pinguis* revealed a high level of genetic divergence among populations that might suggest regional endemism and the possibility of cryptic species. The latter would be consistent with the marked degree of colour polymorphism noted above.

**Table 9.** Morphological comparison of *Scolopendra pinguis*, *Scolopendra gracillima* Attems, 1898 and *Scolopendra calcarata*. ? Character not present or insufficient data.

Characters	*Scolopendra pinguis*			*Scolopendra gracillima*		*Scolopendra calcarata*		
	Pocock (1891, 1893)	Kraepelin (1903) and Attems (1930)	This study	Attems (1930)	Schileyko (1995)	Porat (1876)	Schileyko (1995)	This study
Number of antennal articles	17	17	17	17	17	17	17	17
Number of glabrous articles	3	3	4	5	6	4	5–6	4
Teeth on tooth-plate	?	?	6+6	5+5	4+4, 5+5	10–12 (in total)	5+5, 6+6	5+5
First tergite with complete paramedian sutures	T3	T3	T3	?	T3	?	T3	T3
First tergite with margination	20–21	20–21	T16–18	20–21	only 21	T12–14	TT15–21	only T21
Tergite surface	?	?	smooth	punctate on TT3(4)–19(20)	?	smooth	?	smooth
Median furrow on tergite of ULBS	absent	absent	absent	absent	absent	present	absent	absent
Extent (percentage) of paramedian sutures on sternites	nearly complete on anterior part of body	nearly complete on anterior part of body	incomplete (20–30%)	incomplete	complete only on anterior part of body	?	incomplete	incomplete (20–50%)
Sternite of ultimate leg-bearing segment	wide, sides converging	wide, sides converging	sides converging posteriorly	narrow, posterior margin rounded	narrow, posterior margin rounded	?	trapeziform with shallow depression	sides converging
Spines on coxopleural process	AP+SAP: 5 L: 1	AP: 3 SAP: 0–3 LS: 1 DS: 1	AP: 3 SAP: 0–3 LS: 1–2 DS: 0–1	AP+SAP: 3–5 LS: 1	AP+SAP: 5–6 LS: 1	AP+SAP: 5	AP+SAP: 5 LS: 1 DS: 1	AP: 3–4 SAP: 0–3 LS: 1 DS: 1
Spine formula on prefemora of ult. legs	V: 14–22 D: 2–8	V: 14–22 D: 2–8 (5–8)	VL: 6–12 VM: 1–12 M: 2–6 DM: 3–4	VL: 6–8 VM: 3–4 M: 3–4 D: 1–3 SP:4–5	VL: 7 VM: 5–6 D: 3–4 SP: 4	V: 9 M: 5 D: 7 SP: 5	VL: 9–12 VM: 11–12 M: 2–3 D: 2–3	VL: 4–7 VM: 0–3 M: 1–2 DM+SP: 3–4
Legs with one tarsal spur	1–20 (?)	1–19	1–19 (20)	1–20	1–20	1–21	1–21	1–21

##### Distribution.

A native species in Southeast Asia, distributed along the montane ranges between the Thailand-Burma borders (Fig. 29). The updated distribution of *Scolopendra pinguis* is as follows: **Southeast Asia**: Myanmar (type locality), Thailand (Kanchanaburi, Mae Hong Son, Chiang Mai, Phayao, Chiyaphume and Loei), Laos (Bo Kaew, Luang Prabang, Vientien and Houaphanh) and Indonesia (Batavia, Buitenzorg [Bogor], Java).

#### Scolopendra dawydoffi

Taxon classificationAnimaliaScolopendromorphaScolopendridae

Kronmüller, 2012

Figs 7B , 29 , 42C , 48 , 49 , 50 , 51 , 52 , 53

Scolopendra subspinipes cingulatoides Attems, 1938: 335, fig. 307, 1953: 138. Schileyko 1998: 268, 2001: 434, 2007: 76. Lewis 2010b: 112, fig. 24. Tran et al. 2013: 229.
Scolopendra dawydoffi Kronmüller, 2012: 22, table 1, fig. 4E [new replacement name]. Tran et al. 2013: 229. Siriwut et al. 2015a: 1.

##### Type locality.

Two localities were reported in the original description, Hagiang, Haut Tonkin [Hà Giang Province, northern Vietnam], and Thakek, Laos [Thakhek, Khammouane Province, Laos].

##### Material.

**Syntypes** NHMW Inv. No. 8234, two females labeled “*Scolopendra subspinipes cingulatoides* Attems, 1934 typus by Attems”, Thakek, Laos (Figs 50, 51).

**Figure 48.**
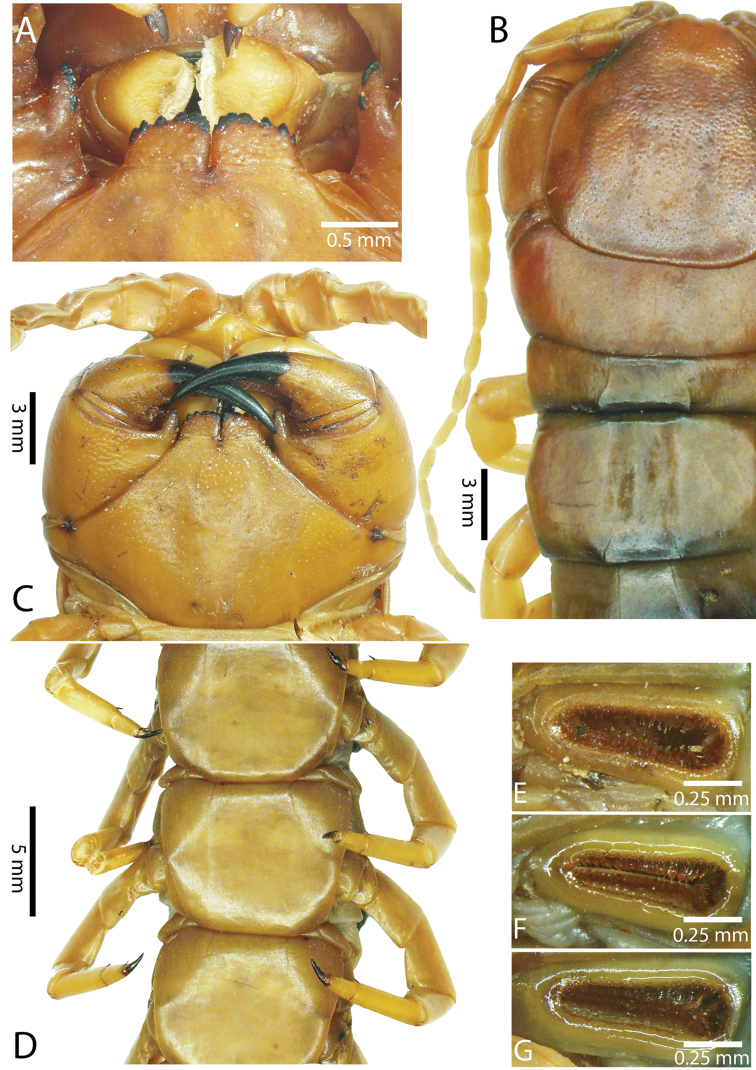
*Scolopendra dawydoffi* (CUMZ 00290, 00291): **A** Tooth-plates **B** Forcipular segment **C** Cephalic plate and trunk segments 1–3 **D** Sternites 9–11 **E–G** Spiracles 3, 5 and 8, respectively.

**Figure 49.**
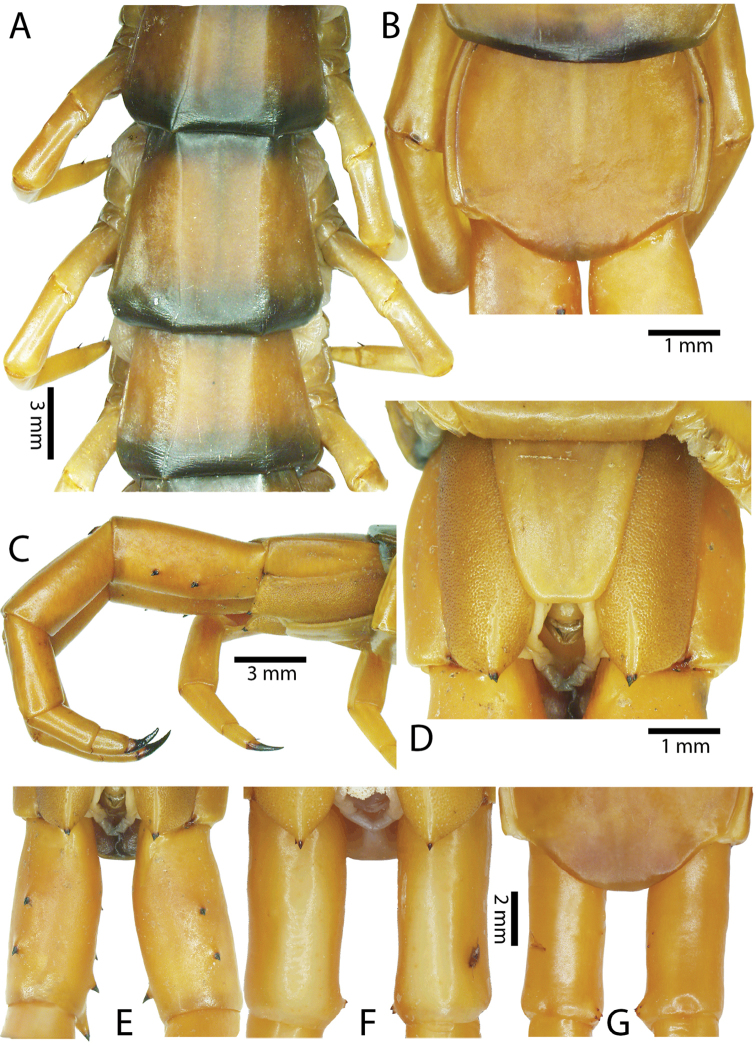
*Scolopendra dawydoffi* (CUMZ 00290, 00291, 00272): **A** Tergites 9–11 **B** Tergite of ultimate leg-bearing segment **C** Lateral view of coxopleuron, leg 20 and ultimate leg **D** Sternite of ultimate leg-bearing segment and coxopleura **E** and **F** Variation in ventral spines on ultimate leg prefemora **G** Dorsal view of ultimate leg prefemora.

**Figure 50.**
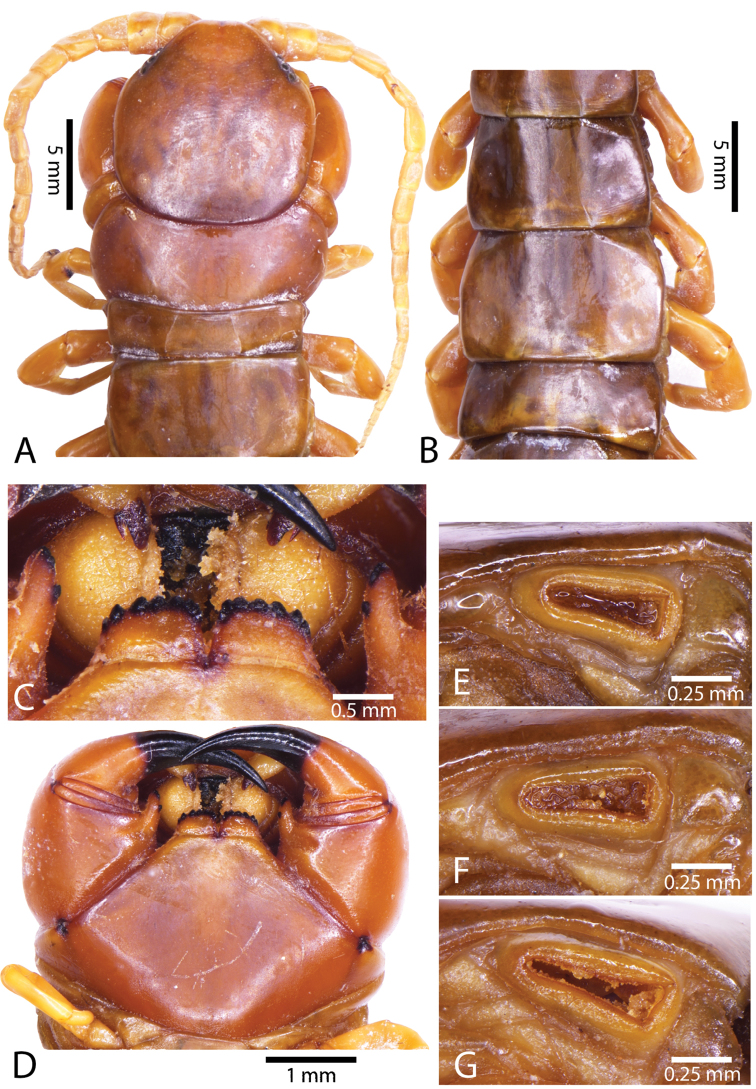
*Scolopendra dawydoffi* (Syntypes NHMW 8234): **A** Cephalic plate and trunk segments 1–3 **B** Tergites 9–11 **C** Tooth-plates **D** Forcipular segment **E–G** Spiracles 3, 5 and 8, respectively.

**Figure 51.**
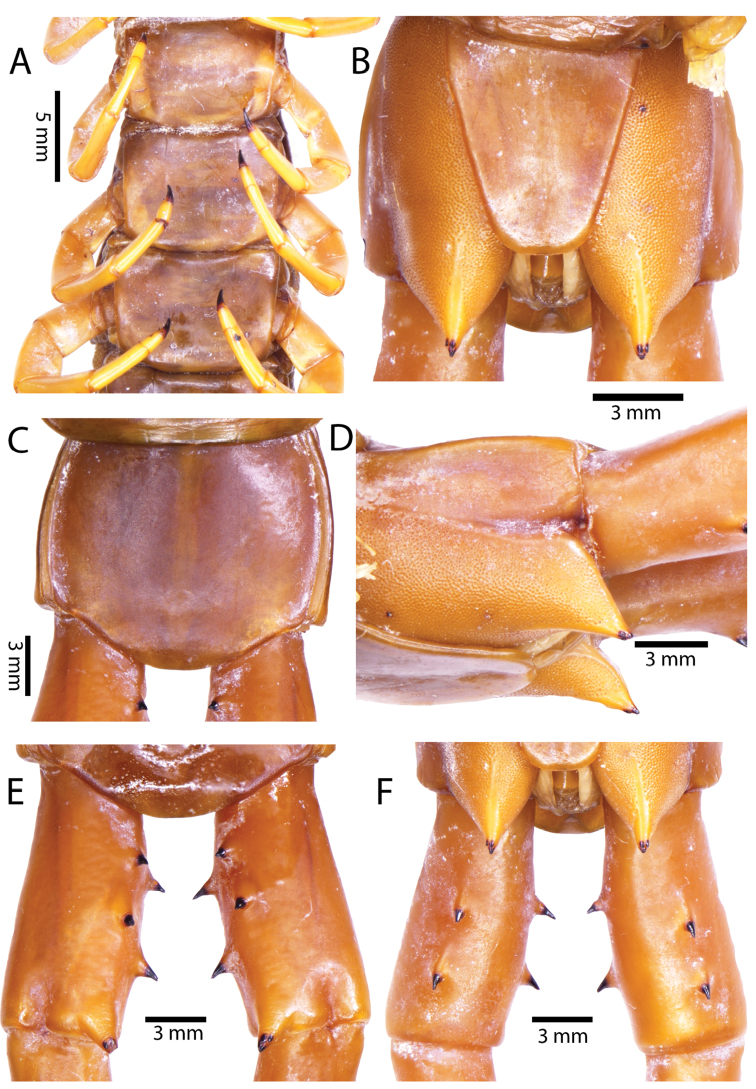
*Scolopendra dawydoffi* (Syntypes NHMW 8234): **A** Sternites 9–11 **B** Sternite of ultimate leg-bearing segment and coxopleura **C** Tergite of ultimate leg-bearing segment **D** Lateral view of coxopleuron **E–F** Dorsal and ventral view of ultimate leg prefemora.

##### Additional material.

**Thailand** — ZMUC 1/7.59, one adult female and 13 juvenile spms., labeled as “*Scolopendra subspinipes*”, Phu Kradueng, Loei, 1300 m, evergreen forest, 24/11/1958, leg. B. Degerbol. CUMZ 00294 two adult spms., Wat Thang Biang, Pak Chong, Nakhon Ratchsrima (14°32'22.0"N, 101°21'54.6"E). CUMZ 00290, one spm., Sakaerat Biosphere Reserve Center, Nakon Ratchasima (14°30'36.5"N, 101°55'51.5"E). CUMZ 00272.1–2, two spms., Saphan Hin Waterfall, Khlong Yai, Trad (12°06'06.0"N, 102°42'39.2"E)

**Malaysia** — NHMUK.1950.4.19.12, one spm., Kelantan, Malay Peninsula (labeled as “*Scolopendra subspinipes*”).

##### Diagnosis.

17–18 antennal articles, 6 basal articles glabrous dorsally. Each tooth-plate with 5–10 teeth. Tergites 2(3)-20 with paramedian sutures. Complete tergite margination from TT11–21. Tergite of ultimate leg-bearing segment without depression or suture. Paramedian sutures on anterior 15–60% of sternites. Coxopleural process with 2–3 apical+subapical spines. Ultimate leg prefemora with 1–2 VL, 0–2 M, 0–2 DM, prefemoral process with 1–5 spines. One tarsal spur on legs 1–19.

##### Composite description.

Body length up to 16.2 cm (14.7 and 15.1 cm in syntypes). Reddish colouration on entire body. Cephalic plate and tergites dichromatic. Cephalic plate and tergites reddish orange; posterior border of tergites with dark band. Cephalic plate with small punctae; median sulcus present. Posterior part of cephalic plate without paramedian sulci.

**Figure 52.**
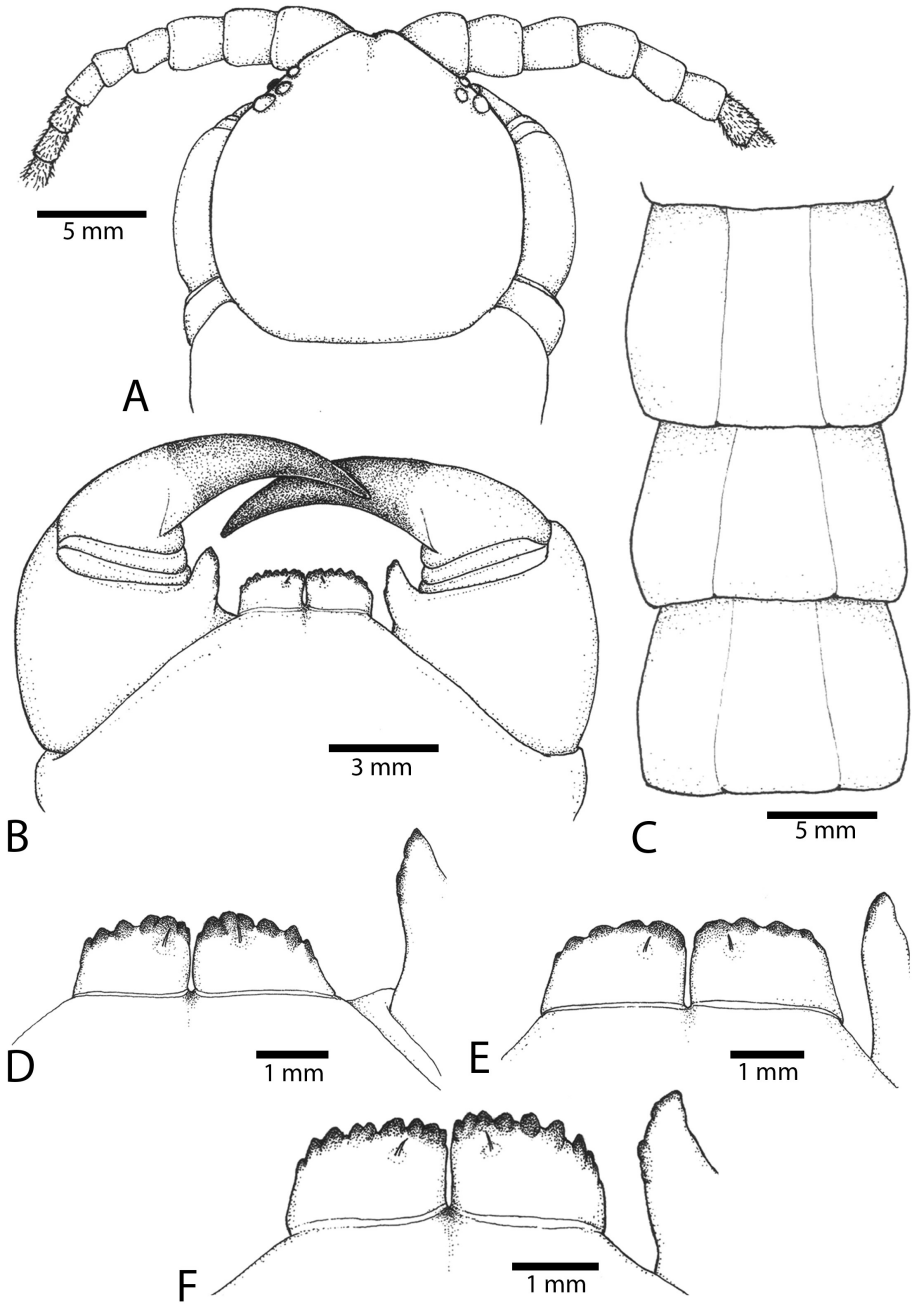
*Scolopendra dawydoffi* (CUMZ 00272): **A** Cephalic plate and basal antennal articles **B** Forcipular segment **C** Tergites 9–11 **D–F** Variation in teeth on tooth-plates and trochanteroprefemoral process.

Antenna usually with 18 articles (sometimes 17 on one side in some specimens), basal 6 subcylindrical and glabrous dorsally (Fig. 52A), 5–5.5 articles glabrous ventrally. Antennae reach segment 4. Forcipular trochanteroprefemoral process with denticles in two groups, one apical and 2–3 inner. Anterior part of coxosternite with tooth-plates, wider than long or nearly equivalent, 5–7 robust teeth (Figs 48A, 52D–F); atypically with 10 teeth (CUMZ 00272). Tooth-plate with straight, transverse suture. Coxosternal surface smooth, without median suture (Figs 48B, 52B). Article 2 of second maxillary telopodite with spur.

**Figure 53.**
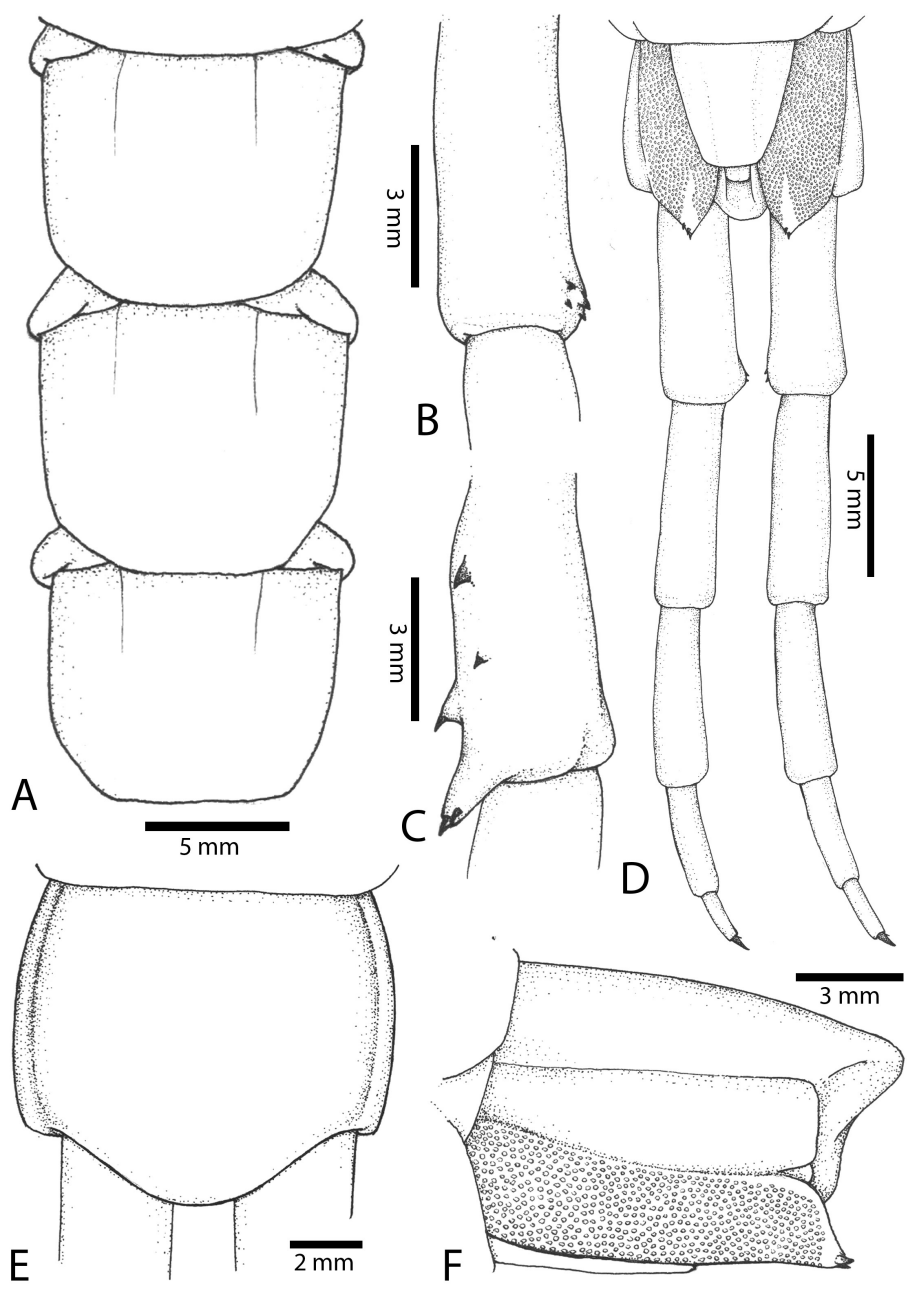
*Scolopendra dawydoffi* (CUMZ 00272): **A** Sternites 9–11 **B–C** Variation in numbers of spines on prefemural process of ultimate leg **D** Sternite of ultimate leg-bearing segment, coxopleura and ultimate legs, showing lack of ventral and median spines on prefemora **E** Tergite of ultimate leg-bearing segment **F** Lateral view of coxopleuron.

Anterior margin of T1 underlying cephalic plate (Fig. 48C). Complete paramedian sutures on TT2–3; margination typically from TT11–14. Tergite surfaces (Figs 49A, 52C) smooth, without median sulci. Tergite of ultimate leg-bearing segment (Figs 49B, 53E) curved posteriorly, without median furrow or depression; ratio of width: length of tergite of ultimate leg-bearing segment 0.7:1. Anterior part of sternites (Figs 48D, 53A) with short paramedian sutures reaching approximately 15–30% (atypically, to 60% in one specimen: CUMZ 00272). Surface of sternites smooth, mostly with depression (small circular pit present on posterior median part of sternite in one specimen: CUMZ 00294). Sternite of ultimate leg-bearing segment (Fig. 49D) with sides converging posteriorly. Pore-field on coxopleuron terminating beneath margin of tergite of ultimate leg-bearing segment, dorsal margin of pore area sinuous, most elevated anteriorly.

Coxopleural process moderately long or short with two apical spines and one subapical spine (atypically only two apical spines; Fig. 53F); pore-free area extending 65–90% length from distal part of coxopleural process to margin of sternite of ultimate leg-bearing segment (Figs 49D, 53D).

All legs without setae and tibial spur. One tarsal spur on legs 1–19. Ultimate legs: thick and moderately long (Figs 49C, 53D), with ratios of lengths of prefemur and femur 1.3:1, femur and tibia 1.3:1, tibia and tarsus 2 1.8:1, tarsus 1 and tarsus 2 1.7:1. Prefemora flattened dorsally (atypically rounded; Figs 49F–G, 53B), with robust blackish spines. Prefemoral spines (Figs 49E, 51E–F): 1–2 VL, 0–2 M, 0–2 DM, prefemoral process with 1–3 spines, atypically with 5 spines (Figs 49G, 53B). Posterior margin of prefemur with shallow median groove

Genital segments well developed, reaching longer than distance between posterior margin of sternite of ultimate leg-bearing segment and distal part of coxopleural process. Sternite of genital segment 1 round and convex posteriorly, with median suture (Fig. 28C–D). Sternite of genital segment 2 developed. Gonopod absent in male. Lamina subanalis between genitalia and anal valve; lamina analis between anal valve and tergite of genital segment. Tergite and sternite of genital segments with small setae. Penis with apical bristle.

##### Discussion.

This species is distinguished from *Scolopendra subspinipes* by its short, robust ultimate legs and three apical/subapical spines on the coxopleural process. The characteristic of incomplete paramedian sutures on the sternites further distinguishes it from *Scolopendra subspinipes* and *Scolopendra japonica* (which have complete paramedian sutures on the sternites). However, *Scolopendra dawydoffi* is similar to *Scolopendra multidens* in the absence of gonopods in the male. The distribution of *Scolopendra dawydoffi* is restricted to mainland Southeast Asia whereas *Scolopendra multidens* occurs in temperate regions of Asia, including both inland and insular parts. A specimen identified as *Scolopendra multidens* from Vietnam is genetically differentiated from Thai populations (see discussion of *Scolopendra multidens* above for molecular arguments in favour of the two taxa being separate species). Moreover, to test the hypothesis that characteristics of *Scolopendra dawydoffi* might indicate affinities to the *cingulata* group (with reference to the Mediterranean species *Scolopendra cingulata*; Attems 1930a), as implied by the original “*cingulatoides*” name for *Scolopendra dawydoffi*, our phylogenetic analysis included *Scolopendra cingulata* sequences from Spain. The result (Fig. 1) demonstrated that *Scolopendra dawydoffi* was not grouped together with *Scolopendra cingulata* but should be recognized as a distinct species based on its genetic distance and geographical distribution. A morphological comparison between these two species is presented in Table 10.

**Table 10.** Morphological comparison of *Scolopendra dawydoffi* and *Scolopendra multidens*; data from present study and previous taxonomic studies, i.e., Attems (1938), Chao (2008), Kronmüller (2012). ? Character not present, L left side.

Characters	*Scolopendra dawydoffi*		*Scolopendra multidens*	
	Laos (Syntypes)	Thailand	China (Holotype)	Hong Kong and Taiwan
Number of antennal articles	18	17–18	7/4, damaged	17–19
Number of glabrous articles	6	6	6	6
Teeth on tooth-plate	6+6	5+5, 7+6, 10+9	5+7	7+7
First tergite with complete paramedian sutures	3	2–3	2	2–4
First tergite with margination	11	12–14	9	13
Tergite surface	smooth	smooth	short median furrow on posterior part	short transverse groove on anterior-lateral part (TT2–20)
Median furrow on tergite of ULBS	absent	absent	absent	absent
Extent (percentage) of paramedian sutures on sternites	incomplete (15–35%)	incomplete (20–60%)	incomplete (40–60%)	incomplete (20–100%)
Sternite of ultimate leg-bearing segment	sides converging posteriorly	sides converging posteriorly	sides converging posteriorly	sides converging posteriorly
Spines on coxopleural process	AP: 1 SAP: 2	AP: 1–2 SAP: 1–2	AP: 1 SAP: 2	AP: 1–2 SAP: 1–2
Spine formula on prefemora of ultimate legs	VL: 2 M: 2 DM: 2 SP: 2–3	VL: 0–2 M: 0–1 DM: 0–2 SP: 1–5	VL: 3 M: 2 DM: 2 SP: 2–3	VL: 2 M: 2 DM: 2 SP: 1–3
Legs with one tarsal spur	1–19	1–19	1–19 (20?)	1–19 (20L)
Podomeres of ultimate legs with shallow groove on posterior part	prefemur and femur	prefemur and femur	prefemur and femur	prefemur and femur
Gonopods on genital segment	? (female)	absent	?	absent

##### Distribution.

This species was formerly reported from only two occurrences in Southeast Asia, one in each of Laos and Vietnam (Attems 1938). We add more material from Thailand and some adjacent areas based on museum collections. The updated distribution range (Fig. 29) is as follows: **Southeast Asia**: Thailand (Nakhon Ratchasima, Loei and Trad), Laos (Thakhek), Malaysia (Kelantan state) and Vietnam (fide Tran et al. 2013: Ha Giang, Hanoi, Thai Nguyen and Ha Tinh).

#### Scolopendra cataracta

Taxon classificationAnimaliaScolopendromorphaScolopendridae

Siriwut, Edgecombe & Panha sp. n.

http://zoobank.org/0956CB47-120F-4D98-BEA4-1FBD63713281

**Figure 54.**
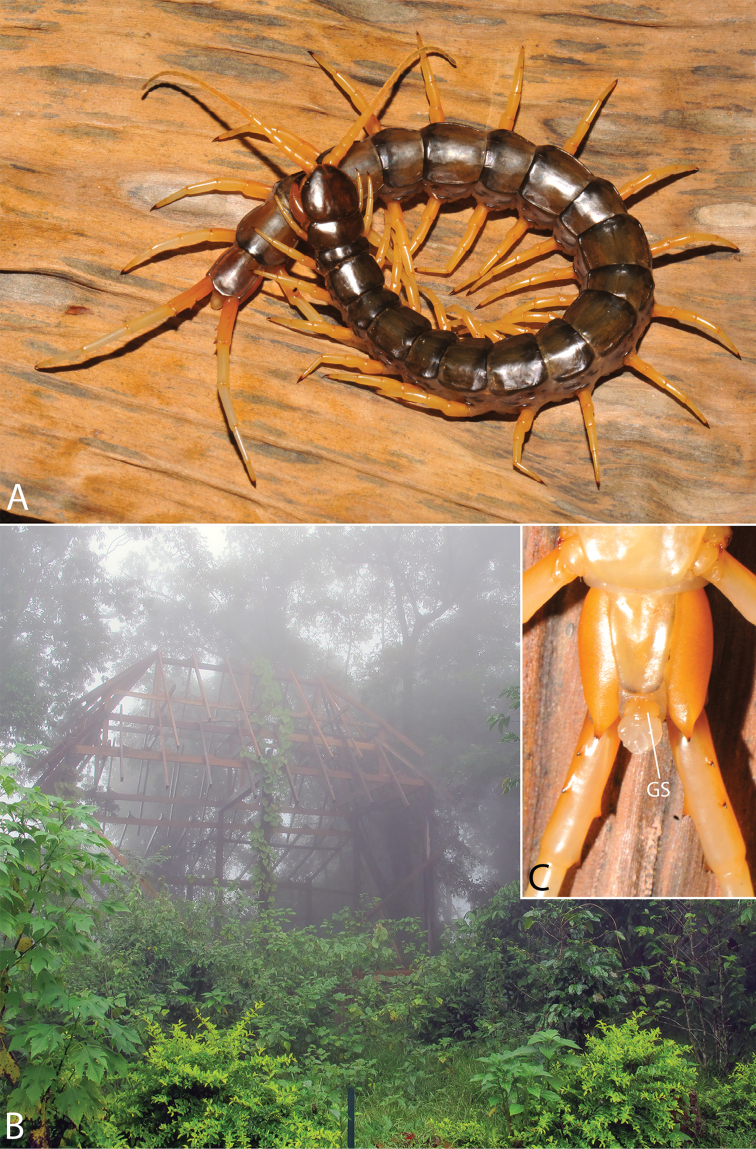
*Scolopendra cataracta* sp. n.: **A** Habitus photograph of holotype CUMZ 00316 **B** Habitat at type locality **C** Genital region; GS – Genital segment.

**Figure 55.**
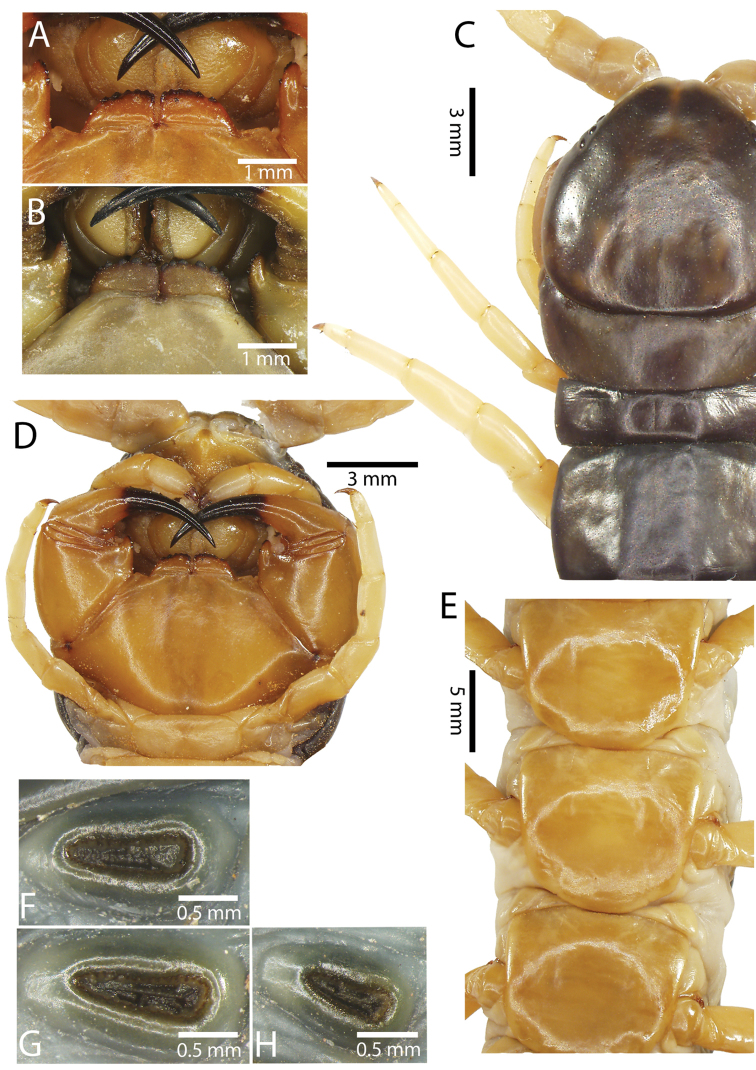
*Scolopendra cataracta* sp. n.: **A–B** Tooth-plates **C** Cephalic plate and trunk segments 1–3 **D** Forcipular segment **E** Sternites 9–11 and **F–H** Spiracles 3, 5 and 8, respectively. Holotype CUMZ 00316 (**A,C–H**) and paratype CUMZ 00317 (**B**).

**Figure 56.**
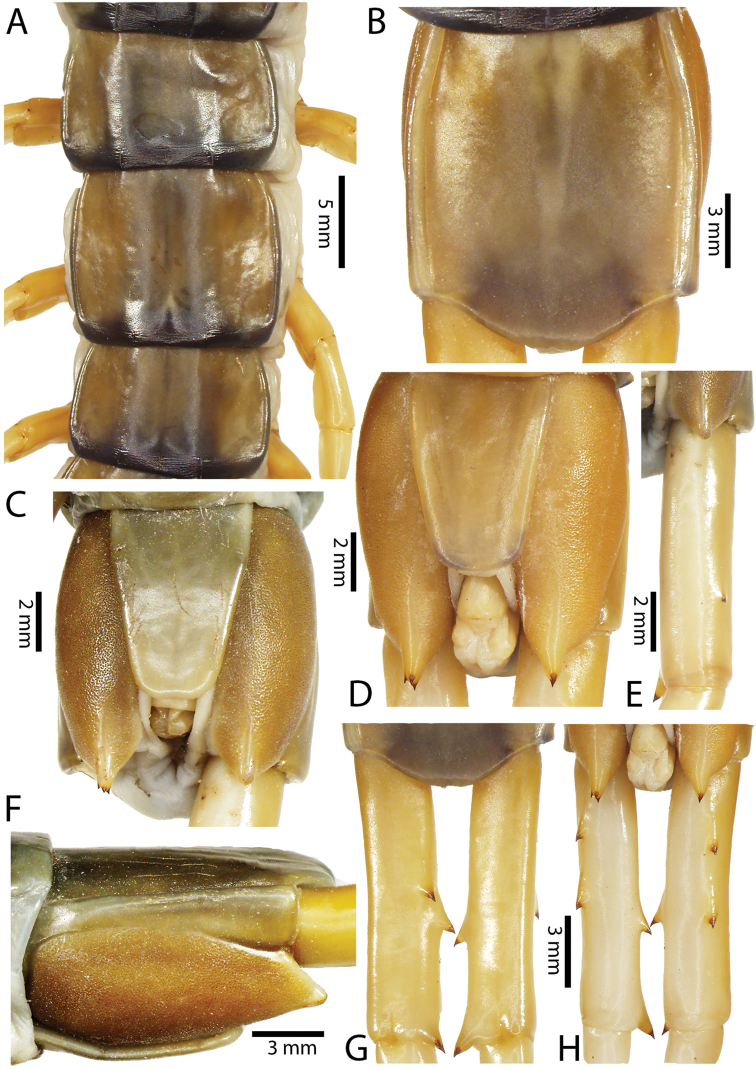
*Scolopendra cataracta* sp. n.: **A** Tergites 9–11 **B** Tergite of ultimate leg-bearing segment **C–D** Sternite of ultimate leg-bearing segment and coxopleura **E–H** Variation in ventral spines on ultimate leg prefemora **F** Lateral view of coxopleuron **G** Dorsal view of ultimate legs. Holotype CUMZ 00316 (**A, B, D, G** and **H**) and paratype CUMZ 00317 (**C, E** and **F**).

**Figure 57.**
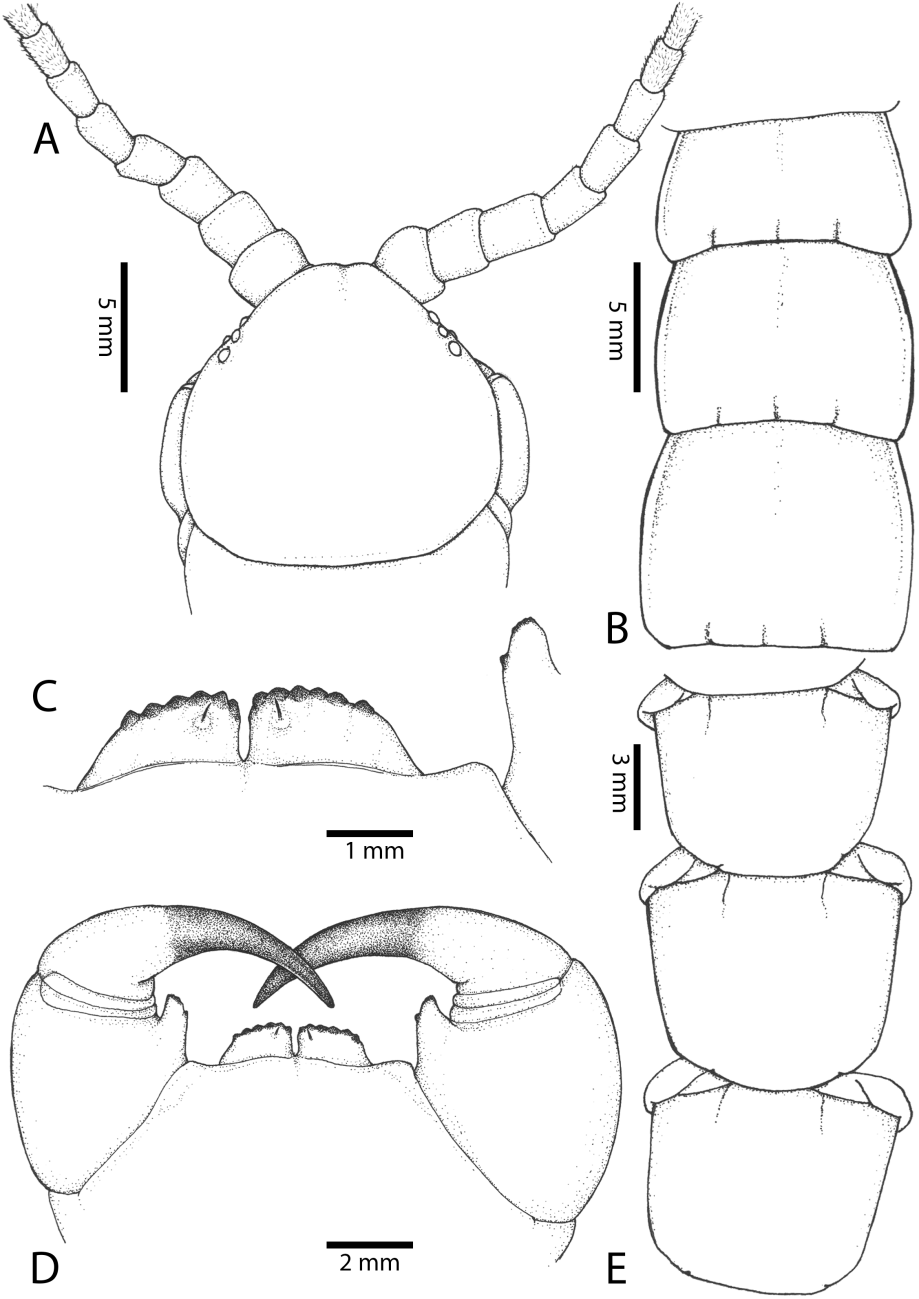
*Scolopendra cataracta* sp. n. (Holotype: CUMZ 00316): **A** Cephalic plate and basal antennal articles **B** Tergites 9–11 **C** Tooth-plates and trochanteroprefemoral process **D** Forcipular segment **E** Sternites 9–11.

**Figure 58.**
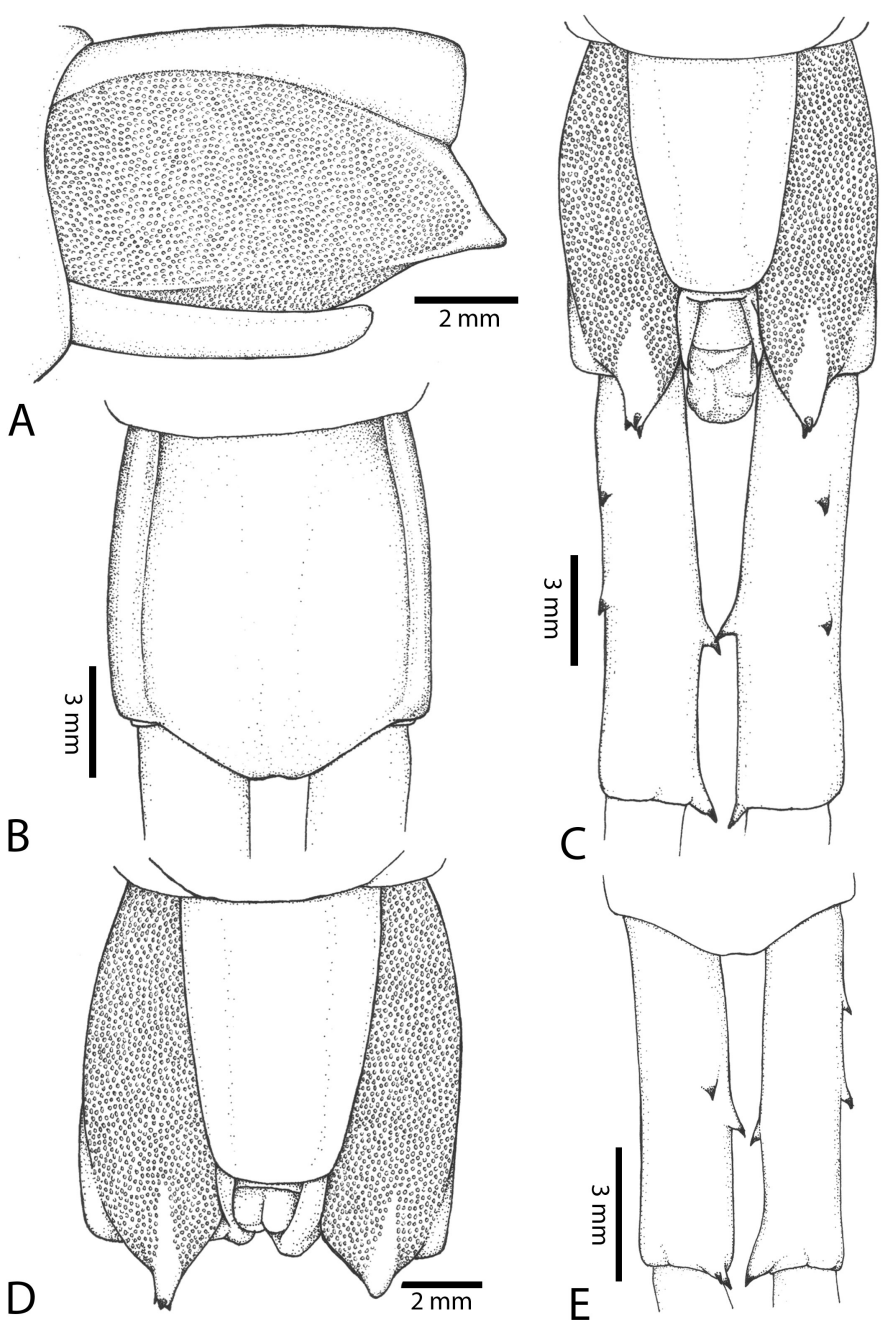
*Scolopendra cataracta* sp. n.: **A** Lateral view of coxopleuron **B** Tergite of ultimate leg-bearing segment **C** Sternite of ultimate leg-bearing segment, coxopleura and ultimate leg prefemora. **D** Asymmetry of spines on coxopleural process **E** Dorsal view of ultimate leg prefemora. Holotype CUMZ 00316 (**B–C, E**) and paratype CUMZ 00317 (**A, D**).

Figs 28C–D , 29 , 54 , 55 , 56 , 57 , 58

##### Type locality.

Tad E-tu Waterfall, Bolaven Plateau, Pakse, Champasak, Laos (15°13'10.6"N, 105°55'31.3"E) (Fig. 54B).

##### Materials.

**Holotype** CUMZ 00316, adult specimen from type locality (Fig. 54A). **Paratypes** CUMZ 00317, one adult, Tad-Yueang Waterfall, Mueang Singh, Luang Namtha, Laos (15°09'55.1"N, 106°06'10.6"E). NHMUK 010305528, one adult, Kao Sok National Park, Surat Thani, Thailand, labeled as “a distinct population of *Scolopendra subspinipes* but without paramedian sutures on the tergites”, det. J.G.E. Lewis, leg. G. Beccaloni, 2001. NHMUK 1928.5.30.7, one adult, Bac-Kan, Vietnam, labelled as “*Scolopendra subspinipes*”. NHMUK 1928.5.30.6, one female, Dac-To, Annam [Vietnam], Delacour-Lowe expedition.

##### Etymology.

From “*cataract*”, meaning waterfall, for the type locality at Tad E-tu Waterfall.

##### Diagnosis.

18–19 antennal articles, 6 basal articles glabrous dorsally. Cephalic plate punctate. 5–6 teeth on tooth-plate. Tergites 7(14)-20 with paramedian sutures, all incomplete, present only on anterior and posterior parts. Tergite of ultimate leg-bearing segment without depression or suture. Paramedian sutures confined to anterior 15–20% of sternites. Coxopleural process with 1–3 apical+subapical spines, 0–1 dorsal spine, without lateral spine. Ultimate leg prefemora with 1–2 VL, 1–2 M, 0–2 DM and prefemoral process with 1–3 spines. Tarsal spur on legs 1–19(20).

##### Holotype description

**(variation of paratypes is given in parentheses).** Body length 12.8 cm (up to ca. 20 cm long in paratype NHMUK 1928.v.30.6). Blackish colouration on entire body. Cephalic plate and segments monochromatic. Tergites dark greenish or black. Cephalic plate without small punctae on anterior part; median sulcus present. Posterior part of cephalic plate without paramedian sulci.

Antenna usually with 19 articles (atypically with 18 articles on one side), basal 6 glabrous dorsally (Fig. 57A), 5–5.5 glabrous ventrally. Antennae reach segment 4(5). Forcipular trochanteroprefemoral process with denticles in two groups, one apical tooth and (2)3–4 inner (Fig. 57C). Tooth-plates (Fig. 55A–B) wider than long or nearly as long as wide, with 6 teeth (5 in NHMUK 1928.5.30.6). Tooth-plate with straight, transverse basal suture. Coxosternal surface smooth, without median suture (Figs 55D, 57D). Article 2 of second maxillary telopodite with spur.

Anterior margin of T1 underlying cephalic plate (Fig. 55C). Incomplete paramedian sutures on all tergites; margination typically starting on T7 (TT10–14). Tergite surface (Figs 56A, 57B) smooth, with median sulci starting from TT5–20. Tergite of ultimate leg-bearing segment (Figs 56B, 58B) curved posteriorly, without median furrow or depression; ratio of width: length of tergite of ultimate leg-bearing segment 1.1:1. Anterior part of sternites (Figs 55E, 57E) with short paramedian sutures confined to approximately 20–45% length of sternite. Surface of sternites smooth. Sternite of ultimate leg-bearing segment (Figs 56C–D, 58C–D) with sides converging posteriorly; surface without depression. Pore-field on coxopleuron extending to and overlapped by margin of tergite of ultimate leg-bearing segment, dorsal margin of pore area sinuous, most elevated anteriorly (Figs 56F, 58A).

Coxopleural process (Fig. 56C–D) moderately long or short with 2–3 apical+subapical spines (spines atypically absent on one side; CUMZ 00317), without lateral spine (one paratype with one dorsal spine on one side: CUMZ 00317). Pore-free area extending 30–90% length from distal part of coxopleural process to margin of sternite of ultimate leg-bearing segment (Figs 54C, 56C–D and 58C–D.).

All legs without setae and tibial spur. One tarsal spur on legs 1–20 (1–19 in one paratype, CUMZ 00317). Ultimate leg: long and slender, with ratios of lengths of prefemur and femur 1.1:1, femur and tibia 1.1:1, tibia and tarsus 2 1.3:1; tarsus 1 and tarsus 2 1.9:1. Prefemora flattened dorsally with acute blackish spines. Prefemoral spines (Figs 56G–H, 58C, 58E): 2 VL, 1 M, 0–1 DM, prefemoral process with 2 spines (Paratypes: 1–2 VL, 1–2 M, 0–2 DM and prefemural process with 1–3 spines).

Genital segments well developed (Figs 28C–D, 54C), reaching longer than distance between posterior margin of sternite of ultimate leg-bearing segment and distal part of coxopleural process. Sternite of genital segment 1 round and convex posteriorly, with median suture. Tergites of genital segment lacking small setae. Presence of gonopods and penis uncertain because genitalia are mostly retracted in holotype and paratypes; only two female specimens show genital segments (NHMUK 010305528 and 1928.5.30.6).

##### Remarks.

The paratype collected in Thailand in 2001 (NHMUK 010305528) was observed to display apparent amphibious habits. The following account is based on observations by G. Beccaloni (pers. comm., Jan. 2016). The centipede was initially observed under a rock slab beside a stream ca. 1.5 m wide and 20 cm deep. It escaped into the stream and concealed itself under a rock. After extraction from the stream it was placed in a glass container of water, in which it swam powerfully on the bottom of the container with vigorous horizontal undulating motions.

##### Discussion.

This species exhibits an atypical characteristic for scolopendromorphs, namely incomplete paramedian sutures on the tergites. Very few members in only three genera share this character, these being within *Scolopocryptops* Newport, 1844, *Scolopendra* and *Rhysida* Wood, 1862. Within *Scolopendra*, only two described species, *Scolopendra hainanum* from Hainan Island, China, and “*Scolopendra subspinipes piceoflava*” (treated above as a synonym of *Scolopendra subspinipes* following Kronmüller (2012)), from Sulawesi, Indonesia, have been reported to lack or have nearly absent paramedian sutures on the tergites. *Scolopendra cataracta* and “*Scolopendra subspinipes piceoflava*” can be distinguished from each other by: paramedian sutures confined to the posterior part of tergites in *Scolopendra cataracta* vs complete sutures in “*Scolopendra subspinipes piceoflava*”, very short paramedian sutures on the sternites vs sutures extending along 80–100% the length of the sternites, and three apical/subapical spines on the coxopleural process vs one or two.

*Scolopendra cataracta* differs from *Scolopendra hainanum* by the short paramedian sutures on the sternites versus being nearly complete in *Scolopendra hainanum*, the number of spines on the coxopleural process (three vs one or two), and the number of VL spines on the ultimate leg prefemora. The two species can also be distinguished by their colouration patterns and their distributions, though the latter are closely associated. For these reasons, we regard *Scolopendra cataracta* as distinct from *Scolopendra hainanum*, and its sampled populations group as monophyletic for each partial gene analysis (see Table 11 for morphological comparison). It is likewise morphologically distinct from *Scolopendra subspinipes*, *Scolopendra multidens* and *Scolopendra dawydoffi*. DNA sequences are not available for *Scolopendra hainanum* but would be useful to study the relationship between these two apparently related species.

**Table 11.** Morphological comparison of *Scolopendra cataracta* sp. n. and some related species in the adjacent sub-regions. ? Character not present, R right side, * data from Kronmüller (2012).

Characters	*Scolopendra cataracta* sp. n.				*Scolopendra subspinipes piceoflava* (Syntypes)	Scolopendra hainanum*
	Laos (Holotype)	Thailand (Paratype)	Vietnam (Paratypes)	Laos (Paratype)		
Number of antennal articles	19	19	19	18(19)	17–18	17–19
Number of glabrous articles	6	6	6	6	6	6
Teeth on tooth-plate	6+6	6+6	5+5, 6+6	6+6	5+5, 6+6	6+6,7+7
First tergite with complete paramedian sutures	incomplete (short PS on anterior and posterior part)	incomplete (short PS on posterior parts from TT5–20)	incomplete (short PS on anterior and posterior parts from TT3–20)	incomplete (short PS on anterior and posterior part)	complete, starting from T3	incomplete, starting from T3–4
First tergite with margination	7	14	7 and 10	12	5–7	5
Tergite surface	smooth	smooth	smooth	smooth	punctate	smooth
Extent (percentage) of paramedian sutures on sternites	15–20%	20–45%	10–25%	15%	complete	nearly complete
Sternite of ultimate leg-bearing segment	sides converging posteriorly	sides converging posteriorly	sides converging posteriorly, with pit like-furrow	sides converging posteriorly	sides converging posteriorly, with pit like-furrow	sides converging posteriorly, with pit like-furrow
Spines on coxopleural process	AP: 3(2) DP: 0–1	AP: 1–2	AP: 1 SAP: 2	AP: 3 DP: 1	AP: 1	AP: 1–2
Spine formula on prefemora of ultimate legs	VL: 2 M: 1 DM: 0–1 SP: 2	VL: 1 M: 0–1 DM: 1–2 SP: 1	VL: 2 M: 1–2 DM: 1–2 SP: 2–3	VL: 1 M: 0 DM: 0 SP: 2	V: 0–2 SP: 2–5	VL: 1 VM: 1 DM: 2 SP: 2
Legs with one tarsal spur	1–20	1–20	1–19, 20(R)	1–19	1–20	1–19
Gonopods on genital segment	female specimen	?	female specimens	?	present	absent

##### Distribution.

All localities are in mainland territory. The currently known distribution (Fig. 29) is as follows: **Southeast Asia**: Laos (Champasak and Luang Namtha), Thailand (Surat Thani) and Vietnam (Bac Kan and Dac-To).

## Supplementary Material

XML Treatment for Scolopendra morsitans

XML Treatment for Scolopendra subspinipes

XML Treatment for Scolopendra subspinipes piceoflava

XML Treatment for Scolopendra dehaani

XML Treatment for Scolopendra multidens

XML Treatment for Scolopendra calcarata

XML Treatment for Scolopendra japonica

XML Treatment for Scolopendra pinguis

XML Treatment for Scolopendra dawydoffi

XML Treatment for Scolopendra cataracta
